# Coupling Interrupted Fischer and Multicomponent Joullié‐Ugi to Chase Chemical Diversity: from Batch to Sustainable Flow Synthesis of Peptidomimetics

**DOI:** 10.1002/cmdc.202100474

**Published:** 2021-10-18

**Authors:** Antonella Ilenia Alfano, Elisabetta Buommino, Maria Grazia Ferraro, Carlo Irace, Angela Zampella, Heiko Lange, Margherita Brindisi

**Affiliations:** ^1^ SPOTS-Lab – Sustainable Pharmaceutical and Organic Technology and Synthesis Laboratory Department of Pharmacy University of Naples Federico II Via D. Montesano 49 80131 Naples Italy; ^2^ Department of Pharmacy University of Naples Federico II Via D. Montesano 49 80131 Naples Italy; ^3^ Current affiliation: Department of Environmental and Earth Science University of Milano-Bicocca Piazza della Scienza 1 20126 Milan Italy

**Keywords:** peptidomimetics, flow chemistry, multicomponent reactions, privileged scaffolds, sustainable synthesis

## Abstract

The generation of peptidomimetic substructures for medicinal chemistry purposes requires effective and divergent synthetic methods. We present in this work an efficient flow process that allows quick modulation of reagents for Joullié‐Ugi multicomponent reaction, using spiroindolenines as core motifs. This sterically hindered imine equivalent could successfully be diversified using various isocyanides and amino acids in generally good space‐time yields. A telescoped flow process combining interrupted Fischer reaction for spiroindolenine synthesis and subsequent Joullié‐Ugi‐type modification resulted in product formation in very good overall yield in less than 2 hours compared to 48 hours required in batch mode. The developed protocol can be seen as a general tool for rapid and facile generation of peptidomimetic compounds. We also showcase preliminary biological assessments for the prepared compounds.

## Graphical abstract

**Go with the flow**: We present a telescoped flow process combining interrupted Fischer and subsequent Joullié‐Ugi‐type modification in a safe, environmentally friendly, and cost‐effective mode. The developed protocol represents a general tool toward the diversity‐oriented generation of peptidomimetics. A preliminary biological assessment of the developed compounds revealed the potential for future optimization toward a targeted anti‐biofilm application.

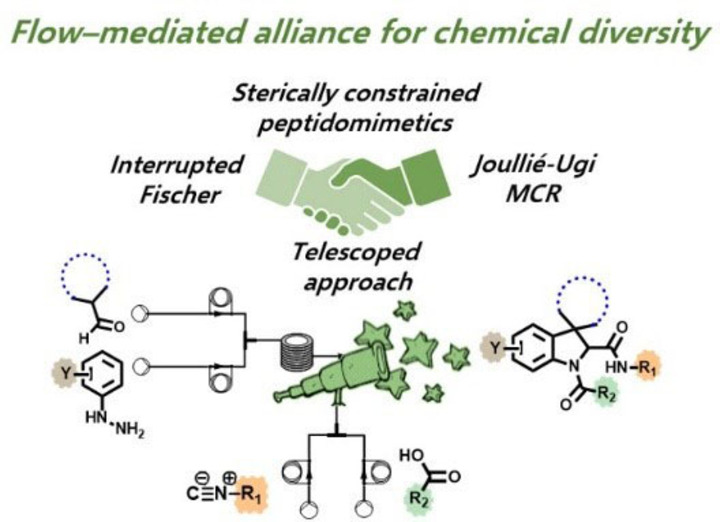

## Introduction

In parallel to the growing prominence of the peptidomimetic drug design approach in various fields of pharmaceutical chemistry and drug design in general, the synthetic methodologies applied to realize diverse peptidomimetic substructures have been increasingly improved.[^1^, ^2^, ^3^] Additionally, the past two decades saw the generation and evolution of diversity‐oriented synthesis (DOS) with the aim to generate architecturally more complex molecules, including peptidomimetic frameworks, for covering additional areas of chemical space.[^4^, ^5^, ^6^] Fully in line with the need for increased structural diversity is also the concept of increasing sp^3^‐character in active molecules, which guarantees the possibility of out‐of‐the plane substituents allowing fine‐tuning of receptor‐ligand complementarity. In this context, supplying peptidomimetic scaffolds with strategical spiro‐fused appendages is an increasingly growing approach in medicinal chemistry. So far, several approaches have been introduced for the synthesis of peptidomimetics; most of them, however, suffer from high number of steps and poor overall efficiency.[^7^, ^8^, ^9^, ^10^, ^11^]

In the field of straightforward ways to more complex and/or peptidomimetic drug‐like scaffolds, isocyanide‐based multicomponent reactions (MCRs), like the Ugi reaction and its variants, are widely established as useful tools for creating novel peptidomimetic structures, owing to the fact that diamide “peptoid” motifs are inherently created in the course of these reactions,[^12^, ^13^, ^14^, ^15^] in a diversity‐oriented fashion and with reduced numbers of synthetic steps.[^16^, ^17^, ^18^, ^19^, ^20^, ^21^, ^22^] Particularly attractive is the opportunity to construct, via a MCR, (spiro)cyclic constrained peptidomimetics which are expected to possess improved absorption properties due to reduced number of rotatable chemical bonds and to better mimic characteristic structural biases in peptides and proteins, i. e., ‐turns.[^23^, ^24^, ^25^]

**Figure 1:**
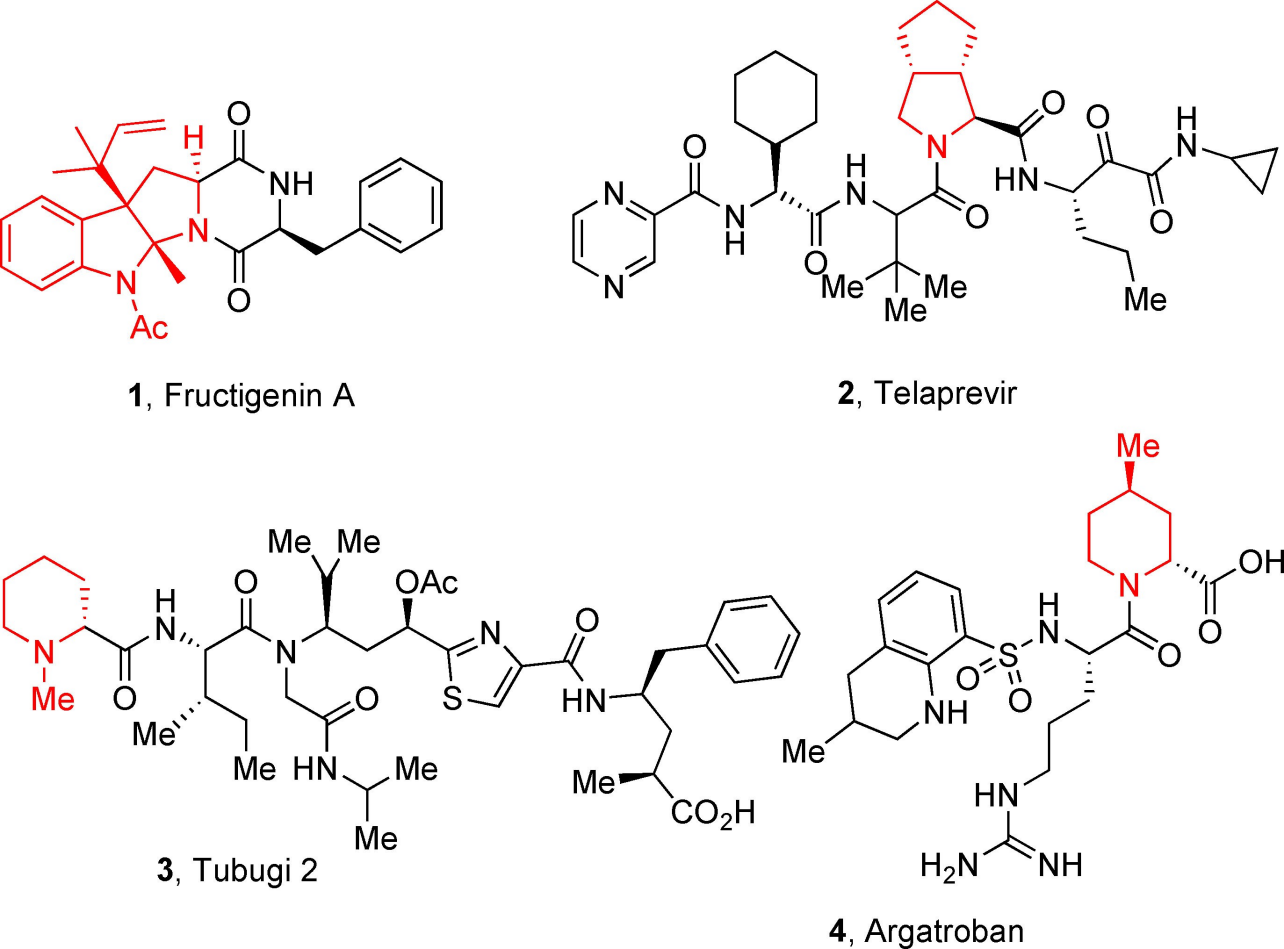
Examples of compounds (1–4) with medicinal applications obtained by the use of cyclic imines in Ugi‐type reactions.

Cyclic imines represent key substructures in naturally occurring and synthetic compounds possessing biological activities. Low stability of some cyclic imines and the reactivity of the C−N bond as electrophile make them excellent substrates for various transformations.[^26^, ^27^, ^28^, ^29^, ^30^] In this regard, several compounds and nature‐inspired molecular entities endowed with biological activities have been obtained via cyclic imines employing Ugi‐type reactions (Figure 1).^[31]^ Examples are the synthesis of fructigenin A (**1**), a pyrazino‐pyrroloindole alkaloid originally isolated from a Penicillum strain and displaying interesting antitumor properties.^[32]^ Another example is represented by the peptidomimetic anti‐HCV agent telaprevir (**2**) for which the original synthesis envisaged 24 steps;^[33]^ a 11‐step synthesis was then reported exploiting both an Ugi and a Passerini MCR as key steps.^[34]^

In the frame of peptidomimetic nature‐inspired scaffolds, tubulysins represent optimum starting points for the generation of new anticancer drugs, owing to their exceptionally high cytotoxic activity. Novel tubulysins analogues, like tubugi 2 (**3**), displaying a comparable potency to tubulysin A, have been recently developed utilizing three consecutive Ugi‐type MCRs.[^35^, ^36^]

Moreover, a synthesis for argatroban (**4**), a therapeutically employed anticoagulant agent, was proposed involving a MCR as crucial step.^[37]^

**Figure 2:**
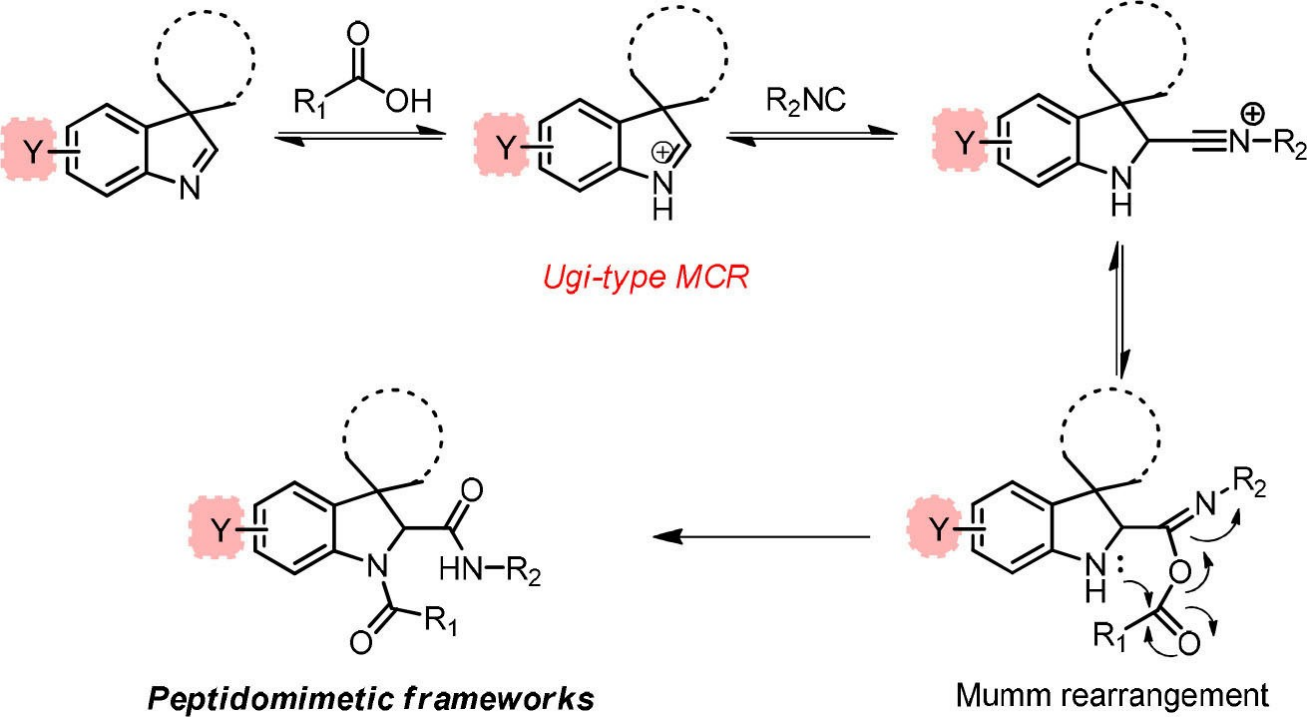
Joullié‐Ugi three‐component reaction based on (spiro)cyclic indolines.

The use of cyclic imines as substrates for MCRs involving isocyanides and carboxylic acids, namely in form of the Joullié‐Ugi reaction, allows introducing proline‐like constrained motifs in the structure of the resulting products.^[38]^ The established reactivity of indolenines in reactions that are typical of imines prompted us to consider them as substrates for the Joullié‐Ugi reaction, in order to generate novel peptidomimetics containing a spiroindoline backbone as arene‐fused proline mimics capable of not only displaying a turn‐like structural bias but also building additional hydrophobic or electronic interactions via the aromatic ring with a given biological target (Figure 2).^[25]^

In our quest toward the development of greener and more sustainable protocols for the synthesis of privileged scaffolds,^[39]^ we recently proposed a flow‐aided methodology for the generation of spiroindolenines and their reduced spiroindoline counterparts.^[40]^ Capitalizing on this newly developed protocol, we sought to investigate the usefulness of spiroindolenines as substrates for the generation of peptidomimetic frameworks through a Joullié‐Ugi MCR, using different amino acids and isocyanides for achieving structural variation. Based on insights gained in a preliminary batch investigation, batch‐to‐flow transition was realised first for the MCR step alone. Subsequently, a telescoped approach was established such as to directly use the flow‐borne spiroindolenine for the rapid generation of a library of peptidomimetics trough the Joullié‐Ugi reaction with overall increased space‐time yields, employing ethanol as green solvent for both steps.

To the best of our knowledge, this is one of the few protocols for MCR implementation in continuous flow in efficient telescoping mode, coupling the synthesis of the metastable spiroindolenine starting material under interrupted Fischer conditions with the subsequent Joullié‐Ugi MCR step. A thorough exploration of key parameters allowed the identification of the most efficient reagent mixing mode that was found to be most crucial, the optimum temperature and residence time. The newly developed method allowed straightforward reaction channelling toward the fast and complete formation of the MCR products, thus reducing the yield drop due to indolenine degradation during chromatographic purification or eventual storage, and limiting the resulting inevitable competing formation of side products. The generated compounds were preliminarily screened for their cytotoxicity and evaluated as anti‐biofilm agents against *Staphylococcus epidermidis*, thus also fostering further exploration and scaffold diversification through the developed methodology.

## Results and Discussion

### Basic screening of reaction conditions in batch

As a preliminary task, we tested the flexibility of the approach starting from spiroindolenine **5** selected as reference in form of a quick but inclusive exploration in batch (Table 1) and three *N*‐capped amino acids with diverse lateral chains and protecting groups, namely Cbz‐l‐Ala, Cbz‐l‐Phe and Bog‐l‐PhG (**7 a**–**c**). Apart from representing robust protecting groups in Joullié‐Ugi MCR conditions, Boc and Cbz functionalities would also provide a useful handle for accessing more complex spiroindoline‐based peptidomimetic structures. Moreover, in this preliminary batch inquiry we sought to investigate the reactivity in the presence of an isocyanide presenting: i) an electron‐rich aryl moiety, using *p*‐methoxyphenyl isonitrile, **6 a**, ii) a non‐activated aryl motif, using benzyl isocyanide **6 b**, and iii) a bulkier aliphatic moiety, using cyclohexyl isonitrile, **6 c**. We have also successfully applied the developed methodology to trityl isocyanide **6 d**, synthesized starting from trityl amine (see Supporting Information for details), and (*S*)‐3‐(acetylthio)‐2‐methylpropanoic acid **7 d**, a relevant amino acid in medicinal chemistry, since its free thiol counterpart is embedded in the structure of the drug Captopril. Moreover, the preparation of compound **17** also provides validation of the MCR methodology in the presence of labile chemical moieties such as the trityl group and the thioester functionality.

**Table 1:** Ugi‐Joullié products synthesized in batch mode.

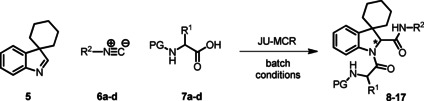				
Entry	Isonitrile	Amino acid	Products^[a]^	Yield [%]^[d]^
1	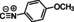	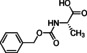	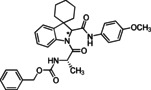	72
	**6 a**	*S‐* **7 a**	(*S*,S*)‐**8** and (*S*,R*)‐**8**	
2	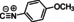	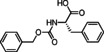	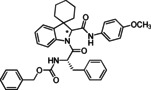	68
	**6 a**	*S‐* **7 b**	(*S*,S*)‐**9** and (*S*,R*)‐**9**	
3	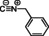	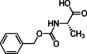	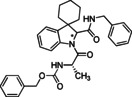	58
	**6 b**	*S‐* **7 a**	(*S*,S*)‐**10** and (*S*,R*)‐**10** ^[b]^	
4	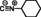	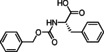	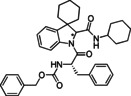	60
	**6 c**	*S‐* **7 b**	(*S*,S*)‐**11** and (*S*,R*)‐**11** ^[b]^	
5	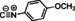	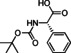	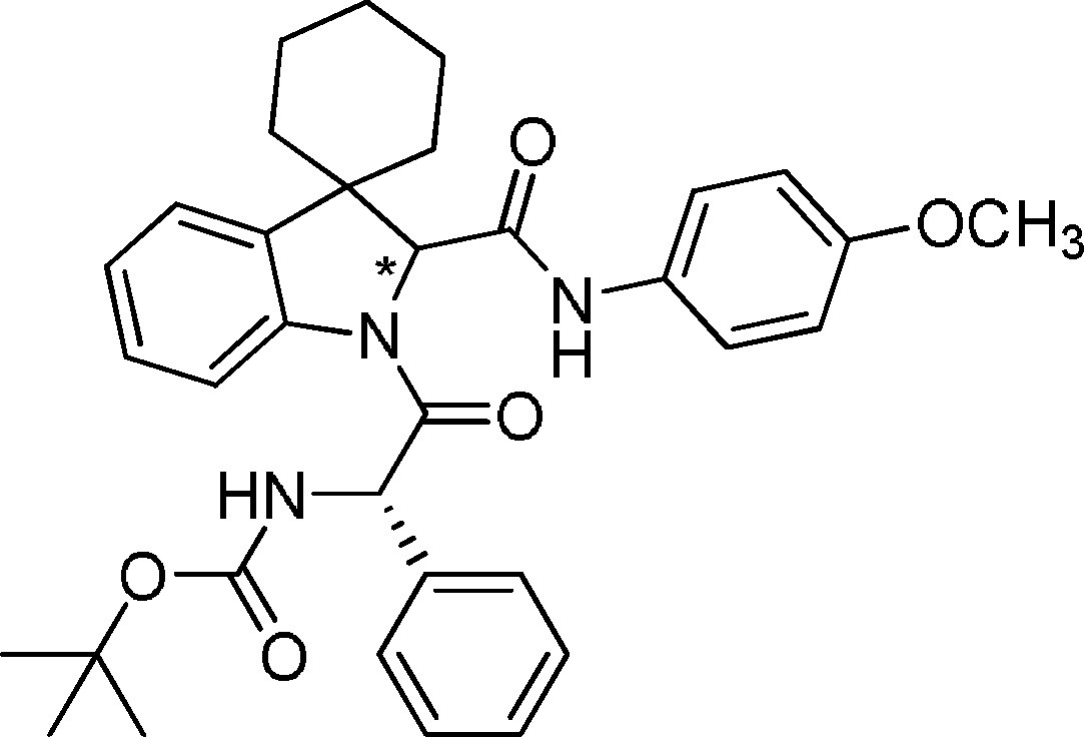	60
	**6 a**	*S‐* **7 c**	(*S*,S*)‐**12** and (*S*,R*)‐**12**	
6	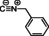	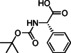	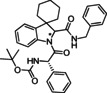	58
	**6 b**	*S‐* **7 c**	(*S*,S*)‐**13** and (*S*,R*)‐**13**	
7	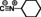	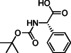	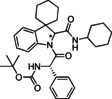	60
	**6 c**	*S‐* **7 c**	(*S*,S*)‐**14** and (*S*,R*)‐**14**	
8	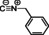	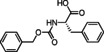	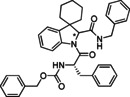	74
	**6 b**	*S‐* **7 b**	(*S,R/S*)‐**15**	
9	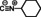	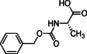	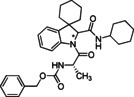	65
	**6 c**	*S‐* **7 a**	(*S,R/S*)‐**16**	
10	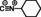	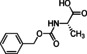	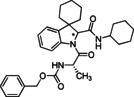	62
	**6 c**	*S‐* **7 a**	(*S,R/S*)‐**16** ^[c]^	
11	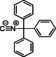	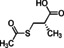	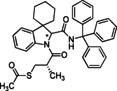	57
	**6 d**	*S‐* **7 d**	(*S,R/S*)‐**17**	

[a] Reactions conditions: Spiroindolenine **5** was dissolved in DCM (0.3 m) followed by the addition of the corresponding amino acid (1.0 eq) and isocyanide (1.0 eq). The reaction mixture was stirred for 30 h at 50 °C. [b] Reaction time: 24 hours; [c] Reactions conditions: Spiroindolenine **5** was dissolved in MeOH (0.3 m) followed by the addition of the corresponding amino acid (1.0 eq) and isocyanide (1.0 eq). The reaction mixture was stirred for 30 h at 50 °C. [d] Yield of the diastereomeric mixture after column chromatography as specified in the Experimental Section.

As spiroindolenine substrate **5**, conveniently prepared as reported before,^[40]^ was unstable to isolation, its use was thus studied without further purification after formation, by adding isocyanide and carboxylic acid in an equimolar ratio to a dichloromethane solution of **5**, which was obtained after the standard workup of the interrupted Fischer synthesis protocol. As a result, the expected peptidomimetic indolines **8**–**17** were obtained in good yields of 57 to 74 % in form of their diastereomeric mixtures, over two steps and after chromatographic purification (Table 1, entries 1–9).

Examination of the reactivity patterns obtained upon combining various indolenine **5**, isocyanides and *N*‐protected amino acids allowed to delineate some important features: i) the employment of a spiroindolenine as a sterically rather hindered cyclic imine in this reaction is possible; ii) the reaction displays a satisfactory tolerance to the character of both isocyanides and amino acids used in the reaction. Unquestionably, the latter represents a significant outcome with respect to the aim of developing conformationally restricted chemical probes for peptidergic biological targets. The quite extreme steric congestion around the imine carbon atom, created by the nearby quaternary centre and resulting eventually critical for the ‘access’ of the isocyanide component, appears to have only little influence on the yield of the reaction. Also, no peculiar reactivity trend could be observed upon variation of the nature of the isocyanide and/or the amino acid, as demonstrated by the limited variation of the registered isolated yields.

A diastereoselective outcome was not expected for our protocol, since experimental studies already established that the use of chiral isocyanides, amines, carboxylic acids, or carbonyl compounds as potential inducers of an asymmetric reaction pathway do not result in meaningful levels of diastereoselectivity.^[31]^ In this study, compounds were obtained in form of diastereoisomeric mixtures and separated through reverse‐phase HPLC as detailed in the Experimental Section. A deeper investigation would be needed, of course, considering the role a defined chiral centre within the starting indolenine system could play, with particular reference to the spirofused ring in 3‐position. Further studies on diastereoselectivity induction in our labs do currently aim at improving stereoselectivity and will be published in due course.

In our quest to shift toward a greener and more sustainable protocol for the synthesis, we attempted the replacement of dichloromethane with methanol, a quite commonly employed solvent for MCR protocols.^[41]^ Gratifyingly, the use of methanol provided comparable results in terms of yield and reaction times (Table 1, entry 10).

### Development of reactions conditions in segmented continuous flow mode

Based on these initial results, robust flow conditions were elaborated. Following the idea that flow chemistry applications do not necessarily need dedicated flow chemistry platforms, our MCR flow system was realized in a comparably cost‐effective fashion: a HPLC piston pump capable of sustaining reliably low flow rates was connected to a six‐port Rheodyne injector for meso‐scale preparative HPLCs, that was equipped with 1 mL volume sample loop made from PTFE tubing and an injection port for conventional disposable syringes. Connection to a self‐made tubular reactor of 15 mL, that was facultatively additionally connected to a commercial tubular reactor to reach 31 mL reactor volume, was realized *via* a simple T‐piece. The self‐made reactor was immersed in a conventional silicone oil bath for heating, while the commercial reactor was heated on a hot‐plate. To maintain control of the system, a 7 bar back‐pressure regulator was placed at the end of the line.

An equimolar solution of the spiroindolenine **5** and the appropriate amino acid and isocyanide was prepared in methanol (1 mL total volume) and injected into the sample loop. A mixing of the three components in the flow set‐up by means of a simple T‐piece was tested, but proved much less effective when otherwise applying winning parameter sets (results not shown). Injecting this solution into the flow system and pumping it at an overall flow rate of 0.2 mL/min through the reactor held at different temperatures caused the reaction mixture to produce the desired MCR product, that was collected as exiting stream in a flask for further purification.

Working with equimolar quantities of all three starting materials and keeping flow‐rate, reactor size and back‐pressure constant, three temperatures were initially screened (Table 2, entries 1–3). The analysis of the retrieved yields led to select 50 °C as ideal temperature, also considering the amount of overall consumed energy. Subsequent increase of residence time by lowering the flow rate led to drastic decline of isolated yield under otherwise unchanged conditions, suggesting that a prolonged residence time can cause significant undesired side reactions and/or decomposition of materials. In general, the implemented flow conditions, although causing a slight decrease in isolated yields of the diastereomeric mixtures, demonstrated the reaction compatibility with continuous flow conditions and, most importantly, proved that the reaction times could be reduced drastically applying the described set‐up.

**Table 2:** Optimisation of reaction conditions in flow for the preparation of peptidomimetics.

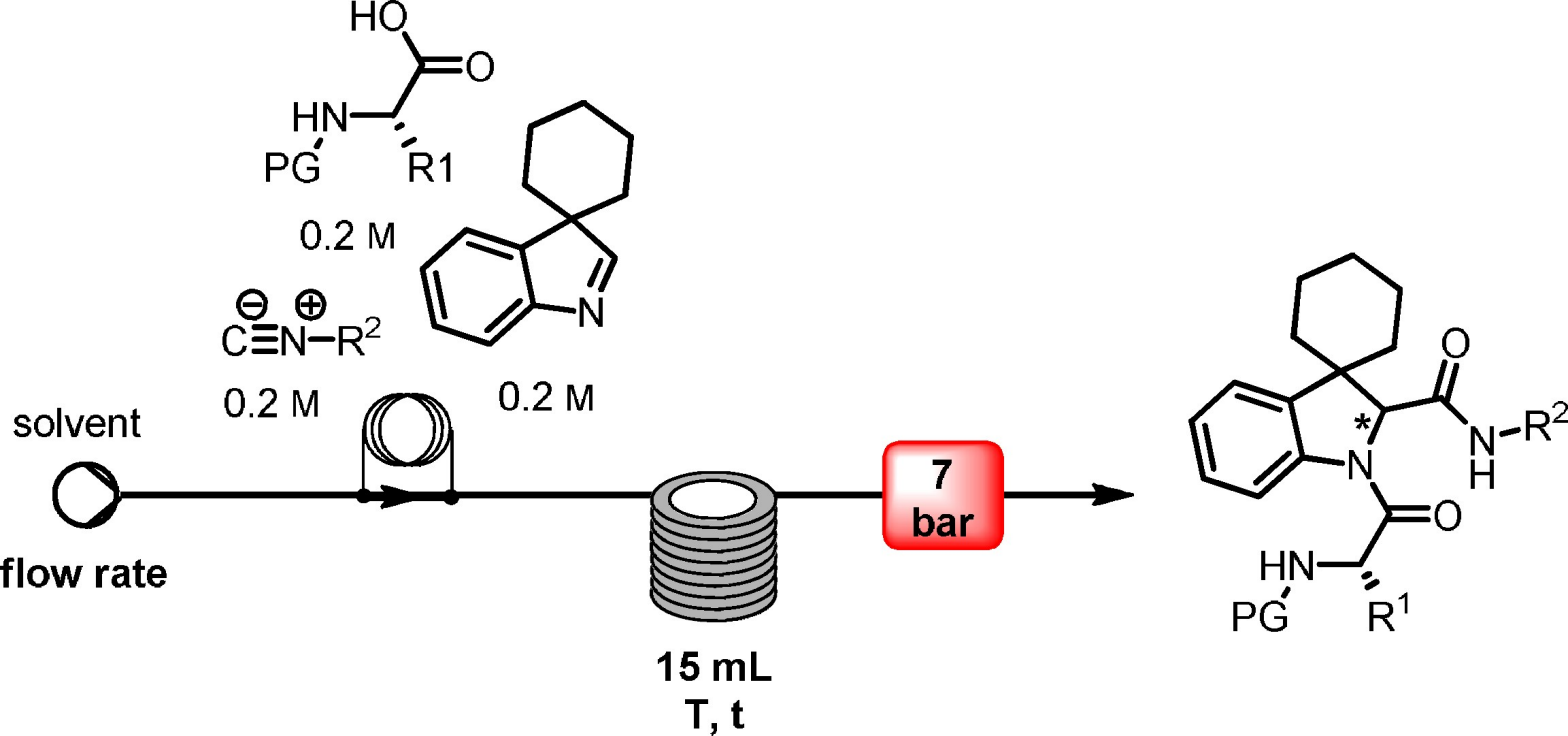								
Entry	Isonitrile	Amino acid	Product	Solvent	Flow rate [mL/min]	Residence time [min]	*T* [°C]	Yield [%]^[d]^
1	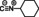	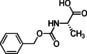	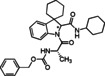	MeOH	0.2	75 ^[a]^	50	57
	**6 c**	*S‐* **7 a**	(*S,R/S*)‐**16**					
2	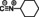	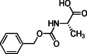	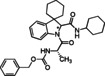	MeOH	0.2	75 ^[a]^	80	53
	**6 c**	*S‐* **7 a**	(*S,R/S*)‐**16**					
3	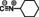	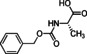	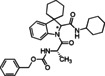	MeOH	0.2	75 ^[a]^	130	35
	**6 c**	*S‐* **7 a**	(*S,R/S*)‐**16**					
4	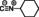	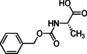	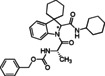	MeOH	0.1	155 ^[a]^	50	22
	**6 c**	*S‐* **7 a**	(*S,R/S*)‐**16**					
5	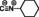	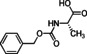	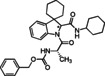	EtOH	0.2	155 ^[b]^	50	45
	**6 c**	*S‐* **7 a**	(*S,R/S*)‐**16**					
6	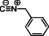	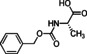	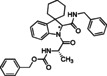	EtOH	0.2	75 ^[a]^	50	54
	**6 b**	*S‐* **7 a**	(*S*,S*)‐**10** (*S*,R*)‐**10**					
7	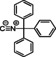	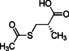	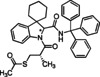	EtOH	0.2	75^[a]^	50	25^[d]^
	**6 d**	*S‐* **7d**	(*S*,S*)‐**17** (*S*,R*)‐**17**					

[a] Reactor volume: 15 mL; [b] Reactor volume: 30 mL; [c] Isolated yield for the diastereomeric mixture after column chromatographic purification; [d] The yield decreases in the protic solvent due to the competing Strecker‐type reaction path involving trityl isocyanide as a suitable cyanide source; the same reaction using DCM as solvent provided a 47 % yield.

Anticipating the possibility of an upstream modification to combine the MCR protocol with our previously developed flow‐based synthesis of spiroindolenine scaffolds, and to guarantee an improved adherence to green chemistry principles, we tested EtOH as solvent. EtOH fully recapitulated the findings employing methanol as solvent, prompting thus the idea of developing a telescoped approach for conveniently coupling interrupted Fischer spiroindolenine synthesis with the developed Ugi‐Joullié protocol.

### Generation of spiroindoline‐based peptidomimetics in telescoped segmented continuous flow

We were ultimately interested in using indolenines as starting point for more complex structures. To this end, the flow protocol was modified and extended into a telescoped segmented continuous flow protocol that hosts both the interrupted Fischer protocol presented earlier and the MCR step developed in this study.

To realise this, the self‐made flow system reported earlier for spiroindolenines was coupled with the flow set‐up for realising the MCR as reported above. In the winning configuration, the flow path previously reported^[40]^ for the spiroindolenine synthesis was extended by placing a column reactor filled with an excess of polymer‐supported tris(2‐aminoethyl)amine in line after a first 15 mL tubular reactor made from PTFA in order to remove both the HCl necessary for spiroindolenine formation and eventually unreacted aldehyde. Unreacted hydrazine and eventual by‐products in form of traces of non‐cyclised derivatives were supposed to not interfere, on the other hand, with the MCR protocol. Spiroindolenines were generated using this initial step with partial in‐line purification at an overall flow rate of 1 mL/min. Inevitable for‐ and backward tailing of the initial reaction plug resulted in an interval of approx. 20 min during which the generated spiroindolenine was found to exit this first part of the telescoped set‐up. Elution profiles, and thus exiting times varied expectedly as function of introduced structural variation. Monitoring of the exiting was possible without the use of a detector by simply paying attention to the colour of the exiting stream, a fact that proofed important for the next step. It is needless to say that a simple ‘by eye’ monitoring can be automated using appropriate in‐line detectors that would principally allow for subsequent pump control as needed for the subsequent steps (*vide infra)*.^[42]^

The stream exiting the column reactor was then combined by means of an effective mixing chip of 0.2 mL with a stream containing the appropriate amino acid and isocyanide in equimolar amounts, respectively, with these amounts representing, however, an inevitable varying slight excess of respect to theoretically maximum amount of spiroindoline produced in the first part of this simple flow set‐up. This stream containing acid and isonitrile, realised joining the single reagent streams *via* a simple T‐piece, was pumped into the mixing chip with an overall flow rate of 0.4 mL/min, achieved at with an overall flow rate of 0.8 mL/min, realised by combination of 0.2 mL/min flow rate for the pumps furnishing amino acid and isonitrile, respectively, and a change of the overall flow rate of the spiroindolenine part to 0.4 mL/min as soon as the spiroindolenine stream was about to reach the mixing chip, as indicated by the colouring of the solvent within the tubing.

The MCR reaction took place in a 16 mL tubular reactor held at 50 °C then, realising approx. 80 min of residence time for the MCR reaction maintaining an overall flow of 0.2 mL/min once the mixing was completed and the coloured stream finished exiting the mixing chip. Although a premixing of amino acid and isonitrile would have facilitated the overall set‐up, such a premixing was omitted for realising the most flexible telescoped flow system that was pressurised to 7 bar *via* a mechanical back pressure regulator. Using ethanol as solvent for the entire telescoped sequence, four different peptidomimetics were realised in excellent overall yields for the diastereomeric mixtures (Table 3). Most noteworthy, this was possible using only approx. 15 % of the combined amount of solvent that was necessary for the two batch processes and purifications of the first step done when applying the batch mode. Additionally, telescoped flow chemical processing allowed for a drastic reduction of the time needed for synthesizing the peptidomimetics, i.e., 100 min in flow mode instead of 24–48 h needed for the batch mode. The flow process was thus significantly more sustainable, greener and time‐efficient.

**Table 3:** Preparation of peptidomimetics in telescoped fashion.

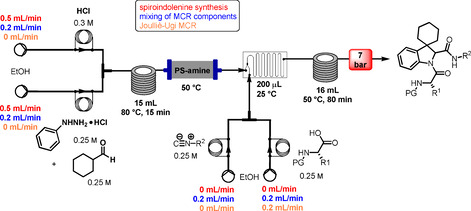					
Entry	Isonitrile	Amino acid	Product	Time [min]^[a]^	Yield [%]^[b]^
1	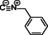	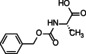	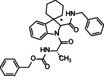	∼101	46
	**6 b**	*S‐* **7 a**	(*S*,S*)‐**10** and (*S*,R*)‐**10**		
	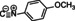	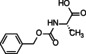	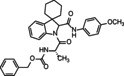	∼101	51
2	**6 a**	*S‐* **7 a**	(*S*,S*)‐**8** and (*S*,R*)‐**8**		
3	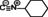	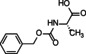	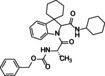	∼101	56
	**6 c**	*S‐* **7 a**	(*S,R/S*)‐**16**		
4	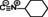	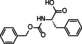	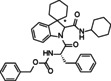	∼101	47
	**6 c**	*S‐* **7 b**	(*S*,S*)‐**11** and (*S*,R*)‐**11**		

[a] See main discussion for details about change in flow rate and related changes to exiting times, etc.; [b] Isolated yield after column chromatography.

### Biological studies

Bacterial biofilms pose a major threat to public health, as they account for at least two third of all infections in humans. Bacteria within biofilms are embedded in a self‐produced extracellular matrix, a scaffold with vital properties of retaining and protecting the biofilm.^[43]^ The intercellular cell‐cell signaling engaged by bacteria, generally referred to asquorum sensing, allows them to monitor their environment and quickly respond to diverse fluctuations and stimuli.[^44^, ^45^, ^46^] The major challenge associated with treatment of biofilms is their increased resilience and tolerance to conventional antimicrobial drugs.^[47]^

Despite the steady developments in surgical practices and biomaterial design, biomaterial‐associated infections account for a large portion of all nosocomial infections and pose an enormous global economic burden, as they can compromise or even cause the failure of the medical implant.[^48^, ^49^, ^50^]

As a part of the innate immune system, antimicrobial peptides have drawn attention within the last decades, owing to their capability of eradicating biofilms at sub‐minimum inhibitory concentration (MIC) levels. However, these peptides possess several disadvantages, such as susceptibility to proteolytic degradation, pH and/or salinity‐dependent activity and reduced bioavailability due to binding to serum proteins. Moreover, naturally derived peptides able to eradicate biofilm are often obtained by costly, low‐yielding, and time‐consuming extraction processes. Hence, the design of peptidomimetics able to overcome these drawbacks is still a pressing need. Our fast and reliable synthetic protocol allows quick and diverse structural modifications of peptidomimetic derivatives, thus paving the way to a deeper understanding of the relationship between structure and potential antibiofilm activity.

First, in order to establish if compounds **8**–**16** possessed the ability to arrest the growth of different pathogenic bacteria, minimal inhibitory concentrations (MIC) were determined by broth microdilution assay. The assay was conducted against two Gram positive strains, *Staphylococcus aureus* ATCC 43300 (MRSA) and *S. epidermidis* ATCC 35984 (biofilm producer), and two Gram negative strains, *Pseudomonas aeruginosa* ATCC 27853 and *Klebsiella pneumoniae* ATCC BAA‐1705 (carbapenem resistant). The antibiotics oxacillin, vancomycin, tobramycin, and imipenem were used as positive control against *S. epidermidis*, *S. aureus*, *P. aeruginosa*, and *K. pneumoniae*, respectively, according to European Committee on Antimicrobial Susceptibility Testing (EUCAST version 9.0, 2019). As reported in Table S1 in the Supporting Information, however, none of the compounds tested was able to affect the growth of the microorganisms studied. To exclude the possibility that the observed inefficacy of the novel peptidomimetics to affect cell growth was due to complex bacteria cell wall structure, the compounds were tested against yeast *Candida albicans*. Also, as reported in Table S1, no appreciable biological activity was measured.

*Staphylococcus epidermidis* is a normal inhabitant of skin as a member of human microbiota.^[51]^ Nevertheless, it can be responsible of severe infections associated to medical devices based on its important role in biofilm formation on their surfaces, including implants and urinary catheters.^[52]^

**Figure 3:**
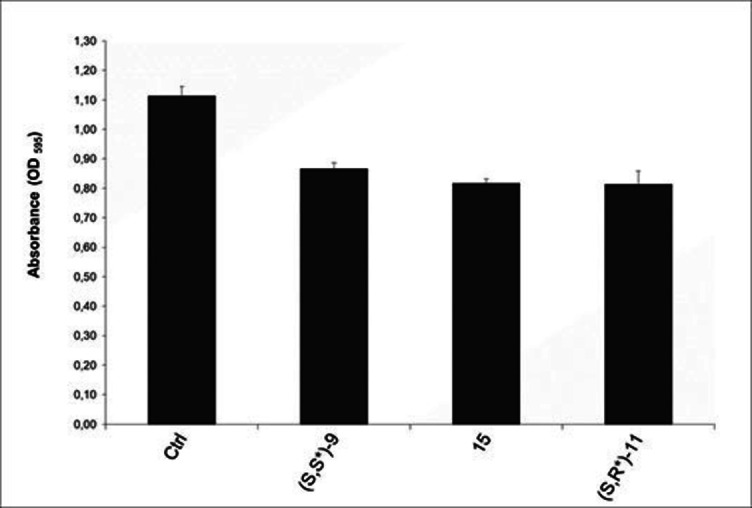
Effect of compounds (S,S*)‐ **9**, (*S,R/S*)‐**15** and (S,R,*)‐ **11** on *S. epidermidis* biofilm reduction. Values represents the means±standards deviations from three independent experiments. Control=DMSO treated cells (vehicle).

We thus investigated the suitability of our developed peptidomimetics for *S. epidermidis* biofilm inhibition. Applying the crystal violet assay, we observed a moderate activity of (S,S*)‐**9**, (S,R*)‐**11** and **15**. Compounds (*S,R/S*)‐**15** and (S,R*)‐**11** were both able to slightly reduce biofilm formation by 25 %, while (S,S*)‐**9** by 20 % when tested at 100 μm concentration (Figure 3). The latter results are encouraging since infections due to biofilm are difficult to treat and foster further investigation and optimization for this class of compounds.

**Figure 4:**
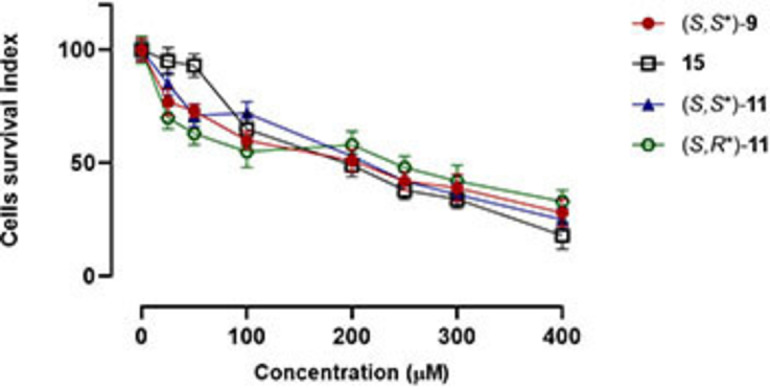
Cell survival index, evaluated by the MTT assay and live/dead cell ratio analysis. Human keratinocytes (HaCaT cells) were incubated for 48 with a range of concentrations (25→400 μM) of (S,S*)‐**9**, (S,S*)‐**11**, (S,R*)‐**11** and (*S,R/S*)‐**15**. Results are expressed in lines graph as the percentage of cell survival index to untreated control cultures and are reported as mean±SEM (*n*=4) of three independent experiments.

To further characterize the biological profile of compounds (S,S*)‐**9**, (S,S*)‐**11**, (S,R*)‐**11** and (*S,R/S*)‐**15**, a preliminary bioscreen *in vitro* was performed to establish the effect on human HaCaT cells (Figure 4), immortalized keratinocytes extensively used to study the epidermal homeostasis and its pathophysiology, including skin bacterial infection and biofilm deposition.[^53^, ^54^] Cell survival, reported as % of control, was evaluated by both the MTT assay and automatic cell count. The concentration‐effect curves reveal a good biocompatibility of all the molecules under investigation whereas significant cytotoxic effects are detectable only at very high *in vitro* concentrations. Indeed, IC_50_ values >200 μM were observed, thus indicating a potentially favourable safety margin.

## Conclusion

The synthetic protocol presented herein is of general utility for the generation of peptidomimetic frameworks trough Joullié‐Ugi MCR in a safe, environmentally friendly, and cost‐effective flow chemistry mode. Using a telescoped sequence it was possible to generate *in situ* the spiroindolenine motif that was used as imine component in the flow Joullié‐Ugi MCR saving 95 % of the time need for traditional batch syntheses. This protocol could additionally be amenable to a facilitated up‐scaling. Basic biological tests revealed a moderate activity against *S. epidermidis* and a low cytotoxicity, thus opening a scene for eventually optimizing the structure toward a targeted anti‐biofilm application.

## Experimental Section

### Chemistry

Unless otherwise specified, materials were purchased from commercial suppliers and used without further purification. TLC analysis was conducted using aluminum foil supported thin‐layer silica gel chromatography plates (F254 indicator). Column chromatography was performed using 230–400 mesh, 60 Å pore diameter silica gel. For ^1^H NMR and ^13^C NMR measurements an accurately weighed amount of analyte (about 5.0–10.0 mg) was dissolved in 600 μL of deuterated chloroform (CDCl_3_) or dimethyl sulfoxide (DMSO‐*d*_6_). The mixture was transferred into a 5 mm NMR tube and the spectra were acquired on a Bruker Advance 400 MHz spectrometer by using the residual signal of the deuterated solvent as internal standard. Splitting patterns are described as singlet (s), doublet (d), triplet (t), quartet (q) and broad (br); the values of chemical shifts (δ) are given in ppm and coupling constants (*J*) in Hertz (Hz). NMR data were processed with MestreNova (Version 8.1.1, Mestrelab Research). ESI‐MS spectra analysis was carried out on a mass spectrometer LTQ‐XL. HPLC was performed with a Waters Model 510 pump equipped with Waters Rheodyne injector and a differential refractometer, model 401. Luna 5 μm PFP (2) 100 A HPLC Column 250×10 mm was employed. The purity of compounds was estimated to be greater than 95 % by HPLC analysis. Optical rotation values were measured at room temperature operating at λ=589 nm, corresponding to the sodium D line, and were determined in a Jasco P‐200 (Jasco Europe s.r.l., Cremella, Italy) polarimeter using a microcell (1 mg sensitivity) containing the compound dissolved in 1 mL of chloroform.

#### Continuous flow synthesis of spiroindolenine 5

Chemical transformations in flow were realized using a self‐made flow reactor: two Waters P510 HPLC pumps were connected each to a Rheodyne 9010 injection valves, each equipped with a 1 mL sample loop (SL) made from PTFE tubing and an injection port for disposable syringes. The injection valves were further connected *via* a T‐piece to a coiled PTFE tubing of 15 mL volume. The coil was immersed in a silicone oil bath for controlling reaction temperatures. The tubular reactor finished into a back pressure regulator (BPR), which was either spring mechanism‐based (6.7 bar) or simply a PEEK capillary of defined length (1.7 bar). Product containing exiting streams were collected directly in a flask. An aliquot of the collected phase was used for analysis by GC‐MS. Reagents were prepared for loading into the sample loops according to one of the following this method: 0.25 mmol aldehyde+0.25 mmol PhNHNH_2_ ⋅ HCl in 1 mL of EtOH, heated to 50 °C for 5 min prior to loading into the SL; SL B: (equiv.–1) HCl in 1 mL of EtOH.

**Spiro[cyclohexane‐1,3′‐indole] 5**. A mixture of phenylhydrazine salt (36.5 mg, 0.25 mmol) and cyclohexanecarboxaldehyde (30 μL, 0.25 mmol) was treated with HCl (150 μL, 0.3 mmol). Next, the reaction mixture was gradually poured into saturated aqueous NaHCO_3_ (100 mL) and extracted with EtOAc (5×2 mL). Combined organic extracts were washed with brine, dried with Na_2_SO_4_, and used without purification. Yield: 58 %; yellow solid; *R_f_* =0.6 (H : E=7 : 3). GC‐MS (EI, 70 eV): *m*/*z* (%)=187 [M]^+^ *t_R_* =14.94 min.

#### Synthesis of multicomponent products in batch (General procedure A)

Spiroindolenine **5** (0.54 mmol) was dissolved in DCM (0.3 M) followed by the addition of the corresponding amino acid (1.0 eq) and isocyanide (1.0 eq). The reaction mixture was stirred for 24–30 h at 50 °C. Then, DCM was evaporated under vacuum to achieve the crude residue that was subjected to column chromatography (SiO_2_, corresponding eluent) and to HPLC to afford a diastereomeric mixture.

#### Synthesis of multicomponent products in flow (General procedure B)

Chemical transformations in flow were realized using a self‐made flow reactor: a Waters P510 HPLC pump was connected to a Rheodyne 9010 injection valve, equipped with a 1 mL sample loop made from PTFE tubing and an injection port for disposable syringes. The injection valve was further connected to a coiled PTFE tubing of 15 mL volume. The coil was immersed in a silicone oil bath for controlling reaction temperatures. The tubular reactor finished into a mechanical back pressure regulator (BPR) of approx. 7 bar). Product containing exiting streams were collected directly in a flask.

A solution of spiroindolenine **5** (49.5 mg, 0.25 mmol), the suitable amino acid (0.25 mmol) and isocyanide (0.25 mmol) was prepared in MeOH (1 mL total solution volume). The flow reactor was heated to 80 °C MeOH was used as solvent at a total flow rate of 0.2 mL/min to move the reaction mixture through the coil reactor for a residence time of 75 min.

#### Synthesis of multicomponent products in telescoped continuous flow (General procedure C)

The initial spiroindolenine formation step was performed analogously to the above reported conditions. Two HPLC pumps were connected to Rheodyne 9010 injection valves, each equipped with a 1 mL sample loop made from PTFE tubing and an injection port for disposable syringes. One sample loop was loaded with phenylhydrazine hydrochloride salt and cyclohexylcarbaldehyde (each 0.25 M in EtOH) and the other one with ethanolic HCl (0.3 M in EtOH). These two lines were joined by means of a T‐piece that connected the injection part to a coiled PTFE tubing of 15 mL volume. The coil was immersed in a silicone oil bath for controlling reaction temperatures. This tubular reactor was followed by an Omnifit column reactor filled with an excess (in mmol) of polymer‐supported tris(2‐aminoethyl)amine. The exit of the column reactor was connected to one inlet of an effecting mixing chip (Sigma Aldrich glass microreactor LTF MR Lab MX) of 0.2 mL.

Two additional HPLC pumps were connected to two other six‐port injection valves, each equipped with a 1 mL sample loop made from PTFE tubing and an injection port for disposable syringes, allowing for separate streams containing the appropriate amino acid (0.25 m ethanolic solution in sample loop) and isocyanide (0.25 m ethanolic solution in sample loop). The two streams were joined *via* a simple T‐piece before being led *via* the second inlet port into the mixing chip. Mixing was achieved with an overall flow rate of 0.8 mL/min, realised by combination of 0.2 mL/min flow rate for the pumps furnishing acid and isonitrile, respectively, and a change of the overall flow rate of the spiroindolenine part to 0.4 mL/min as soon as the spiroindolenine stream was about to reach the mixing chip, as indicated by the colouring of the tube content.

As soon as the fading colour of the solvent in the tubing indicated the completion of mixing, the MCR reaction was allowed to take place in a 16 mL tubular reactor held at 50 °C, by ultimately lowering the flow rate to 0.2 mL/min by switching off three of the four pumps.

The overall telescoped flow system was pressurised to 7 bar *via* a mechanical BPR. Product containing exiting streams were collected directly into a flask.

#### Compound characterization

**Benzyl ((2*S*)‐1‐(2′‐((4‐methoxyphenyl)carbamoyl)spiro[cyclohexane‐1,3′‐indolin]‐1′‐yl)‐1‐oxopropan‐2‐yl)carbamate (8)**. According to general procedure A, a mixture of spiro[cyclohexane‐1,3′‐indole] **5** (100 mg, 0.54 mmol), Z‐L‐alanine **7 a** (120 mg, 0.54 mmol) and 4‐methoxyphenyl isocyanide **6 a** (71 mg, 0.54 mmol) was dissolved in DCM (2 mL) in a sealed tube at 50 °C for 30 hours. The crude product was purified by chromatography on silica gel with hexane/ EtOAc (7 : 3) to yield the single diastereoisomers. The products were subjected to HPLC with MeOH/H_2_O (85 : 15) as the eluent (flow rate 3.00 mL/min) for purity determination. Total yield: 72 % (*dr* 1 : 1).

(*S*,S*)‐**8**: yellowish solid; R_*f*_=0.45 (H : E=7 : 3). ^1^H NMR (400 MHz, DMSO‐*d*_6_) δ 10.49 (s, 1H), 7.99 (d, 1H), 7.87 (d, 1H), 7.47 (d, 2H), 7.38–7.31 (m, 5H), 7.22 (d, 1H), 7.16 (t, 1H), 7.03 (t, 1H), 6.89 (d, 2H), 5.31 (s, 1H), 5.06 (d, 1H), 5.00 (d, 1H), 4.56 (t, 1H), 3.72 (s, 3H), 1.93–1.86 (m, 2H), 1.72–1.60 (m, 5H), 1.24 (s, 3H), 1.10 (d, *J*=6.8 Hz, 3H). ^13^C NMR (101 MHz, DMSO) δ 172.4, 167.2, 156.4, 156.2, 142.9, 141.1, 137.4, 131.6, 128.8 (2), 128.2, 128.1 (2), 127.4, 124.0, 122.5, 121.7 (2), 116.8, 114.5 (2), 70.2, 65.9, 55.6 (2), 48.8, 31.1, 30.0, 25.5, 23.2, 22.3, 16.8. ESI‐MS m/z: [M+H]^+^ 542. *tR* HPLC=11.19 min. [α]^20^_D_=+7.76 (*c* 0.3, CHCl_3_).

(*S*,R*)‐**8**: yellowish solid; R_*f*_=0.35 (H : E=7 : 3). ^1^H NMR (400 MHz, DMSO‐*d*_6_) δ 10.33 (s, 1H), 8.01 (d, *J*=8.0 Hz, 1H), 7.59 (d, *J*=7.4 Hz, 1H), 7.47–7.42 (m, 2H), 7.34–7.30 (m, 4H), 7.23–7.18 (m, 2H), 7.04 (d, *J*=7.1 Hz, 1H), 6.89 (t, *J*=9.0 Hz, 2H), 4.99–4.93 (m, 3H), 4.55–4.48 (m, 1H), 3.72 (s, 3H), 1.94–1.81 (m, 3H), 1.73–1.69 (m, 2H), 1.65–1.62 (m, 2H), 1.35 (d, *J*=6.5 Hz, 3H), 1.24 (s, 3H). ^13^C NMR (101 MHz, CDCl_3_) δ 171.4, 166.4, 156.9, 155.4, 140.4, 139.7, 136.2, 129.5, 128.5 (3), 128.1 (3), 125.6, 123.1 (2), 122.9, 117.9, 114.0 (2), 71.2, 66.9, 55.4, 49.3, 39.5, 30.3, 29.7, 25.4, 22.7 (2), 18.6. ESI‐MS *m/z*: [M+H]^+^ 542; *tR* HPLC=11.79 min. [α]^20^_D_=−22.13 (*c* 0.3, CHCl_3_).

**Benzyl((2*S*)‐1‐(2′‐((4‐methoxyphenyl)carbamoyl)spiro[cyclohexane‐1,3′‐indolin]‐1′‐yl)‐1‐oxo‐3‐phenylpropan‐2‐yl)carbamate (9)**. According to general procedure A, a mixture of spiro[cyclohexane‐1,3′‐indole] **5** (100 mg, 0.54 mmol), Z‐L‐Phenylalanine **7 b** (161 mg, 0.54 mmol) and 4‐methoxyphenyl isocyanide **6 a** (71 mg, 0.54 mmol) was dissolved in DCM (2 mL) in a sealed tube at 50 °C for 30 hours. The crude product was purified by chromatography on silica gel with hexane/EtOAc (7 : 3) to yield the single diastereoisomers. The products were subjected to HPLC with MeOH/H_2_O (85 : 15) as the eluent (flow rate 3.00 mL/min) for purity determination. Total yield: 68 % (*dr* 1 : 1).

(*S*,S*)‐**9**: yellowish solid; R_*f*_=0.62 (H : E=7 : 3). ^1^H NMR (400 MHz, DMSO‐*d*_6_) δ 10.64 (s, 1H), 8.03 (t, *J*=7.6 Hz, 2H), 7.55 (d, *J*=7.6 Hz, 2H), 7.35–7.25 (m, 6H), 7.20–7.06 (m, 7H), 6.92 (d, *J*=7.9 Hz, 2H), 5.49 (s, 1H), 4.97 (s, 2H), 4.61 (s, 1H), 3.72 (s, 3H), 2.92–2.83 (m, 2H), 1.98–1.90 (m, 2H), 1.79–1.62 (m, 6H), 1.27–1.21 (m, 2H). ^13^C NMR (101 MHz, CDCl_3_) δ 169.9, 166.7, 156.7, 156.6, 140.9, 140.5, 136.0, 135.8, 130.2, 129.3 (2), 129.1, 128.5 (2), 128.2, 128.0 (2), 126.8, 125.2, 125.0, 124.9, 122.6, 121.6 (2), 117.7, 114.1 (2), 71.0, 67.3, 55.5, 54.8, 49.2, 39.5, 38.3, 30.3, 25.3, 23.2, 22.7. ESI‐MS *m/z*: [M+H]^+^ 618; *tR* HPLC=22.22 min. [α]^20^_D_=+23.93 (*c* 0.3, CHCl_3_).

(*S*,R*)‐**9**: yellowish solid; R_*f*_=0.52 (H : E=7 : 3). ^1^H NMR (400 MHz, DMSO‐*d*_6_) δ 10.30 (s, 1H), 8.08 (d, *J*=7.8 Hz, 1H), 7.58 (d, *J*=8.5 Hz, 1H), 7.51 (d, *J*=8.5 Hz, 1H), 7.42 (d, *J*=8.8 Hz, 2H), 7.29 (d, *J*=9.3 Hz, 6H), 7.20–7.17 (m, 3H), 7.07–6.98 (m, 2H), 6.86 (d, *J*=8.7 Hz, 3H), 4.90–4.86 (m, 1H), 4.84 (s, 2H), 4.75 (d, *J*=12.6 Hz, 1H), 3.70 (s, 3H), 3.03–2.95 (m, 2H), 1.89–1.79 (m, 2H), 1.71–1.61 (m, 6H), 1.37–1.27 (m, 2H). ^13^C NMR (101 MHz, CDCl_3_) δ 170.4, 166.3, 156.8, 155.2, 140.2, 140.0, 136.1, 135.5, 129.3 (2), 128.9 (2), 128.4 (4), 128.0 (3), 127.2 (2), 125.5, 122.8 (2), 117.7, 113.9 (2), 71.8, 66.9, 55.4, 54.9, 48.9, 39.7, 39.6, 30.6, 25.3, 23.1, 22.7. ESI‐MS *m/z*: [M+H]^+^ 618; *tR* HPLC=23.54 min. [α]^20^_D_=+2.63 (*c* 0.3, CHCl_3_).

**Benzyl((2*S*)‐1‐(2′‐(benzylcarbamoyl)spiro[cyclohexane‐1,3′‐indolin]‐1′‐yl)‐1‐oxopropan‐2‐yl)carbamate (10)**. According to general procedure A, a mixture of spiro[cyclohexane‐1,3′‐indole] **5** (100 mg, 0.54 mmol), Z‐L‐Alanine **7 a** (161 mg, 0.54 mmol) and benzyl isocyanide **6 b** (65 μL, 0.54 mmol) was dissolved in DCM (2 mL) in a sealed tube at 50 °C for 24 hours. The crude product was purified by chromatography on silica gel with hexane/EtOAc (7 : 3) and by HPLC with MeOH/H_2_O (82 : 18) as the eluent (flow rate 3.00 mL/min) to yield single diastereomers. Total yield: 58 % (*dr* 1 : 1)

(*S*,S*)‐**10**: yellowish solid; R_*f*_=0.62 (H : E=7 : 3). ^1^H NMR (400 MHz, DMSO‐*d*_6_) δ 8.98 (s, 1H), 7.99 (d, *J*=7.8 Hz, 1H), 7.56 (d, *J*=7.3 Hz, 1H), 7.42–7.38 (m, 3H), 7.31–7.23 (m, 7H), 7.19–7.15 (m, 2H), 7.03 (d, *J*=7.1 Hz, 1H), 5.08 (q, *J*=12.6 Hz, 2H), 4.89 (s, 1H), 4.57–4.48 (m, 1H), 4.38–4.31 (m, 1H), 4.25–4.18 (m, 1H), 1.75 (d, *J*=13.4 Hz, 1H), 1.64–1.52 (m, 6H), 1.34–1.22 (m, 6H). ^13^C NMR (101 MHz, DMSO) δ 171.7, 167.7, 155.8, 142.8, 141.1, 139.0, 137.5, 128.8 (2), 128.7 (2), 128.4 (2), 128.2, 128.1 (3), 127.4, 124.0, 122.1, 116.7, 68.3, 65.8, 49.2, 48.5, 43.1, 29.7, 25.6, 23.2, 22.2, 21.8, 18.7. ESI‐MS *m/z*: [M+H]^+^ 526; *tR* HPLC=18.79 min. [α]^20^_D_=−34.50 (*c* 0.3, CHCl_3_).

(*S*,R*)‐**10**: yellowish solid; R_*f*_=0.62 (H : E=7 : 3). ^1^H NMR (400 MHz, DMSO‐*d*_6_) δ 9.08 (s, 1H), 7.95 (d, *J*=7.9 Hz, 1H), 7.81 (d, *J*=6.0 Hz, 1H), 7.35–7.31 (m, 6H), 7.30–7.27 (m, 3H), 7.22–7.10 (m, 3H), 7.01 (t, *J*=7.3 Hz, 1H), 5.20 (s, 1H), 5.06–4.95 (m, 2H), 4.42–4.36 (m, 1H), 4.26 (d, *J*=5.4 Hz, 2H), 1.80 (dd, *J*=17.8, 10.0 Hz, 2H), 1.69–1.55 (m, 6H), 1.27–1.16 (m, 2H), 1.00 (d, *J*=6.8 Hz, 3H). ^13^C NMR (101 MHz, DMSO) δ 172.2, 168.5, 156.4, 142.7, 141.3, 139.1, 137.3, 128.8 (2), 128.7 (2), 128.6 (2), 128.3, 128.1 (2), 127.5, 127.3, 124.0, 122.3, 116.9, 69.7, 65.9, 48.6, 48.4, 43.0, 29.9, 25.5, 23.2, 22.3, 16.9 (2). ESI‐MS *m/z*: [M+H]^+^ 526; *tR* HPLC=20.79 min. [α]^20^_D_=+9.63 (*c* 0.3, CHCl_3_).

**Benzyl((2*S*)‐1‐(2′‐(cyclohexylcarbamoyl)spiro[cyclohexane‐1,3′‐indolin]‐1′‐yl)‐1‐oxo‐3‐phenylpropan‐2‐yl)carbamate (11)**. According to general procedure A, a mixture of spiro[cyclohexane‐1,3′‐indole] **5** (100 mg, 0.54 mmol), Z‐L‐phenylalanine **7 b** (161 mg, 0.54 mmol) and cyclohexyl isocyanide **6 c** (66 μL, 0.54 mmol) was dissolved in DCM (2 mL) in a sealed tube at 50 °C for 24 hours. The crude product was purified by chromatography on silica gel with hexane/ EtOAc (8 : 2) and by HPLC with MeOH/H_2_O (82 : 18) as the eluent (flow rate 3.00 mL/min) to yield single diastereomers. Total yield: 60 % (*dr* 1 : 1).

(*S*,S*)‐**11**: yellowish solid; R_*f*_=0.51 (H : E=7 : 3). ^1^H NMR (400 MHz, DMSO‐*d*_6_) δ 8.45 (d, *J*=6.6 Hz, 1H), 7.99 (d, *J*=7.8 Hz, 1H), 7.89 (d, *J*=7.4 Hz, 1H), 7.34–7.24 (m, 10H), 7.21–7.19 (m, 1H), 7.15 (d, *J*=7.5 Hz, 1H), 7.02 (t, *J*=7.3 Hz, 1H), 5.27 (s, 1H), 4.96 (s, 2H), 4.69–4.61 (m, 1H), 3.46 (s, 1H), 2.90 (d, 1H), 2.76 (d, *J*=12.9 Hz, 1H), 1.87–1.83 (m, 1H), 1.75–1.59 (m, 9H), 1.55–1.49 (m, 3H), 1.25–1.10 (m, 7H).

^13^C NMR (101 MHz, DMSO) δ 171.1, 167.6, 156.7, 142.8, 141.5, 137.9, 137.2, 129.7 (2), 128.7 (2), 128.3 (2), 128.2, 128.0 (2), 127.3, 126.8, 124.0, 122.4, 116.9, 69.6, 65.9, 54.1, 48.6 (2), 36.4, 32.3, 31.9, 29.7, 25.6 (3), 24.9, 24.7, 23.3, 22.3. ESI‐MS *m/z*: [M+H]^+^ 542; *tR* HPLC=18.87 min. [α]^20^_D_=+19.78 (*c* 0.28, CHCl_3_).

(*S*,R*)‐**11**: yellowish solid; R_*f*_=0.43 (H : E=7 : 3). ^1^H NMR (400 MHz, DMSO‐*d*_6_) δ 8.34 (d, *J*=7.1 Hz, 1H), 8.05 (d, *J*=7.8 Hz, 1H), 7.37–7.24 (m, 11H), 7.15–7.10 (m, 2H), 6.99 (d, *J*=6.9 Hz, 1H), 5.00 (s, 2H), 4.81–4.73 (m, 1H), 4.64 (s, 1H), 3.48 (s, 1H), 2.97 (d, *J*=6.9 Hz, 2H), 1.83–1.76 (m, 2H), 1.73–1.58 (m, 10H), 1.27–1.12 (m, 7H), 1.02 (s, 1H). ^13^C NMR (101 MHz, DMSO) δ 169.9, 166.5, 155.6, 142.7, 141.2, 137.4, 136.9, 129.8 (2), 128.7 (2), 128.6 (2), 128.2, 127.9 (2), 127.4, 126.9, 124.1, 122.3, 116.7, 69.4, 65.7, 54.5, 48.2, 48.1, 32.6 (2), 32.1, 29.8, 25.7 (3), 25.1, 24.9, 23.1, 22.4. ESI‐MS *m/z*: [M+H]^+^ 542; *tR* HPLC=19.75 min. [α]^20^_D_=−2.33 (*c* 0.3, CHCl_3_).

***tert‐*** **Butyl((1*S*)‐2‐(2′‐((4‐methoxyphenyl)carbamoyl)spiro[cyclohexane‐1,3′‐indolin]‐1′‐yl)‐2‐oxo‐1‐phenylethyl)carbamate (12)**. According to general procedure A, a mixture of spiro[cyclohexane‐1,3′‐indole] **5** (100 mg, 0.54 mmol), Boc‐L‐α‐phenylglycine **7 c** (136 mg, 0.54 mmol) and 4‐metoxyphenyl isocyanide **6 a** (71 mg, 0.54 mmol) was dissolved in DCM (2 mL) in a sealed tube at 50 °C for 30 hours. The crude product was purified by chromatography on silica gel with hexane/ EtOAc (8 : 2) and by HPLC with MeOH/H_2_O (82 : 18) as eluent (flow rate 3.00 mL/min) to yield single diastereomers. Total yield: 60 % (*dr* 1 : 1).

(*S*,S*)‐**12**: yellowish solid; R_*f*_=0.43 (H : E=7 : 3). ^1^H NMR (400 MHz, DMSO‐*d*_6_) δ 10.40 (s, 1H), 7.96 (d, *J*=7.8 Hz, 1H), 7.69 (d, *J*=6.9 Hz, 1H), 7.44 (d, *J*=6.7 Hz, 2H), 7.33–7.30 (m, 2H), 7.25 (d, *J*=7.4 Hz, 2H), 7.16 (d, *J*=7.4 Hz, 2H), 7.08 (t, *J*=7.3 Hz, 1H), 6.88 (d, *J*=8.6 Hz, 2H), 5.72 (d, *J*=6.9 Hz, 1H), 5.55 (s, 1H), 3.76 (s, 3H), 2.04–1.83 (m, 4H), 1.77–1.65 (m, 6H), 1.44 (s, 9H). ^13^C NMR (101 MHz, DMSO) δ 170.1, 166.4, 156.1, 156.1, 142.8, 141.5, 135.9, 131.4, 128.7 (2), 128.4 (2), 128.2, 127.3, 124.2, 122.4, 121.8(2), 117.2, 114.2 (2), 79.1, 70.2, 56.6, 55.6, 49.1, 29.7, 28.6 (3), 25.5 (2), 23.1, 22.5. ESI‐MS *m/z*: [M+H]^+^ 570; *tR* HPLC=11.64 min. [α]^20^_D_=+62.1 (*c* 0.3, CHCl_3_).

(*S*,R*)‐**12**: yellowish solid; R_*f*_=0.38 (H : E=7 : 3). ^1^H NMR (400 MHz, Chloroform‐*d*) δ 8.31 (d, *J*=7.9 Hz, 1H), 7.49 (d, *J*=6.5 Hz, 2H), 7.39–7.35 (m, 2H), 7.30–7.27 (m, 2H), 7.22–7.12 (m, 4H), 6.80 (d, *J*=8.9 Hz, 2H), 5.72–5.64 (m, 1H), 4.62 (s, 1H), 3.76 (s, 3H), 1.78 (d, *J*=33.5 Hz, 6H), 1.65 (s, 1H), 1.57 (d, *J*=9.8 Hz, 1H), 1.41 (s, 9H), 1.14 (d, *J*=11.7 Hz, 2H). ^13^C NMR (101 MHz, CDCl_3_) δ 169.1, 166.6, 157.0, 154.7, 140.4, 139.7, 135.9, 129.6, 129.4 (2), 128.9, 128.5, 128.2 (2), 125.6, 123.5 (2), 122.7, 117.8, 114.0 (2), 79.9, 70.2, 58.1, 55.5, 49.5, 39.6, 30.2, 28.3 (3), 25.1, 22.6, 22.1. ESI‐MS *m/z*: [M+H]^+^ 570; *tR* HPLC=12.26 min. [a]^20^_D_=+28.31 (*c* 0.32, CHCl_3_).

***tert‐*** **Butyl((1*S*)‐2‐(2′‐(benzylcarbamoyl)spiro[cyclohexane‐1,3′‐indolin]‐1′‐yl)‐2‐oxo‐1‐phenylethyl)carbamate (13)**. According to general procedure A, a mixture of spiro[cyclohexane‐1,3′‐indole] **5** (100 mg, 0.54 mmol), Boc‐L‐α‐phenylglycine **7 c** (136 mg, 0.54 mmol) and benzyl isocyanide **6 b** (65 μL, 0.54 mmol) was dissolved in DCM (2 mL) in a sealed tube at 50 °C for 30 hours. The crude product was purified by chromatography on silica gel with hexane/ EtOAc (85 : 15) and by HPLC with MeOH/H_2_O (82 : 18) as the eluent (flow rate 3.00 mL/min) to yield single diastereomers. Total yield: 58 % (*dr* 1 : 1).

(*S*,S*)‐**13**: yellowish solid; R_*f*_=0.48 (H : E=7 : 3). ^1^H NMR (400 MHz, DMSO‐*d*_6_) δ 8.95 (s, 1H), 7.97 (d, *J*=7.6 Hz, 1H), 7.79 (d, *J*=7.3 Hz, 1H), 7.43–7.37 (m, 5H), 7.35–7.29 (m, 4H), 7.23–7.20 (m, 3H), 7.07 (t, *J*=7.3 Hz, 1H), 5.67 (d, *J*=7.3 Hz, 1H), 5.46 (s, 1H), 4.15 (d, *J*=14.0 Hz, 1H), 3.89 (d, *J*=13.9 Hz, 1H), 1.94–1.85 (m, 2H), 1.76 (d, *J*=12.7 Hz, 1H), 1.69–1.57 (m, 6H), 1.44 (s, 9H), 1.31–1.29 (m, 1H). ^13^C NMR (101 MHz, DMSO) δ 170.0, 168.1, 156.0, 142.7, 141.7, 138.8, 136.6, 128.6 (4), 128.5 (2), 128.5 (2), 128.2, 127.4, 127.2, 124.2, 122.2, 117.3, 79.0, 69.6, 56.3, 48.7, 43.0, 31.1, 29.6, 28.6 (3), 25.5, 23.2, 22.6. ESI‐MS *m/z*: [M+H]^+^ 554; *tR* HPLC=17.93 min. [α]^20^_D_=+85.33 (*c* 0.3, CHCl_3_).

(*S*,R*)‐**13**: yellowish solid; R_*f*_=0.39 (H : E=7 : 3). ^1^H NMR (400 MHz, DMSO‐*d*_6_) δ 9.13 (s, 1H), 8.11 (d, *J*=7.7 Hz, 1H), 7.57 (d, *J*=6.4 Hz, 2H), 7.43–7.32 (m, 9H), 7.21 (d, *J*=7.0 Hz, 1H), 7.16 (d, *J*=6.4 Hz, 1H), 7.06 (t, *J*=13.4 Hz, 1H), 5.53 (d, *J*=7.5 Hz, 1H), 4.73 (s, 1H), 4.44–4.32 (m, 2H), 2.15 (s, 9H), 1.52–1.42 (m, 10H). ^13^C NMR (101 MHz, DMSO) δ 169.0, 167.8, 155.1, 142.7, 140.7, 139.1, 137.3, 129.1 (2), 128.6 (4), 128.5, 128.2 (2), 127.5, 127.3, 124.3, 122.3, 116.7, 78.8, 68.6, 57.3, 48.6, 43.1, 31.1 (3), 28.6 (2), 25.3, 23.0, 22.1. ESI‐MS *m/z*: [M+H]^+^ 554; *tR* HPLC=18.92 min. [α]^20^_D_=+49.47 (*c* 0.3, CHCl_3_).

***tert‐*** **Butyl((1*S*)‐2‐(2′‐(cyclohexylcarbamoyl)spiro[cyclohexane‐1,3′‐indolin]‐1′‐yl)‐2‐oxo‐1‐phenylethyl)carbamate (14)**. According to general procedure A, a mixture of spiro[cyclohexane‐1,3′‐indole] **5** (100 mg, 0.54 mmol), Boc‐L‐α‐phenylglycine **7 c** (136 mg, 0.54 mmol) and cyclohexyl isocyanide **6 c** (66 μL, 0.54 mmol) was dissolved in DCM (2 mL) in a sealed tube at 50 °C for 30 hours. The crude product was purified by chromatography on silica gel with hexane/ EtOAc (92 : 8) to yield the single diastereoisomers. The products were subjected to HPLC with MeOH/H_2_O (85 : 15) as the eluent (flow rate 3.00 mL/min) for purity determination. Total yield: 60 % (*dr* 1 : 1).

(*S*,S*)‐**14**: yellowish solid; R_*f*_=0.56 (H : E=7 : 3). ^1^H NMR (400 MHz, DMSO‐*d*_6_) δ 8.51 (d, *J*=7.6 Hz, 1H), 8.10 (d, *J*=7.9 Hz, 1H), 7.59 (d, *J*=7.1 Hz, 2H), 7.45–7.39 (m, 3H), 7.28 (d, *J*=7.9 Hz, 1H), 7.23–7.15 (m, 2H), 7.05 (t, *J*=7.4 Hz, 1H), 5.52 (d, *J*=8.0 Hz, 1H), 4.73 (s, 1H), 3.67–3.59 (m, 1H), 1.86–1.62 (m, 10H), 1.44 (s, 9H), 1.35–1.18 (m, 8H), 0.96 (t, *J*=18.9 Hz, 2H). ^13^C NMR (101 MHz, DMSO) δ 169.2, 166.4, 154.9, 142.8, 140.9, 137.9, 128.9 (2), 128.6, 128.2 (2), 127.5, 124.2, 122.3, 116.5, 78.7, 68.9, 56.9, 48.4, 48.2, 32.6, 32.4, 29.7, 28.5 (4), 25.7, 25.4, 25.2, 25.1, 23.0, 21.9. ESI‐MS *m/z*: [M+H]^+^ 546; *tR* HPLC=12.93 min. [α]^20^_D_=+47.60 (*c* 0.3, CHCl_3_).

(*S*,R*)‐**14**: yellowish solid; R_*f*_=0.52 (H : E=7 : 3). ^1^H NMR (400 MHz, DMSO‐*d*_6_) δ 8.42 (d, *J*=6.6 Hz, 1H), 7.93 (d, *J*=7.8 Hz, 1H), 7.73 (d, *J*=7.4 Hz, 1H), 7.48–7.42 (m, 2H), 7.38–7.32 (m, 3H), 7.23–7.15 (m, 3H), 7.06 (t, *J*=7.3 Hz, 1H), 5.70 (d, *J*=7.5 Hz, 1H), 5.41 (s, 1H), 3.31–3.24 (m, 1H), 1.82–1.60 (m, 10H), 1.43 (s, 9H), 1.32–1.28 (m, 2H), 1.20–1.12 (m, 7H). ^13^C NMR (101 MHz, DMSO) δ 170.1, 167.0, 156.1, 142.8, 142.0, 136.6, 128.5, 128.4 (2), 128.2, 127.1, 124.21, 122.1, 117.2, 79.1, 69.5, 56.2, 48.7, 48.0, 32.2, 31.8, 29.6, 29.4, 28.5 (4), 25.6 (2), 24.8, 24.5, 23.4, 22.6. ESI‐MS *m/z*: [M+H]^+^ 546; *tR* HPLC=12.32 min. [α]^20^_D_=+56.33 (*c* 0.3, CHCl_3_).

**Benzyl((2*S*)‐1‐(2′‐(benzylcarbamoyl)spiro[cyclohexane‐1,3′‐indolin]‐1′‐yl)‐1‐oxo‐3‐phenylpropan‐2‐yl)carbamate (15)**. According to general procedure A, a mixture of spiro[cyclohexane‐1,3′‐indole] **5** (100 mg, 0.54 mmol), Z‐L‐Phenylalanine **7 b** (161 mg, 0.54 mmol) and benzyl isocyanide **6 b** (65 μL, 0,54 mmol) was dissolved in DCM (2 mL) in a sealed tube at 50 °C for 30 hours. The crude product was purified by chromatography on silica gel with hexane/ EtOAc (8 : 2) and by HPLC with MeOH/H_2_O (88 : 12) as eluent (flow rate 3.00 mL/min) to afford an inseparable mixture of diastereomers (74 % total yield). Yellowish oil; R_*f*_=0.64 (H : E=7 : 3). ^1^H NMR (400 MHz, DMSO‐*d*_6_) δ 9.06 (s, 1H), 8.97 (s, 1H), 8.08 (d, *J*=7.9 Hz, 1H), 8.02 (d, *J*=7.8 Hz, 1H), 7.93 (d, *J*=7.8 Hz, 1H), 7.56 (d, *J*=8.4 Hz, 1H), 7.35 (s, 2H), 7.30–7.21 (m, 25H), 7.21–7.17 (m, 4H), 7.15–7.12 (m, 2H), 7.06–6.98 (m, 3H), 5.35 (s, 1H), 5.05 (s, 2H), 4.95 (s, 2H), 4.82 (d, *J*=7.8 Hz, 1H), 4.71 (s, 1H), 4.63–4.56 (m, 1H), 4.41–4.33 (m, 2H), 4.13–4.01 (m, 2H), 2.98–2.88 (m, 3H), 2.76–2.69 (m, 1H), 1.84–1.74 (m, 3H), 1.64–1.49 (m, 12H), 1.30–1.19 (m, 4H), 1.07–1.03 (m, 1H). ^13^C NMR (101 MHz, DMSO) δ 171.2, 170.3, 168.7, 167.7, 156.6, 155.7, 142.6, 141.4, 141.1, 139.1, 138.7, 137.9, 137.6, 137.5, 137.2, 136.9, 129.8, 129.7, 128.7 (2), 128.7 (2), 128.7 (3), 128.7 (3), 128.6 (3), 128.5 (3), 128.4 (3), 128.2, 128.1, 128.0 (3), 127.5, 127.4, 127.4, 127.3, 126.9, 126.8, 124.2, 124.2, 122.4, 122.3, 117.1, 116.8, 69.7, 69.4, 65.9, 65.7, 54.4, 54.4, 48.6, 48.2, 43.4, 43.2, 39.2 (3), 36.5, 29.8, 29.7, 25.6, 25.5, 23.1 (2), 22.5, 22.3. ESI‐MS *m/z*: [M+H]^+^ 602; *tR* HPLC=14.51 min.

**Benzyl((2*S*)‐1‐(2′‐(cyclohexylcarbamoyl)spiro[cyclohexane‐1,3′‐indolin]‐1′‐yl)‐1‐oxopropan‐2‐yl)carbamate (16)**. According to general procedure A, a mixture of spiro[cyclohexane‐1,3′‐indole] **5** (100 mg, 0.54 mmol), Z‐L‐Alanine **7 a** (120 mg, 0.54 mmol) and cyclohexyl isocyanide **6 c** (66 μL, 0.54 mmol) was dissolved in DCM (2 mL) in a sealed tube at 50 °C for 30 hours. The crude product was purified by chromatography on silica gel with hexane/ EtOAc (8 : 2) and by HPLC with MeOH/H_2_O (85 : 15) as eluent (flow rate 3.00 mL/min) to afford an inseparable mixture of diastereomers. Total yield: 65 %; yellowish oil; R_*f*_=0.51 (H : E=7 : 3). ^1^H NMR (400 MHz, DMSO‐*d*_6_) δ 8.53 (d, *J*=7.6 Hz, 1H), 8.41 (d, *J*=7.7 Hz, 1H), 7.99–7.92 (m, 2H), 7.82 (d, *J*=6.1 Hz, 1H), 7.44 (d, *J*=7.5 Hz, 1H), 7.38–7.31 (m, 9H), 7.18–7.11 (m, 5H), 7.02–6.98 (m, 2H), 5.12 (s, 1H), 5.05–4.95 (m, 4H), 4.79 (s, 1H), 4.51–4.43 (m, 2H), 3.57–3.50 (m, 2H), 1.87–1.80 (m, 4H), 1.74–1.62 (m, 19H), 1.55 (d, *J*=11.5 Hz, 5H), 1.30 (d, *J*=6.8 Hz, 4H), 1.26–1.19 (m, 9H), 1.14 (d, *J*=6.9 Hz, 5H). ^13^C NMR (101 MHz, DMSO) δ 172.3, 171.7, 167.4, 166.3, 156.4, 155.7, 142.9, 142.9, 141.5, 141.2, 137.5, 137.4, 128.8 (2), 128.7 (2), 128.2 (4), 128.1 (2), 127.3, 127.2, 123.9, 123.8, 122.2, 122.1, 116.7, 116.5, 69.5, 68.3, 65.8, 65.7, 49.2, 48.6, 48.3, 48.3, 47.9, 47.9, 32.7, 32.5, 32.4, 32.3, 31.1, 29.8, 29.7, 25.6 (2), 25.6 (2), 25.0, 24.8 (2), 24.7, 23.4, 23.3, 22.4, 22.2, 19.0, 17.1 (2). ESI‐MS *m/z*: [M+H]^+^ 518; *tR* HPLC=12.47 min.

***S*** **‐((2*S*)‐2‐methyl‐3‐oxo‐3‐(2′‐(tritylcarbamoyl)spiro[cyclohexane‐1,3′‐indolin]‐1′‐yl)propyl) ethanethioate (17**). According to general procedure A, a mixture of spiro[cyclohexane‐1,3′‐indole] 5 (100 mg, 0.54 mmol), (*S*)‐3‐(acetylthio)‐2‐methylpropanoic acid **7 d** (74 μL, 0.54 mmol) and triphenylmethyl (trityl) 6d (159 mg, 0.59 mmol) was dissolved in DCM (2 mL) in a sealed tube at 50 °C for 30 hours. The crude product was purified by chromatography on silica gel with hexane/ EtOAc (8 : 2) and by HPLC with MeOH/H_2_O (85 : 15) as the eluent (flow rate 3.00 mL/min) to yield single diastereomers. Total yield: 57 % (*dr* 1 : 1).

(*S*,S*)‐**17**: yellowish solid; R_*f*_=0.58 (H : E=7 : 3). ^1^H NMR (700 MHz, Chloroform‐*d*) δ 8.07 (d, J=7.6 Hz, 1H), 7.12–7.09 (m, 8H), 7.07–7.00 (m, 5H), 6.97–6.92 (m, 5H), 6.71 (d, 1H), 4.57 (s, 1H), 3.27 (d, J=16.6 Hz, 1H), 3.13 (q, J=25.5, 7.4 Hz, 1H), 2.84 (d, 1H), 2.00 (s, 3H), 1.64–1.52 (m, 6H), 1.45–1.38 (m, 2H), 1.28 (d, J=6.5 Hz, 3H), 1.20–1.13 (m, 2H). ^13^C NMR (176 MHz, Chloroform‐*d*) δ 194.37, 172.33, 166.55, 143.07 (3), 139.36, 139.02, 127.63 (6), 127.12 (6), 126.85 (3), 125.96, 124.34, 121.46, 116.99, 69.75, 69.20, 48.44, 39.26, 37.80, 30.35, 29.54, 28.17, 24.34, 21.89, 21.39, 17.11. *tR* HPLC=14.95 min. [α]^20^_D_=+12.63 (*c* 0.3, CHCl_3_).

(*S*,R*)‐**17**: yellowish solid; R_*f*_=0.58 (H : E=7 : 3). ^1^H NMR (700 MHz, Chloroform‐*d*) δ 8.10 (d, J=7.5 Hz, 1H), 7.17–7.13 (m, 12H), 7.01–6.97 (m, 6H), 6.89 (d, 1H), 5.13 (s, 1H), 3.22 (dd, J=13.4, 5.5 Hz, 1H), 2.91 (d, 1H), 2.79 (d, J=11.5 Hz, 1H), 2.24 (s, 3H), 1.75–1.60 (m, 9H), 1.29–1.26 (m, 1H), 1.18 (d, J=5.8 Hz, 3H). ^13^C NMR (176 MHz, Chloroform‐*d*) δ 196.41, 173.01, 167.61, 144.14 (3), 141.12, 140.42, 128.31 (6), 127.88 (6), 127.05 (4), 125.10, 122.42, 118.20, 71.33, 70.26, 49.37, 39.08, 38.81, 33.77, 30.63, 29.76, 25.55, 23.19, 22.88, 16.61. *tR* HPLC=15.97 min. [α]^20^_D_=−14.37 (*c* 0.3, CHCl_3_).

### Antibiotics and strains

Vancomycin, tobramycin, oxacillin, imipenem, and amphotericin B were purchased from Sigma‐Aldrich (Milan, Italy). *Staphylococcus aureus* ATCC 43300, *S. epidermidis* ATCC 35984, *Pseudomonas aeruginosa* ATCC 27853, *Klebsiella pneumoniae* ATCC BAA‐1705 (carbapenemase producer), and *Candida albicans* ATCC 90028 were obtained from the American Type Culture Collection (Rockville, MD).

***Antimicrobial susceptibility testing*** Minimal inhibitory concentrations (MIC) of all the compounds were determined in Mueller‐Hinton medium (MH) by the broth microdilution assay, following the procedure already described.^[55]^ The compounds were added to bacterial suspension in each well yielding a final cell concentration of 1×10^6^ CFU/mL and a final compound concentration ranging from 3.1 to 100 μM. Negative control wells were set to contain bacteria in Mueller‐Hinton broth plus the amount of vehicle (DMSO) used to dilute each compound. Positive controls included vancomycin (2 μg/mL), tobramycin (1 and 4 μg/mL), oxacillin (2 and 10 μg/mL), and imipenem (4 and 8 μg/mL). The MIC was defined as the lowest concentration of drug that caused a total inhibition of microbial growth after 24 hours incubation time at 37 °C. Medium turbidity was measured by a microtiter plate reader (Bio‐Rad mod 680, Milan, Italy) at 595 nm.

*Candida albicans* ATCC 90028 The antifungal activity of compounds **8**–**16** was determined using a standardized broth microdilution method (Clinical and Laboratory Standards Institute (CLSI) document M27‐A2).^[56]^ Briefly, cell suspension was adjusted to 3×10^3^ CFU/mL in RPMI 1640 medium (Sigma) supplemented with 0.2 % (w/v) glucose. One hundred microliter aliquots of these cell suspensions were dispensed into 96‐well microtiter plates. Compounds were serially diluted using RPMI 1640 medium and added to the wells at a final concentration ranging from 3.1 to 100 μM, and the plate was incubated for 48 hours at 37 °C. Amphotericin B (2 μg/mL) was chosen as the positive control. All the tests were conducted at least three times using independent cell suspensions.

### Biofilm formation assay

Biofilm formation was evaluated by measuring the ability of cells to adhere to sterile 96‐well polystyrene flat‐bottom microtiter plate (BD Falcon, Mississauga, Ontario Canada) as described previously.^[57]^ Briefly, a suspension of *S. epidermidis* (MH supplemented with 1 % glucose) at the final density of 10^5^ CFU mL^−1^ was treated with compound (S,S*)‐**9**, (S,R*)‐**11** and **15** at 100 μM. After 24 hours at 37 °C, planktonic cells were removed, and the wells washed twice with phosphate‐buffered saline (PBS) and dried at 60 °C for 30 min. Crystal violet solution (150 mL at 0.1 %) was added to each well and the plates were incubated at room temperature for 30 min. The wells were then washed with PBS and discolored with 200 mL of 96 % ethanol for 20 min. Absorbance was measured at 595 nm using a microtiter plate reader. The percentage of biofilm mass reduction was calculated using the formula: [(Ac−At)/Ac]×100, where Ac is the OD_595_ for control wells and At is OD_595_ in the presence of a compound.

### Cell culture

HaCaT cells, an immortalized, nontumorigenic human keratinocyte cell line purchased from ATCC, were cultured in Dulbecco's modified Eagle's medium (DMEM; Invitrogen, Paisley, United Kingdom) containing high glucose (4.5 g/L), supplemented with 10 % fetal bovine serum (FBS; Cambrex, Verviers, Belgium), l‐glutamine (2 mM; Sigma, Milan, Italy), penicillin (100 U/mL; Sigma), and streptomycin (100 mg/mL; Sigma) at 37 °C in a humidified 5 % CO_2_ atmosphere, according to ATCC recommendations.^[58]^

### In vitro bioscreens

The biological effects and cytotoxicity of (S,S*)‐**9**, (S,S*)‐**11**, (S,R*)‐**11** and (*S,R/S*)‐**15** were investigated through the estimation of a “cell survival index”, arising from the combination of cell viability evaluation and automatic cell count.^[59]^ Cells were inoculated in 96‐microwell culture plates at a density of 10^4^ cells/well and allowed to grow for 24 h. The medium was then replaced with fresh medium and cells were treated for further 48 h with a range of concentrations (25→400 μM) of (S,S*)‐**9**, (S,S*)‐**11**, (S,R*)‐**11** and (*S,R/S*)‐**15**. DMSO was used as vehicle to solubilize compounds under investigation to a final concentration of 100 mM. DMSO toxicity was evaluated in control cultures at a concentration ranging from 0.025 to 0.5 % v/v (the same concentrations used in bioscreen experiments) to exclude interference with cell viability. Cell viability was evaluated by the MTT assay procedure, which measures the level of mitochondrial dehydrogenase activity using the yellow 3‐(4,5‐dimethyl‐2‐thiazolyl)‐2,5‐diphenyl‐2*H*‐tetrazolium bromide (MTT, Sigma) as substrate. The assay is based on the redox ability of living mitochondria to convert dissolved MTT into insoluble purple formazan. Briefly, after the treatments, the medium was removed, and the cells were incubated with 20 μL/well of a MTT solution (5 mg/mL) for 1 h in a humidified 5 % CO_2_ incubator at 37 °C. The incubation was stopped by removing the MTT solution and by adding 100 μL/well of DMSO to solubilize the formazan. Finally, the absorbance was monitored at 550 nm by using a microplate reader (iMark microplate reader, Bio‐Rad, Milan, Italy). Cell number was determined by TC20 automated cell counter (Bio‐Rad, Milan, Italy), providing an accurate and reproducible total count of cells and a live/dead ratio in one step by a specific dye (trypan blue) exclusion assay. Bio‐Rad's TC20 automated cell counter uses disposable slides, trypan blue dye (0.4 % trypan blue dye w/v in 0.81 % sodium chloride and 0.06 % potassium phosphate dibasic solution) and a CCD camera to count cells based on the analyses of captured images. Once the loaded slide is inserted into the slide port, the TC20 automatically focuses on the cells, detects the presence of trypan blue dye and provides the count. When cells are damaged or dead, trypan blue can enter the cell allowing living cells to be counted. Operationally, after treatments in 96‐microwell culture plates, the medium was removed, and the cells were collected. Ten microliters of cell suspension, mixed with 0.4 % trypan blue solution at 1 : 1 ratio, were loaded into the chambers of disposable slides. The results are displayed as total cell count (number of cells per ml). If trypan blue is detected, the instrument also accounts for the dilution and shows live cell count and percent viability. Total counts and live/dead ratio from random samples for each cell line were subjected to comparisons with manual hemocytometers in control experiments. The calculation of the concentration required to inhibit the net increase in the cell number and viability by 50 % (IC_50_) is based on plots of data (*n*=4 for each experiment) and repeated three times (total *n*=12). IC_50_ values were obtained by means of a dose response curve by nonlinear regression using a curve fitting program, GraphPad Prism 5.0, and are expressed as mean±SEM (*n*=12) of five independent experiments.

## Conflict of interest

The authors declare no conflict of interest.

## Biographical Information

*Antonella Ilenia Alfano is currently pursuing her PhD in the SPOTS‐Lab at the Department of Pharmacy, University of Naples Federico II. She graduated in 2018 in Pharmaceutical Chemistry and Technology. Soon afterward she spent a six‐month fellowship working on the multicomponent synthesis of compounds via visible‐light photocatalysis approaches. Her PhD project focuses on the development of greener and more sustainable syntheses and derivatization of privileged scaffolds using continuous flow chemistry. She is currently focusing on spirocyclic constrained compounds as structural templates for the generation of diversity‐oriented libraries*.

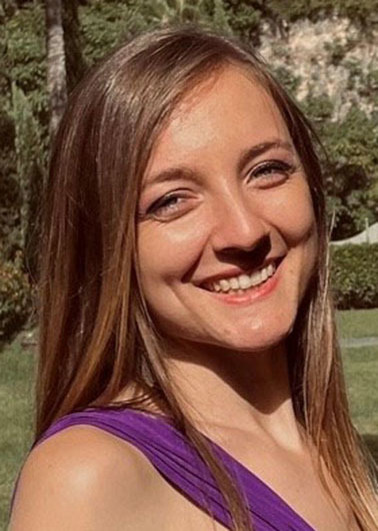

## Biographical Information

*Margherita Brindisi received her PhD in Pharmaceutical Sciences from the University of Siena in 2008. As a temporary researcher in Siena, she worked on developing compounds for the treatment of cancer, viral and parasitic diseases, and brain disorders. She was a postdoctoral fellow in Professor Arun K. Ghosh's research group at Purdue University in 2010–2011 and a Visiting Scientist in the same group in 2016–2017. In April 2019, she was appointed as Assistant Professor at the Department of Pharmacy at University of Naples Federico II, where she is working on the application of sustainable methodologies to her drug discovery projects*.

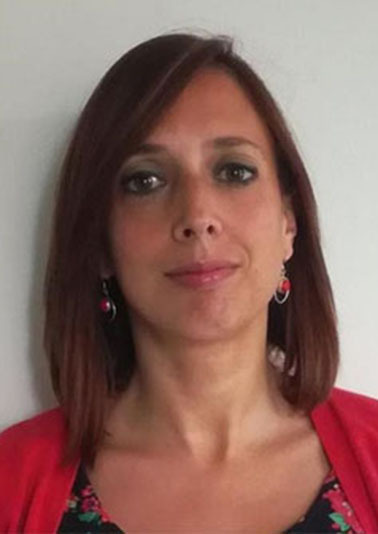

## Supporting information

As a service to our authors and readers, this journal provides supporting information supplied by the authors. Such materials are peer reviewed and may be re‐organized for online delivery, but are not copy‐edited or typeset. Technical support issues arising from supporting information (other than missing files) should be addressed to the authors.

Supporting Information
