# Supplementary material for: Coupling Interrupted Fischer and Multicomponent Joullié‐Ugi to Chase Chemical Diversity: from Batch to Sustainable Flow Synthesis of Peptidomimetics
Source: ChemMedChem. 2021 Oct 18;16(24):3795–809. doi: 10.1002/cmdc.202100474 (PMC9297956; doi:10.1002/cmdc.202100474)

ChemMedChem

Supporting Information

Coupling Interrupted Fischer and Multicomponent Joullié-Ugi to Chase Chemical Diversity: from Batch to Sustainable Flow Synthesis of Peptidomimetics

Antonella Ilenia Alfano, Elisabetta Buommino, Maria Grazia Ferraro, Carlo Irace, Angela Zampella, Heiko Lange,* and Margherita Brindisi*

SUPPORTING INFORMATION

Table of Contents

General procedure of trityl isocyanide synthesis…………………………………Page S2

Table S1……………………………………………………………………………. Page S3

ESI-MS, NMR spectra and HPLC chromatograms for compounds 8-17………Pages S4-S31

Synthesis of *N*-trityl isocyanide

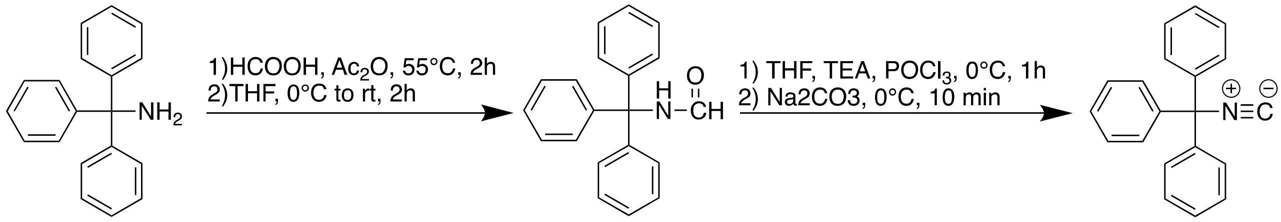

N-Trityl formamide

Formic acid (10.4 mmol, 2.7 equiv.) and acetic anhydride (8.85 mmol, 2.3 equiv.) are reacted in a dry flask at 55 °C for 2 h. The solution is transferred dropwise to a solution of trityl amine (3.85 mmol, 1 equiv.) in 7 mL THF (0.6 M) at 0 °C under N_2 atmosphere. The reaction mixture is heated to room temperature and stirred for 2 h. The reaction is quenched with saturated Na_2CO_3 solution and the aqueous layer is extracted with AcOEt. The white solid is dried to afford the pure product. Yield: quantitative.

Trityl isocyanide

Trityl formamide (4.11 mmol, 1 equiv) and triethylamine (27.54 mol, 6.7 equiv) are dissolved in 7 mL THF (0.6 M) and cooled to 0 °C under N_2 atmosphere. Phosphorus oxychloride (6.99 mmol, 1.7 equiv) is added dropwise to reaction mixture and is stirred for 1 h. The solution is then transferred to an extraction funnel and washed with saturated $NaHCO_3$ solution and the aqueous layer is extracted with CH_2Cl_2. The product is purified by chromatography on silica gel column (95:5 Hex/AcOEt). Yield: 88%.

Table S1. In vitro antibacterial activity. MIC values (µM) for synthesized compounds against *Staphylococcus aureus*, *S. epidermidis*, *Pseudomonas aeruginosa*, *Klebsiella pneumoniae, Candida albicans.*

Compounds	*S.aureus* ATCC 43300	*S. epidermidis* ATCC 35984	*P. aeruginosa* ATCC 27853	*K.pneumoniae* ATCC BAA-1705	*Candida albicans ATCC 90028*
***(S,S*)*-8**	>100	>100	>100	>100	>100
***(S,R*)*-8**	>100	>100	>100	>100	>100
***(S,S*)*-9**	>100	>100	>100	>100	>100
***(S,R*)*-9**	>100	>100	>100	>100	>100
***(S,S*)*-10**	>100	>100	>100	>100	>100
***(S,R*)*-10**	>100	>100	>100	>100	>100
**15**	>100	>100	>100	>100	>100
**16**	>100	>100	>100	>100	>100
***(S,S*)*-11**	>100	>100	>100	>100	>100
***(S,R*)*-11**	>100	>100	>100	>100	>100
***(S,S*)*-12**	>100	>100	>100	>100	>100
***(S,R*)*-12**	>100	>100	>100	>100	>100
***(S,S*)*-13**	>100	>100	>100	>100	>100
***(S,R*)*-13**	>100	>100	>100	>100	>100
***(S,S*)*-14**	>100	>100	>100	>100	>100
***(S,R*)*-14**	>100	>100	>100	>100	>100
**Oxacillin**	10	2	NA	NA	NA
**Vancomycin**	2	2	NA	NA	NA
**Tobramycin**	1	1	4	4	NA
**Imipenem**	NT	NT	4	>8	NA
**Amphotericin B**	NA	NA	NA	NA	2

*NA= not applicable; NT= Not tested. For oxacillin, vancomycin, tobramycin and imipenem MIC values are expressed as µg/mL.

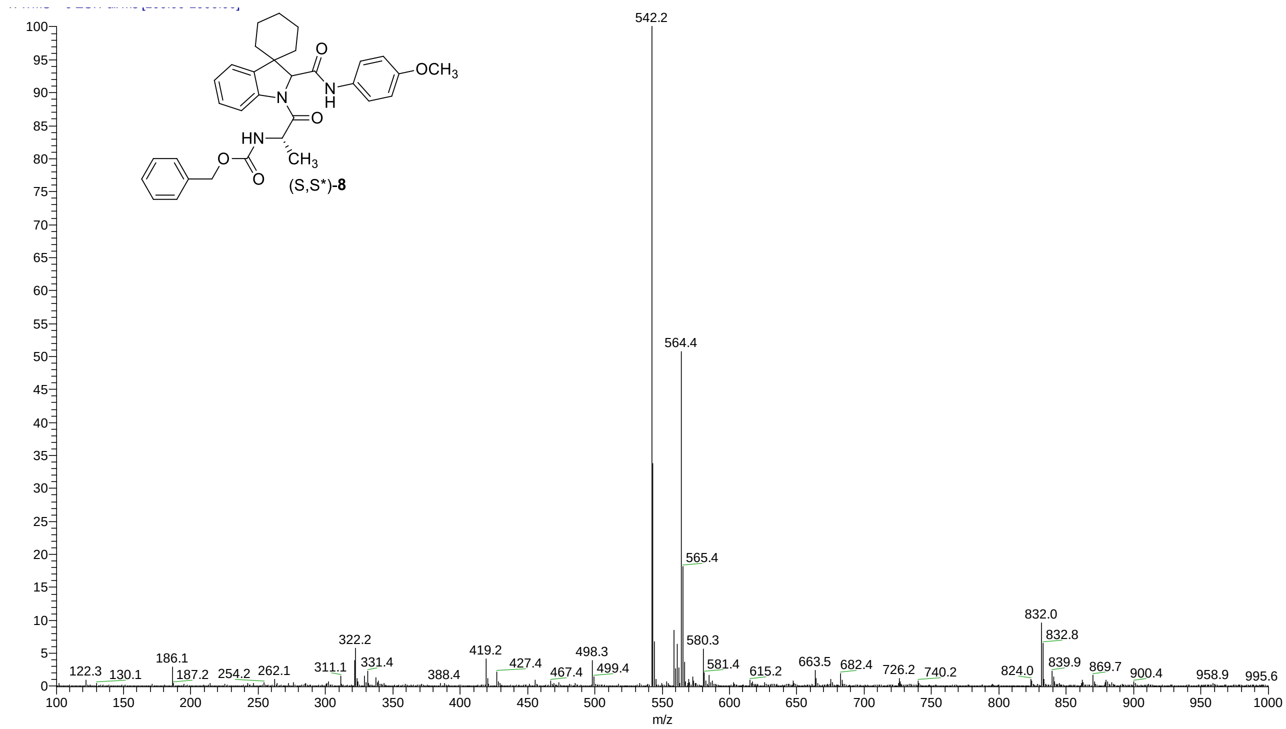

542.2
564.4
565.4
832.0
832.8
580.3
322.2
419.2
498.3
186.1
663.5
m/z
OCH3
HN
CH3
(S,S*)-8

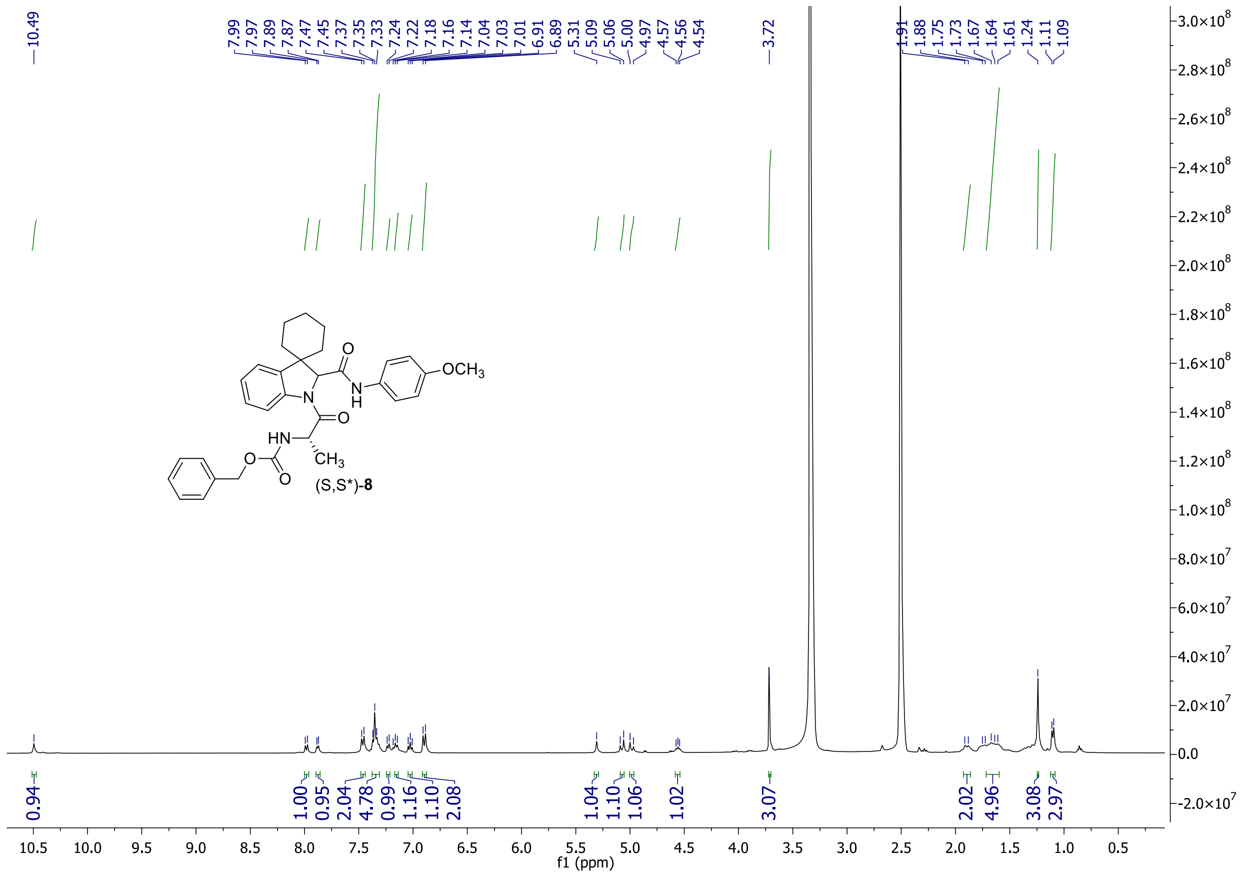

10.49
7.99 7.97 7.89 7.87 7.47 7.45 7.37 7.35 7.33 7.24 7.22 7.18 7.16 7.14 7.04 7.03 7.01 6.91 6.89
5.31 5.09 5.06 5.00 4.97 4.57 4.56 4.54
3.72
1.91 1.88 1.75 1.73 1.67 1.64 1.61 1.24 1.11 1.09
0.94 1.00 0.95 2.04 4.78 0.99 1.16 1.10 2.08 1.04 1.10 1.06 1.02 3.07 2.02 4.96 3.08 2.97
f1 (ppm)
OCH3
HN
CH3
(S,S*)-8

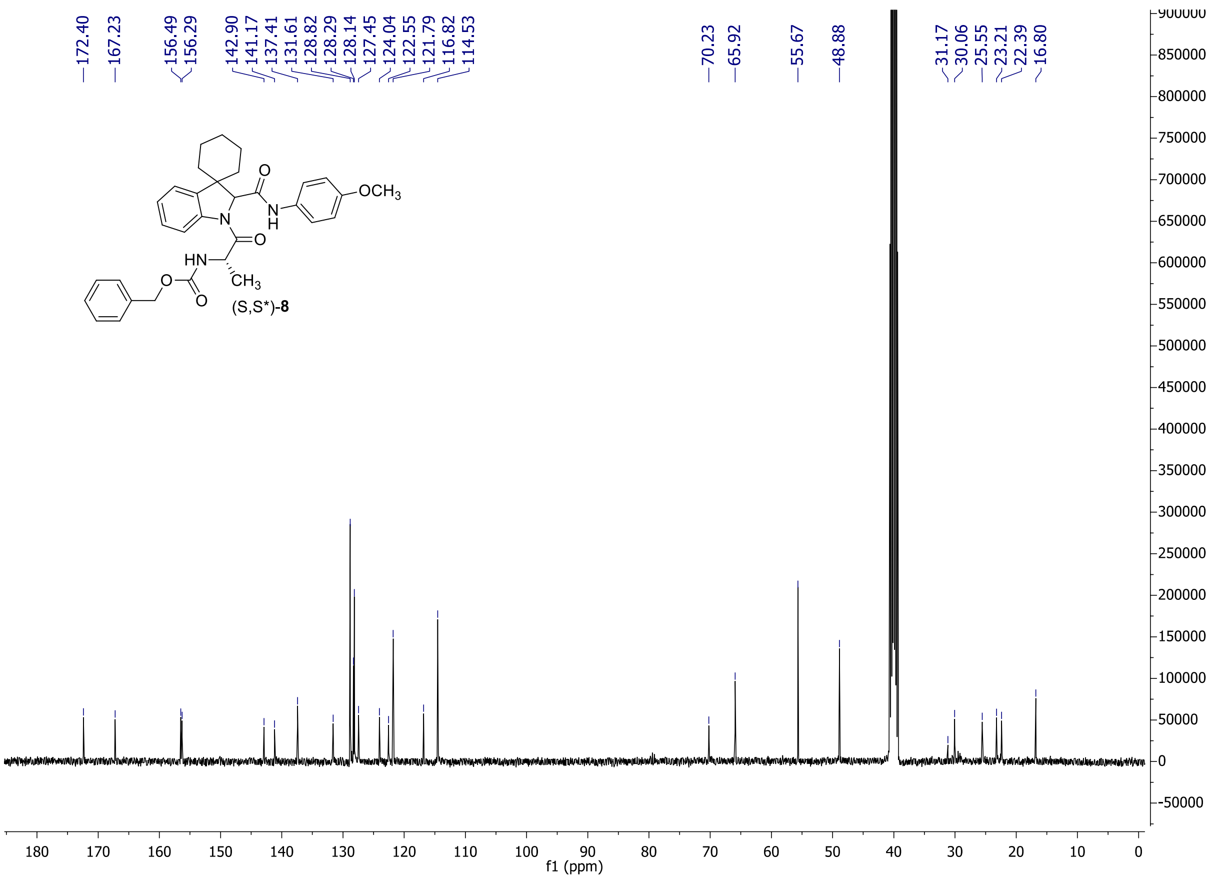
172.40
167.23
156.49
156.29
142.90
141.17
137.41
131.61
128.82
128.29
128.14
127.45
124.04
122.55
121.79
116.82
114.53
70.23
65.92
55.67
48.88
31.17
30.06
25.55
23.21
22.39
16.80
OCH3
(S,S*)-8
f1 (ppm)

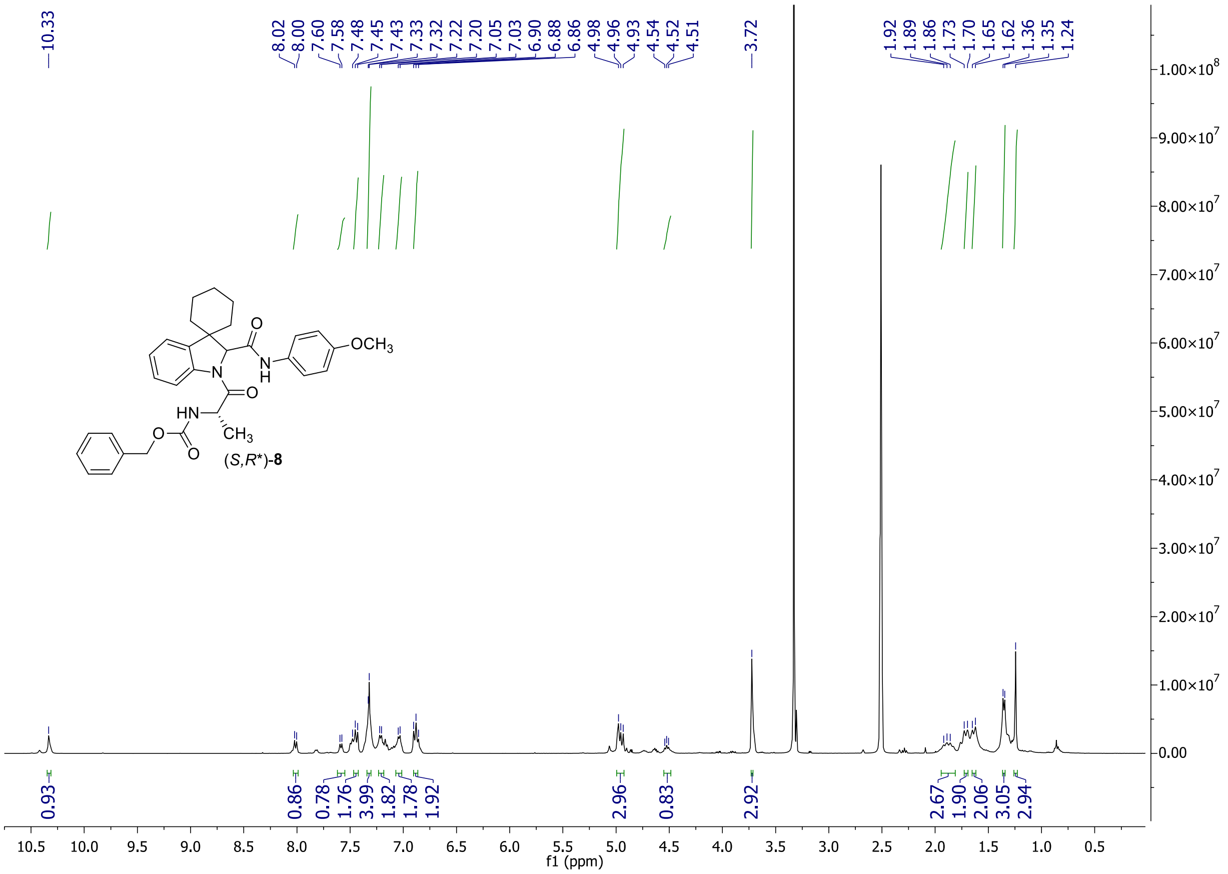
10.33
8.02 8.00 7.60 7.58 7.48 7.45 7.43 7.33 7.32 7.22 7.20 7.05 7.03 6.90 6.88 6.86
4.98 4.96 4.93 4.54 4.52 4.51
3.72
1.92 1.89 1.86 1.73 1.70 1.65 1.62 1.36 1.35 1.24
OCH3
HN
N
H
O
CH3
(S,R*)-8
0.93 0.86 0.78 1.76 3.99 1.82 1.78 1.92 2.96 0.83 2.92 2.67 1.90 2.06 3.05 2.94
f1 (ppm)

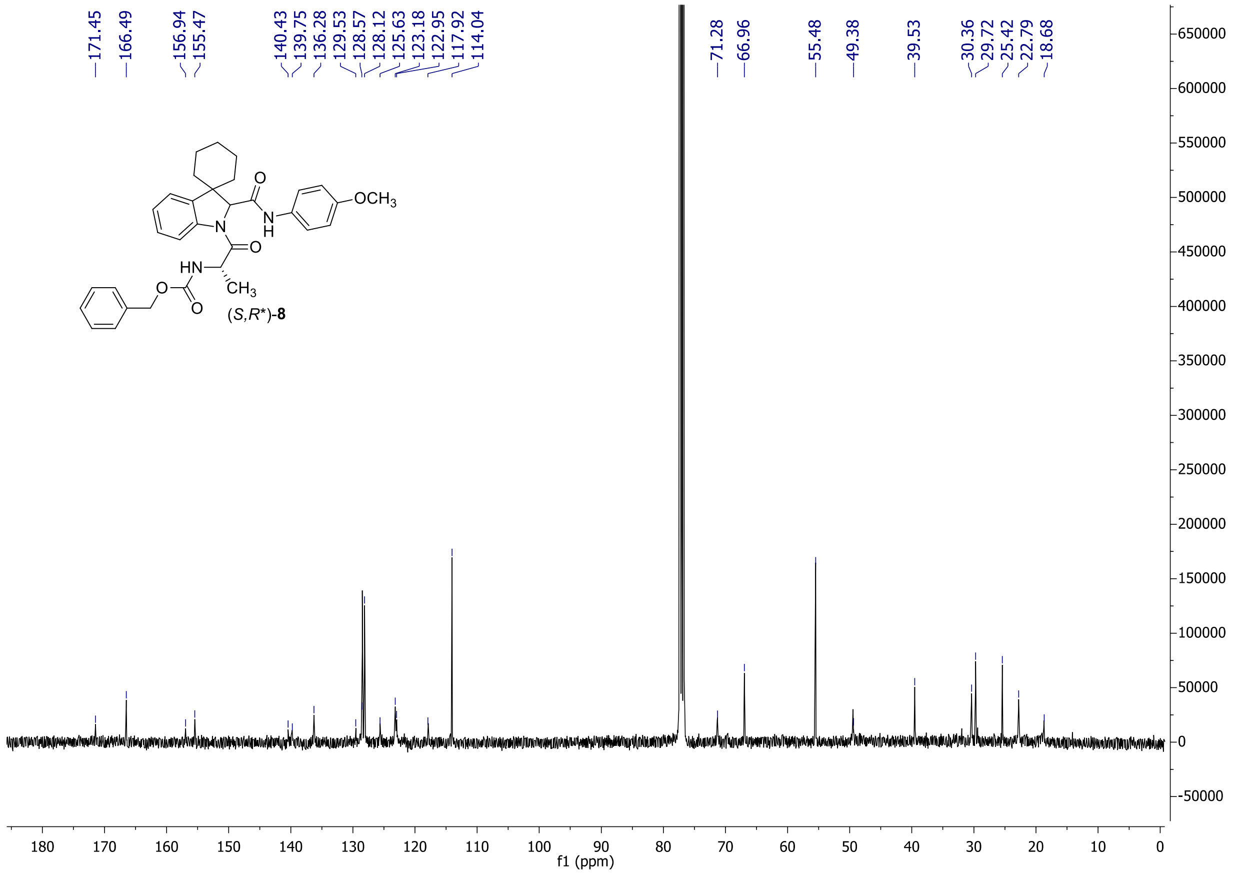
171.45 166.49 156.94 155.47
140.43 139.75 136.28 129.53 128.57 128.12 125.63 123.18 122.95 117.92 114.04
71.28 66.96 55.48 49.38 39.53 30.36 29.72 25.42 22.79 18.68
OCH3
HN
N
H
O
CH3
(S,R*)-8
f1 (ppm)

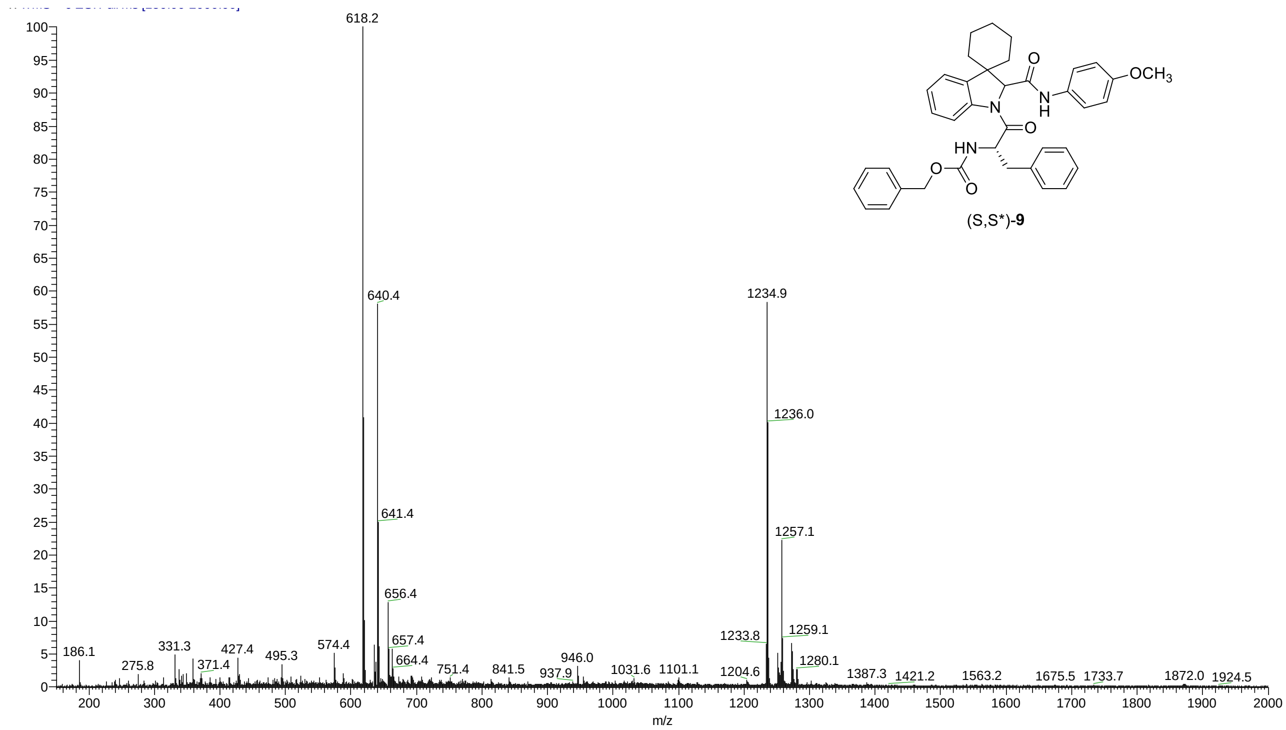
618.2
640.4
641.4
656.4
657.4
664.4
1234.9
1236.0
1257.1
1259.1
1280.1
1233.8
m/z
OCH3
(S,S*)-9

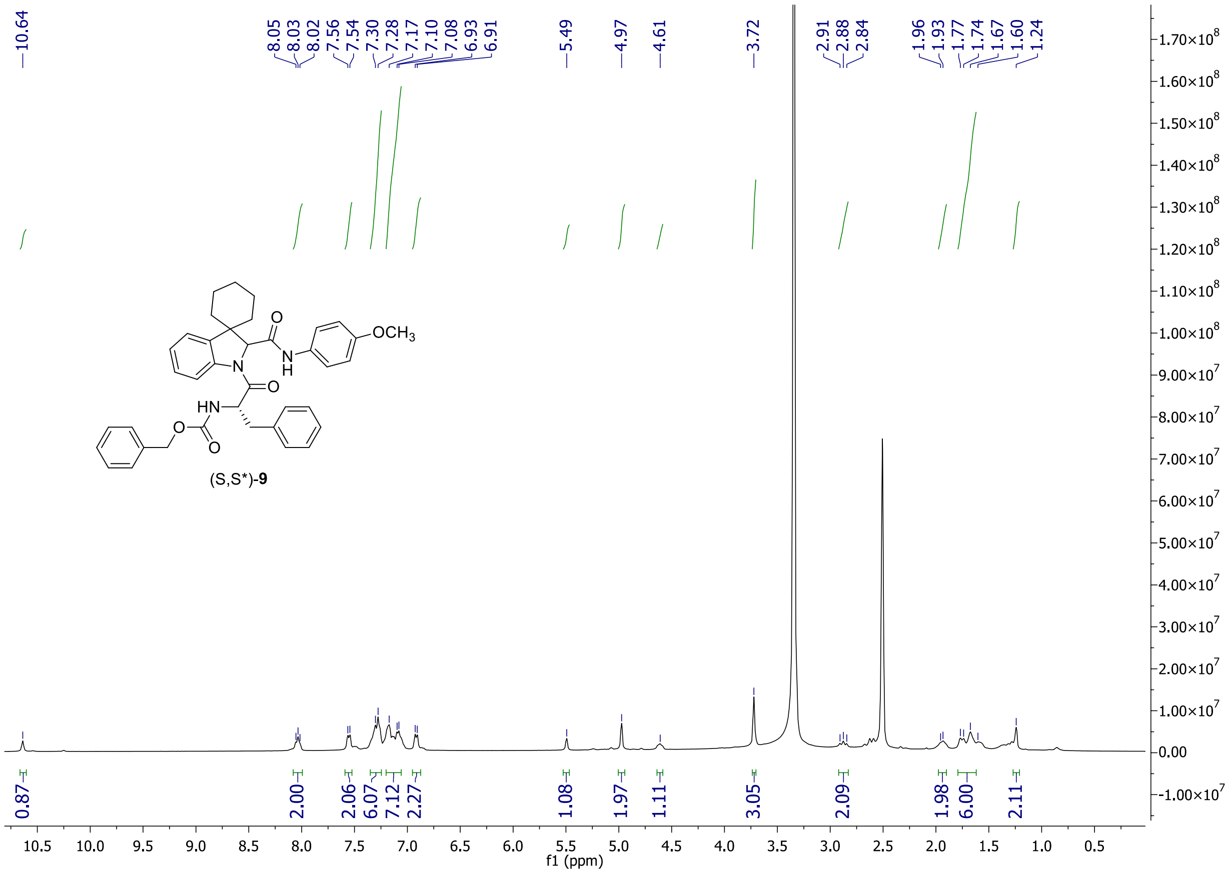
10.64
8.05 8.03 8.02 7.56 7.54 7.30 7.28 7.17 7.10 7.08 6.93 6.91
5.49
4.97
4.61
3.72
2.91 2.88 2.84
1.96 1.93 1.77 1.74 1.67 1.60 1.24
0.87 2.00 2.06 6.07 7.12 2.27 1.08 1.97 1.11 3.05 2.09 1.98 6.00 2.11
f1 (ppm)
OCH3
(S,S*)-9

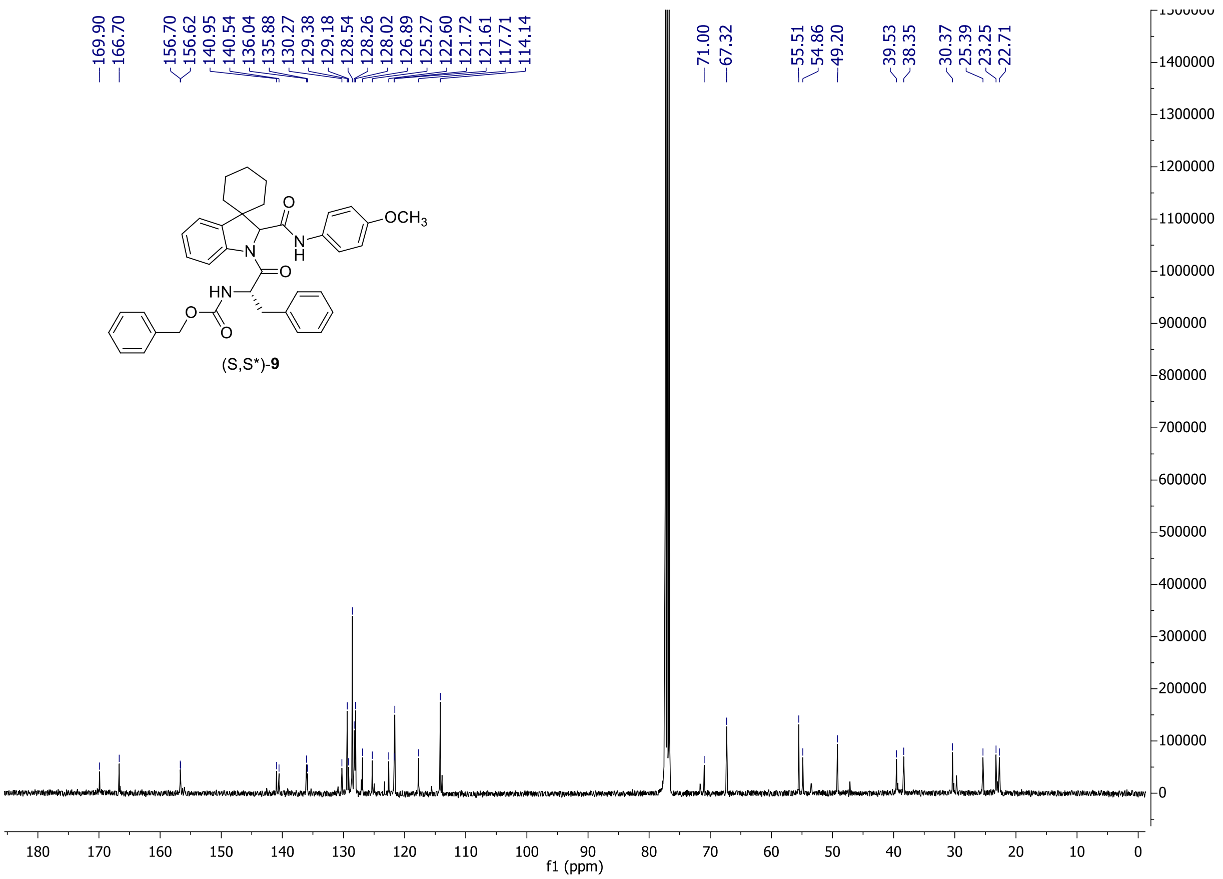
169.90
166.70
156.70
156.62
140.95
140.54
136.04
135.88
130.27
129.38
129.18
128.54
128.26
128.02
126.89
125.27
122.60
121.72
121.61
117.71
114.14
71.00
67.32
55.51
54.86
49.20
39.53
38.35
30.37
25.39
23.25
22.71
OCH3
(S,S*)-9
f1 (ppm)

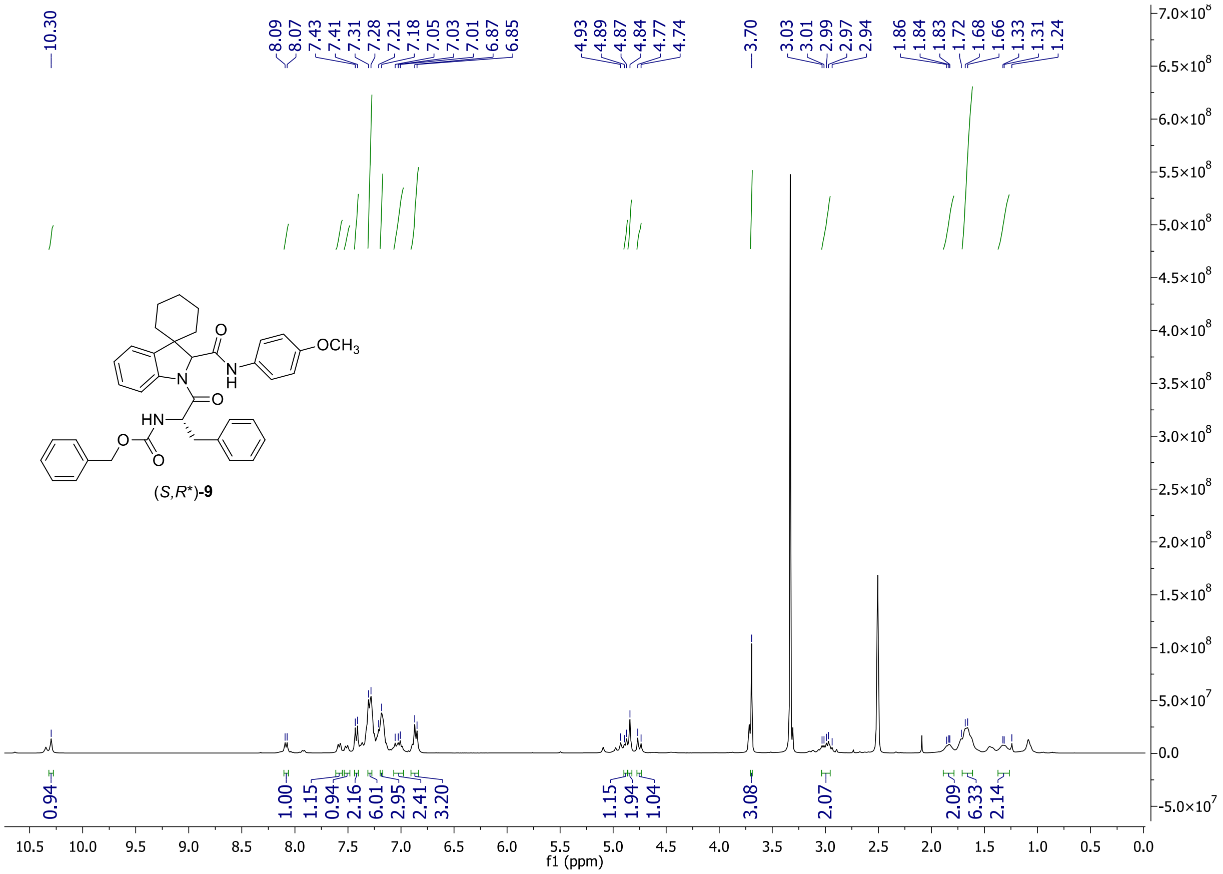
10.30
8.09
8.07
7.43
7.41
7.31
7.28
7.21
7.18
7.05
7.03
7.01
6.87
6.85
4.93
4.89
4.87
4.84
4.77
4.74
3.70
3.03
3.01
2.99
2.97
2.94
1.86
1.84
1.83
1.72
1.68
1.66
1.33
1.31
1.24
OCH3
(S,R*)-9
0.94
1.00
1.15
0.94
2.16
6.01
2.95
2.41
3.20
1.15
1.94
1.04
3.08
2.07
2.09
6.33
2.14
f1 (ppm)

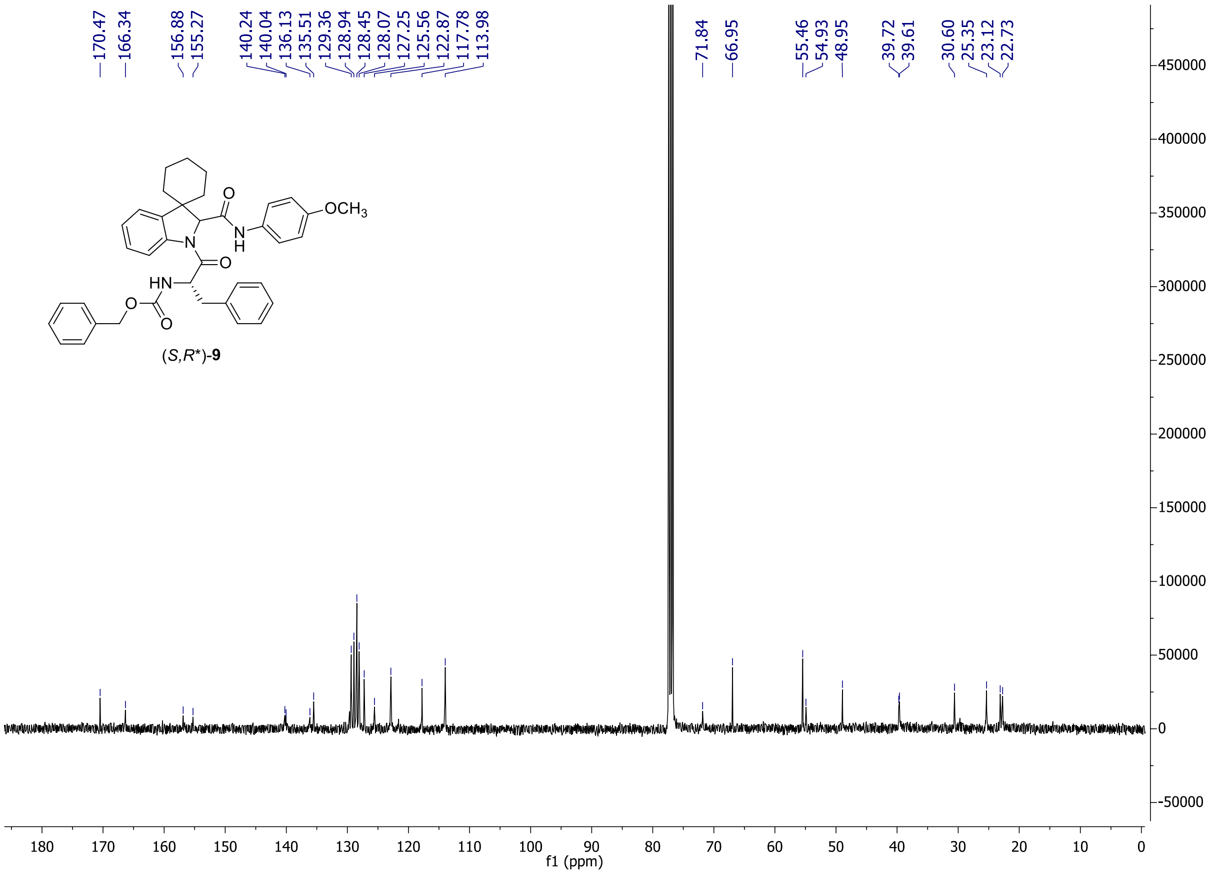
170.47
166.34
156.88
155.27
140.24
140.04
136.13
135.51
129.36
128.94
128.45
128.07
127.25
125.56
122.87
117.78
113.98
71.84
66.95
55.46
54.93
48.95
39.72
39.61
30.60
25.35
23.12
22.73
f1 (ppm)

(*S*,*R**)-**9**

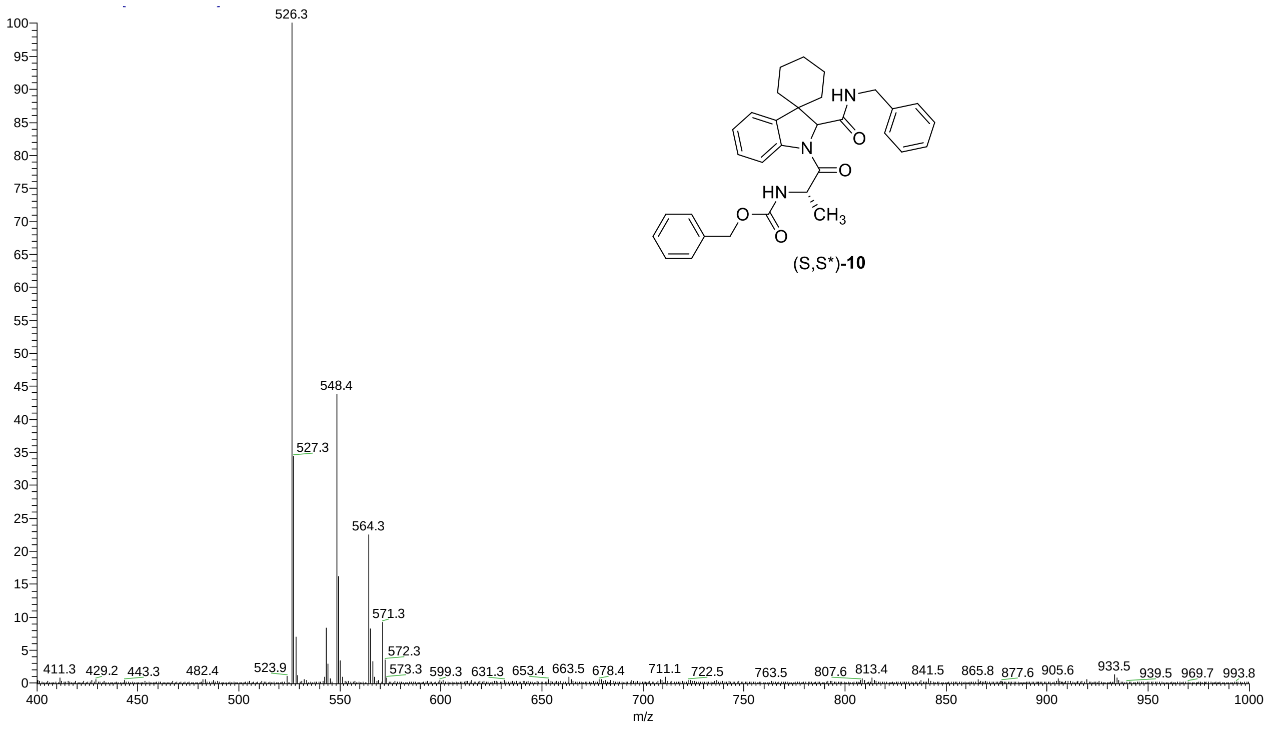
526.3
548.4
527.3
564.3
571.3
572.3
573.3
m/z

(S,S*)-**10**

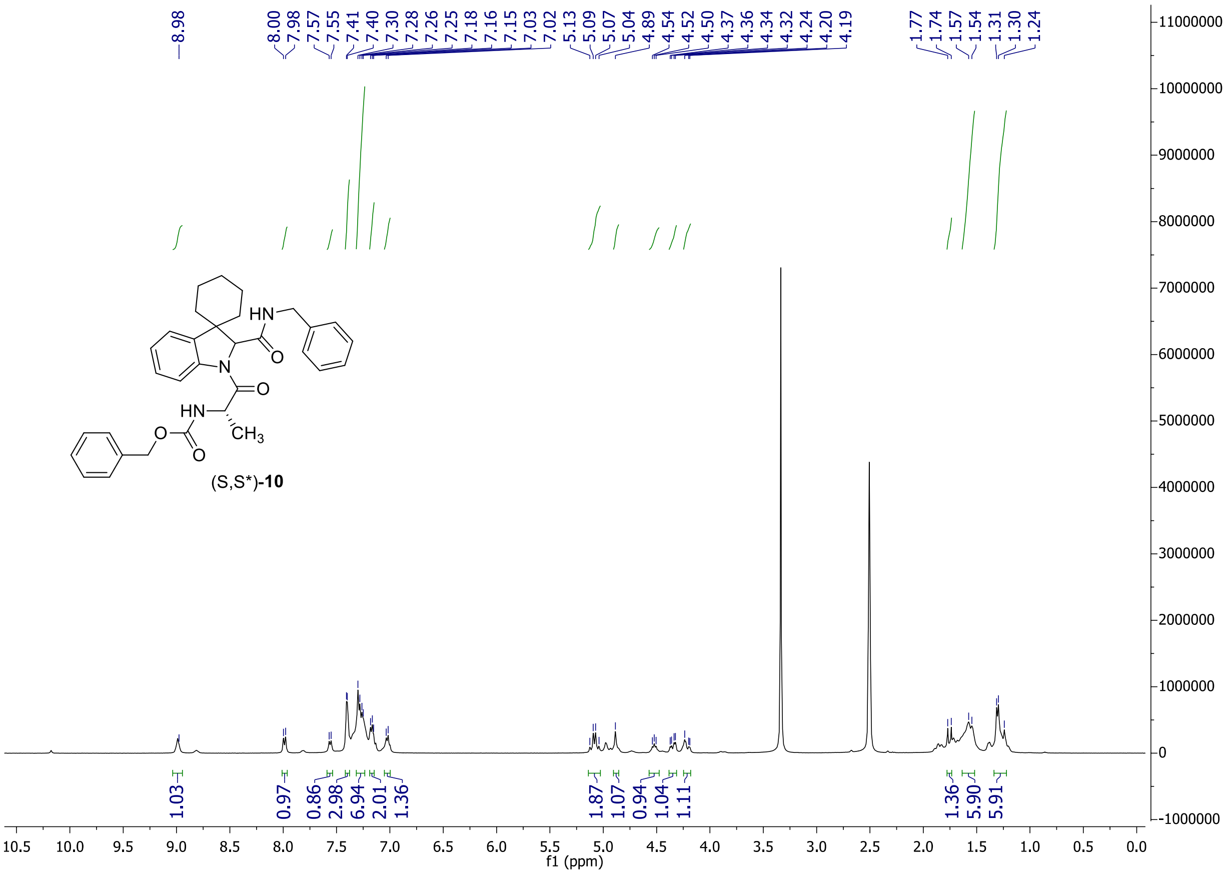
8.98
8.00 7.98 7.57 7.55 7.41 7.40 7.30 7.28 7.26 7.25 7.18 7.16 7.15 7.03 7.02
5.13 5.09 5.07 5.04 4.89 4.54 4.52 4.50 4.37 4.36 4.34 4.32 4.24 4.20 4.19
1.77 1.74 1.57 1.54 1.31 1.30 1.24
1.03 0.97 0.86 2.98 6.94 2.01 1.36 1.87 1.07 0.94 1.04 1.11 1.36 5.90 5.91
HN
O
N
O
HN
CH3
O
O
(S,S*)-10
f1 (ppm)

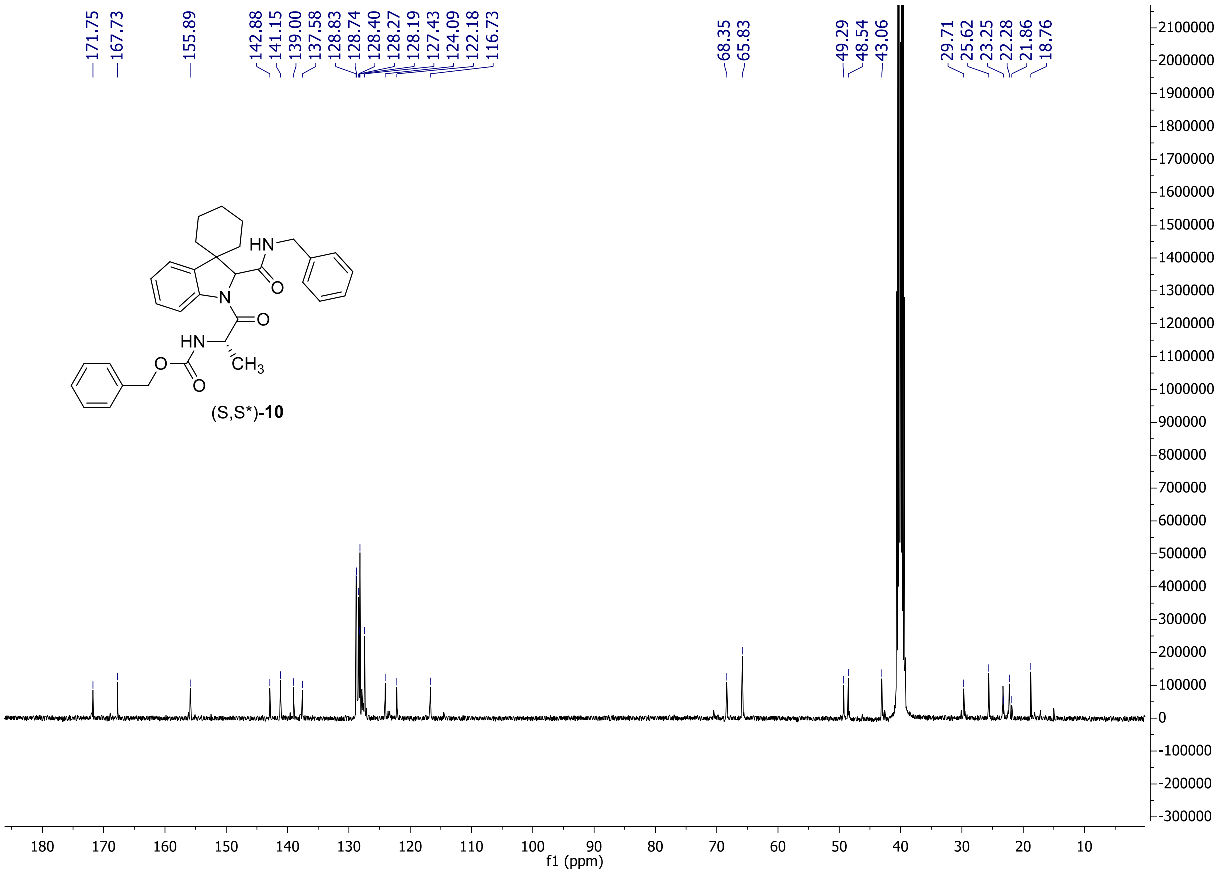
171.75 167.73 155.89
142.88 141.15 139.00 137.58 128.83 128.74 128.40 128.27 128.19 127.43 124.09 122.18 116.73
68.35 65.83
49.29 48.54 43.06
29.71 25.62 23.25 22.28 21.86 18.76
HN
O
N
O
HN
CH3
O
O
(S,S*)-10
f1 (ppm)

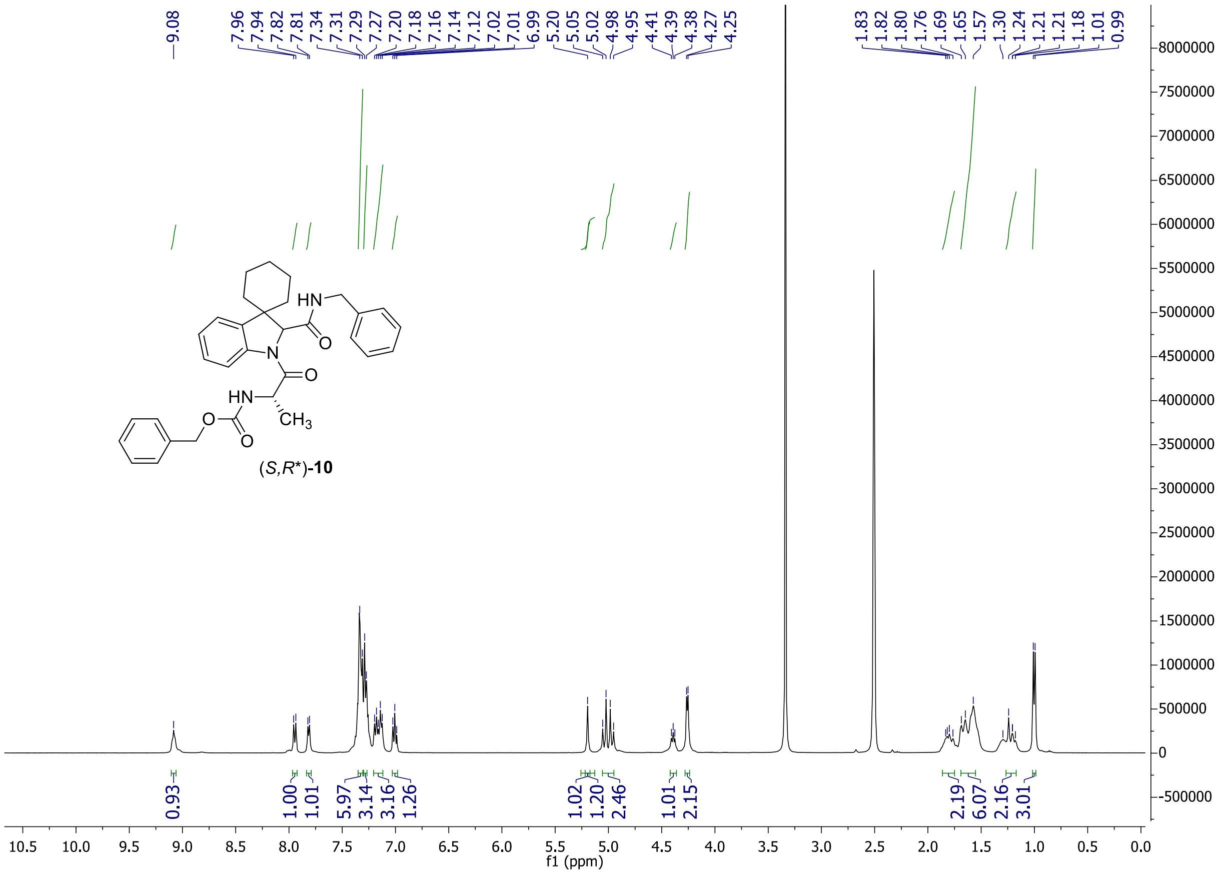
9.08
7.96 7.94 7.82 7.81 7.34 7.31 7.29 7.27 7.20 7.18 7.16 7.14 7.12 7.02 7.01 6.99
5.20 5.05 5.02 4.98 4.95 4.41 4.39 4.38 4.27 4.25
1.83 1.82 1.80 1.76 1.69 1.65 1.57 1.30 1.24 1.21 1.21 1.18 1.01 0.99
0.93 1.00 1.01 5.97 3.14 3.16 1.26 1.02 1.20 2.46 1.01 2.15 2.19 6.07 2.16 3.01
HN
O
N
HN
O
CH3
O
O
(S,R*)-10
f1 (ppm)

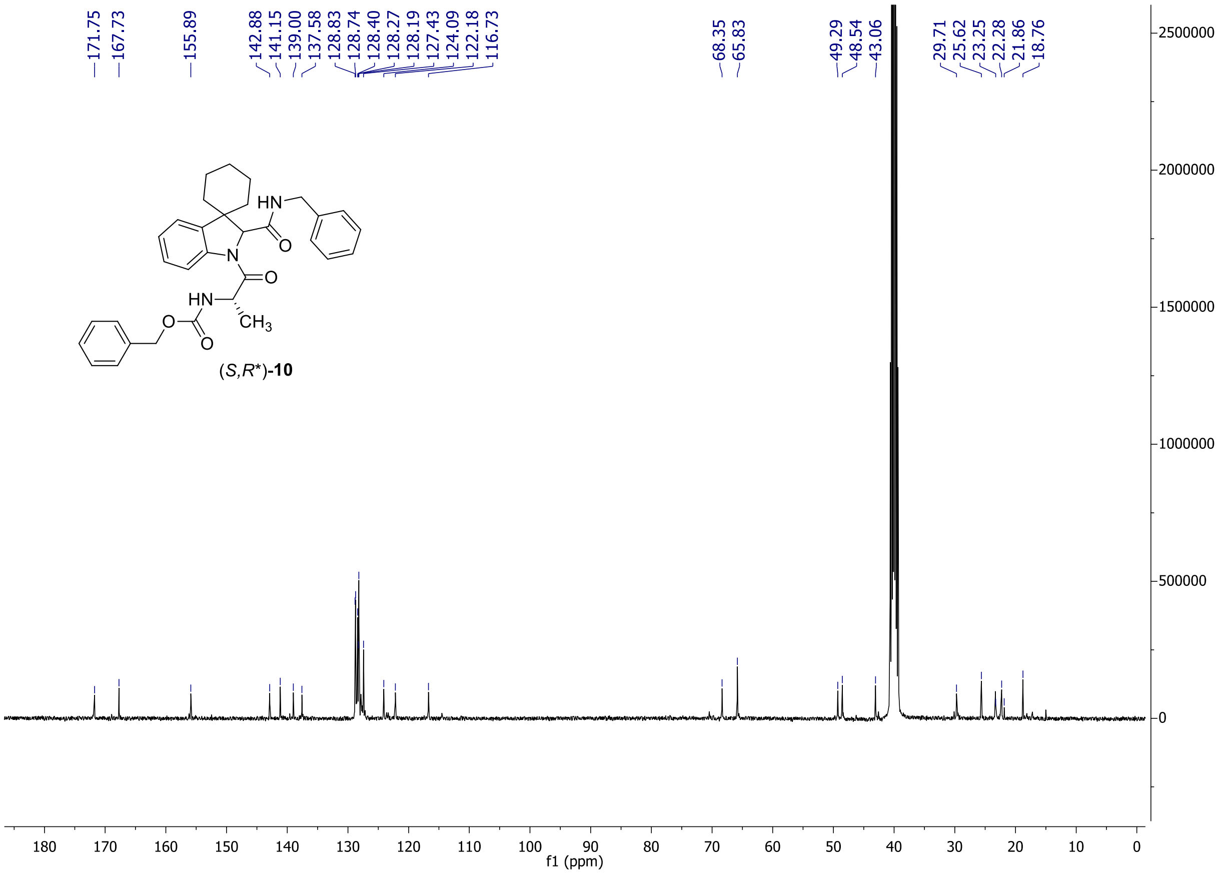
171.75 167.73 155.89
142.88 141.15 139.00 137.58 128.83 128.74 128.40 128.27 128.19 127.43 124.09 122.18 116.73
68.35 65.83
49.29 48.54 43.06
29.71 25.62 23.25 22.28 21.86 18.76
HN
O
N
HN
O
CH3
O
O
(S,R*)-10
f1 (ppm)

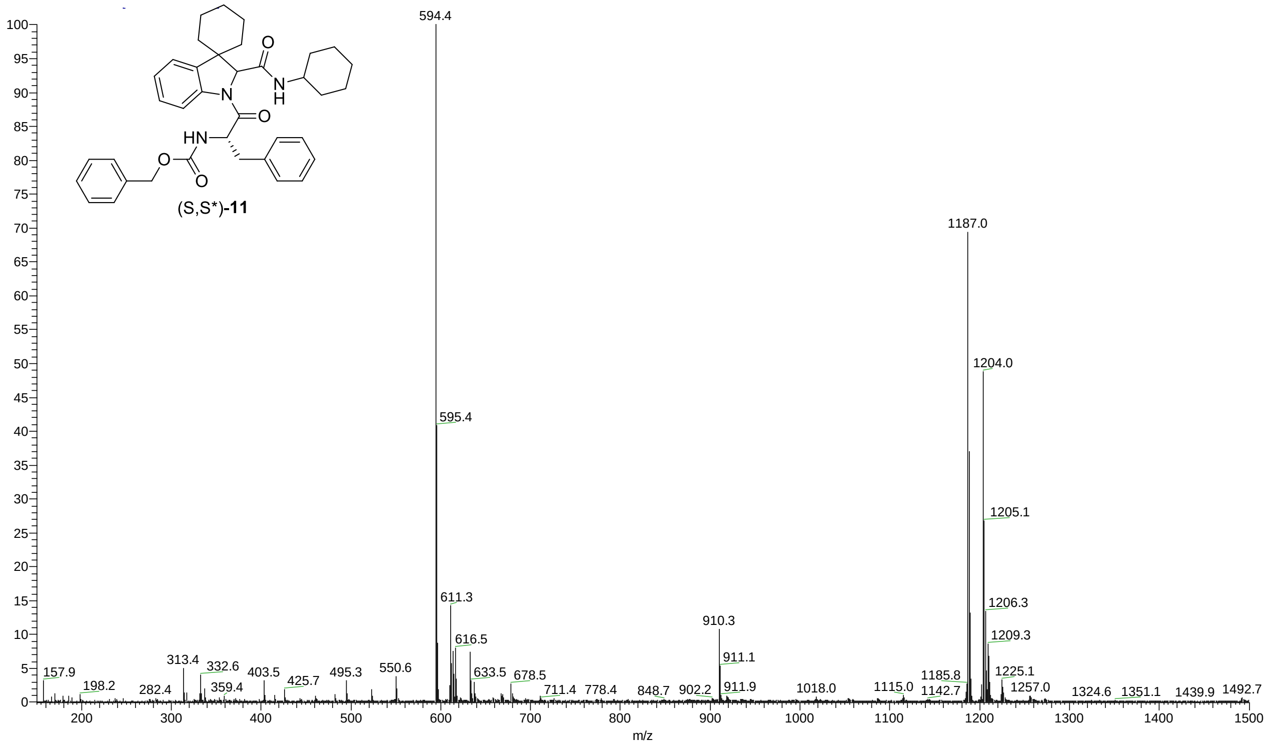
(S,S*)-11
594.4
595.4
611.3
616.5
633.5
678.5
910.3
911.1
1187.0
1204.0
1205.1
1206.3
1209.3
1225.1
m/z

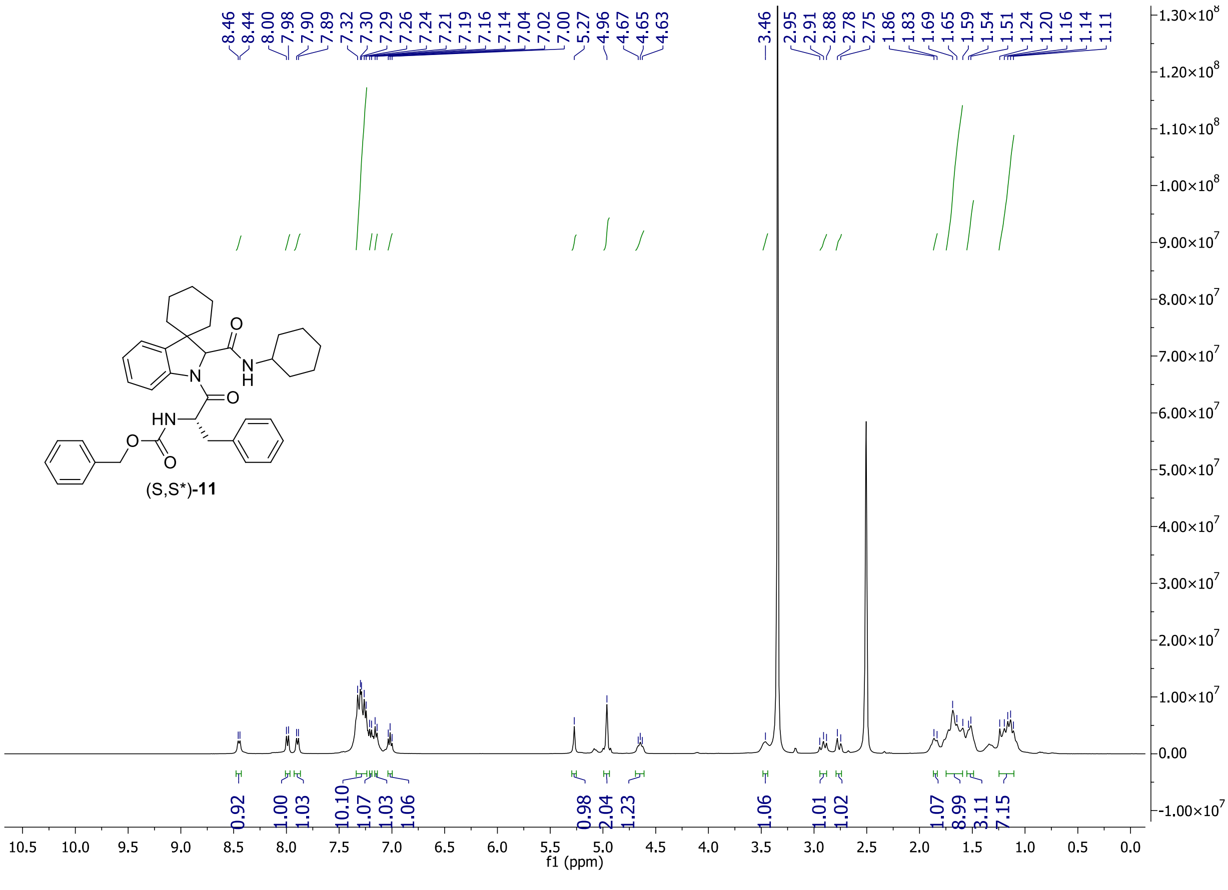
(S,S*)-11
f1 (ppm)

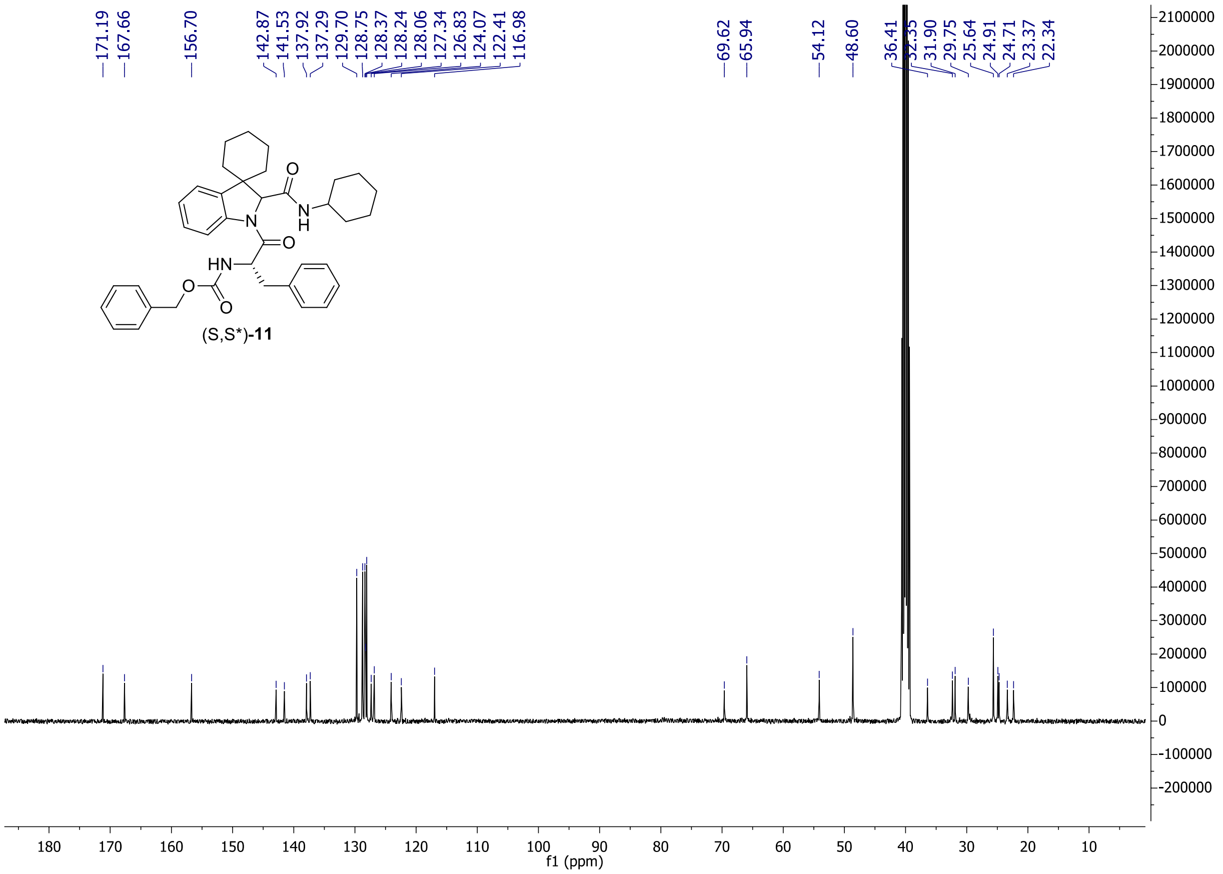
171.19
167.66
156.70
142.87
141.53
137.92
137.29
129.70
128.75
128.37
128.24
128.06
127.34
126.83
124.07
122.41
116.98
69.62
65.94
54.12
48.60
36.41
32.35
31.90
29.75
25.64
24.91
24.71
23.37
22.34
(S,S*)-11
f1 (ppm)

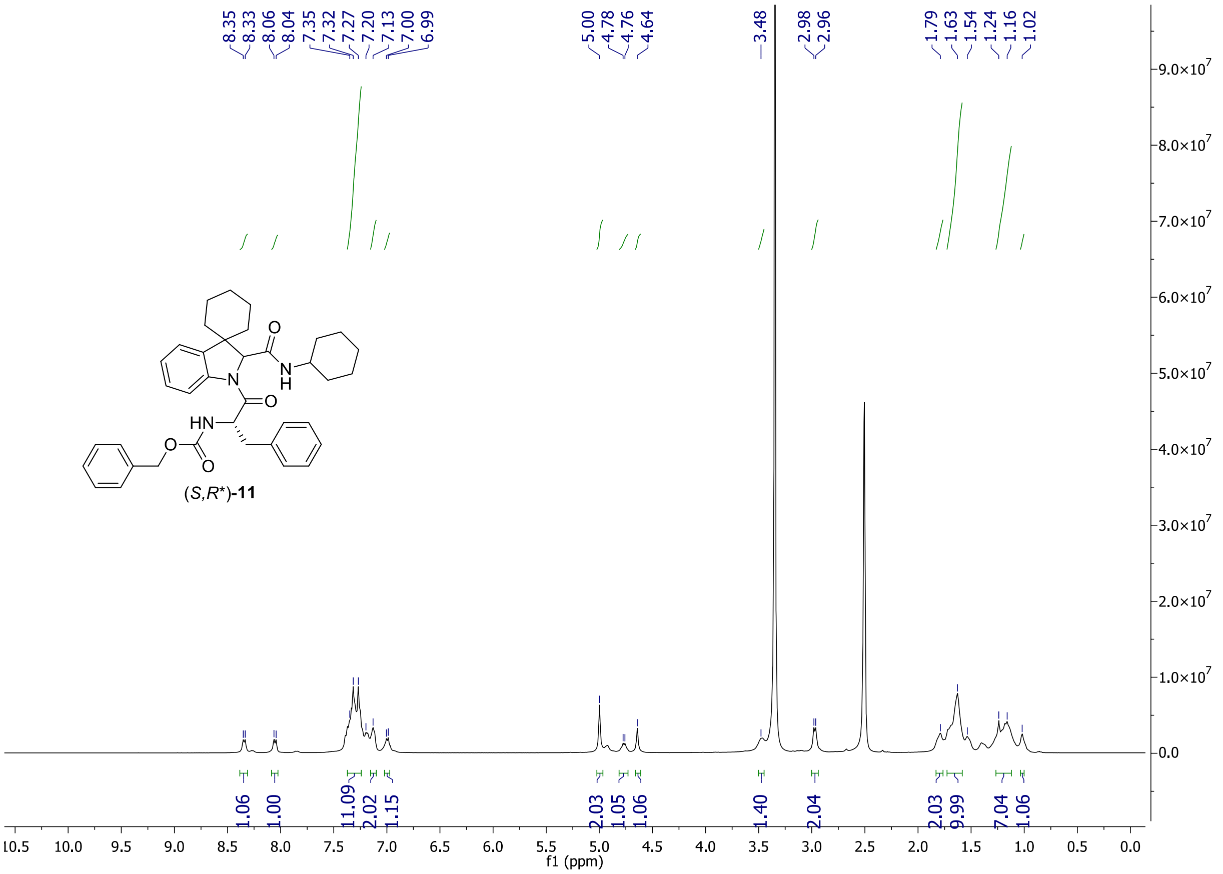
8.35
8.33
8.06
8.04
7.35
7.32
7.27
7.20
7.13
7.00
6.99
5.00
4.78
4.76
4.64
3.48
2.98
2.96
1.79
1.63
1.54
1.24
1.16
1.02
(S,R*)-11
1.06
1.00
11.09
2.02
1.15
2.03
1.05
1.06
1.40
2.04
2.03
9.99
7.04
1.06
f1 (ppm)

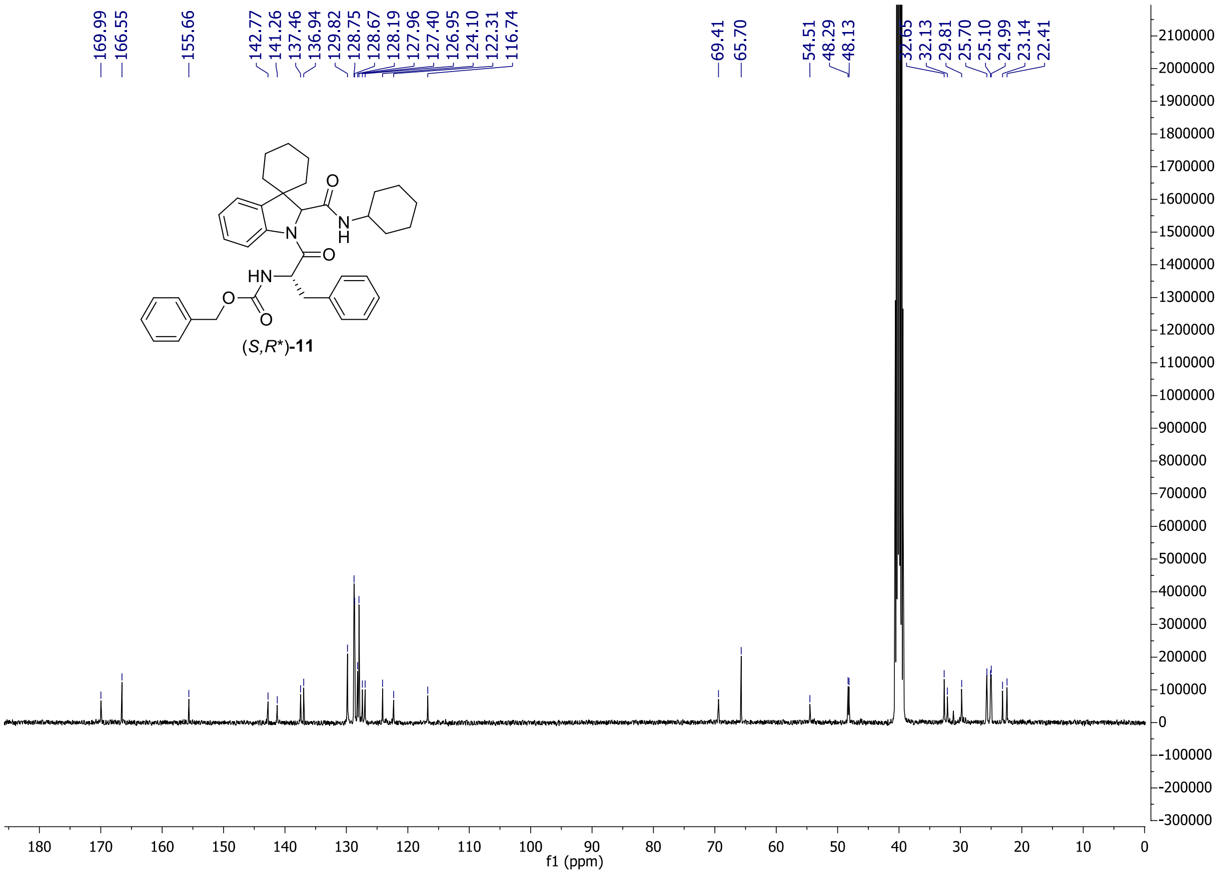
169.99
166.55
155.66
142.77
141.26
137.46
136.94
129.82
128.75
128.67
128.19
127.96
127.40
126.95
124.10
122.31
116.74
69.41
65.70
54.51
48.29
48.13
32.65
32.13
29.81
25.70
25.10
24.99
23.14
22.41
(S,R*)-11
f1 (ppm)

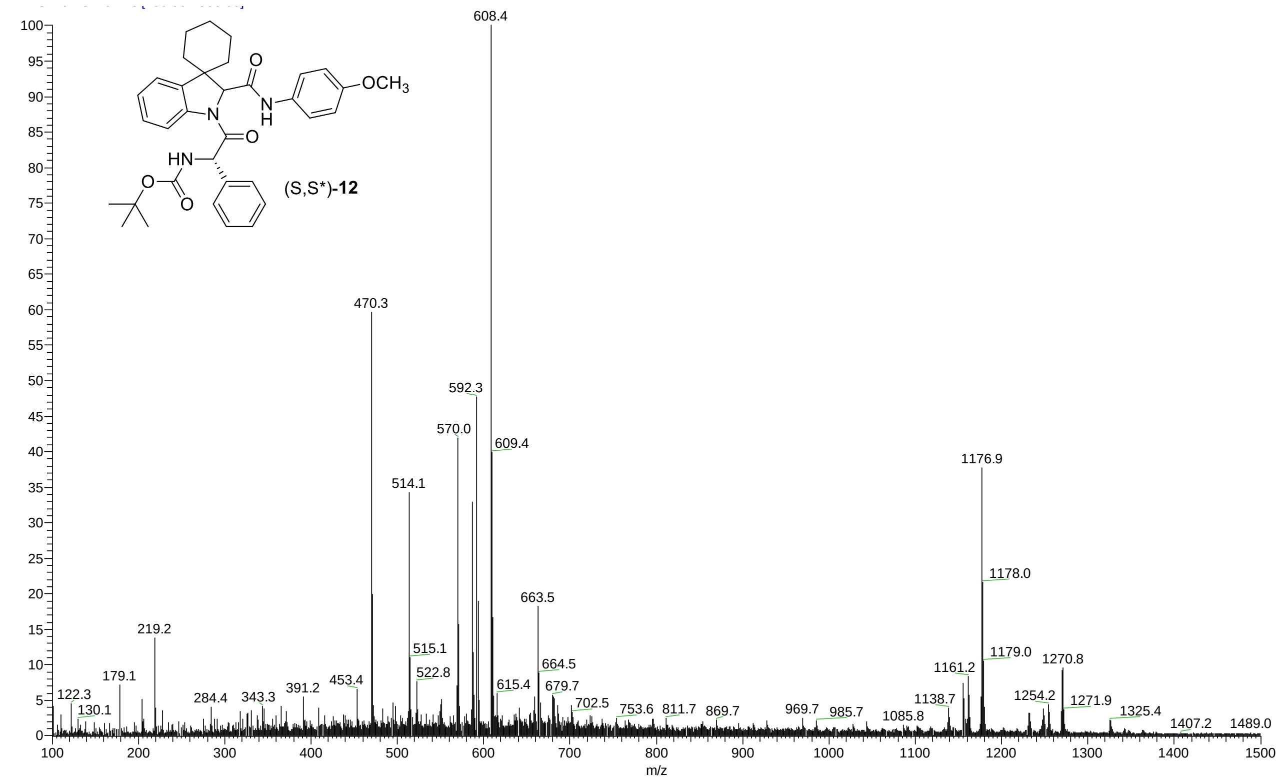
OCH3
(S,S*)-12
608.4
470.3
592.3
570.0
609.4
514.1
1176.9
1178.0
663.5
219.2
515.1
522.8
664.5
1179.0
1270.8
1161.2
179.1
122.3
130.1
284.4
343.3
391.2
453.4
615.4
679.7
702.5
753.6
811.7
869.7
969.7
985.7
1085.8
1138.7
1254.2
1271.9
1325.4
1407.2
1489.0
m/z

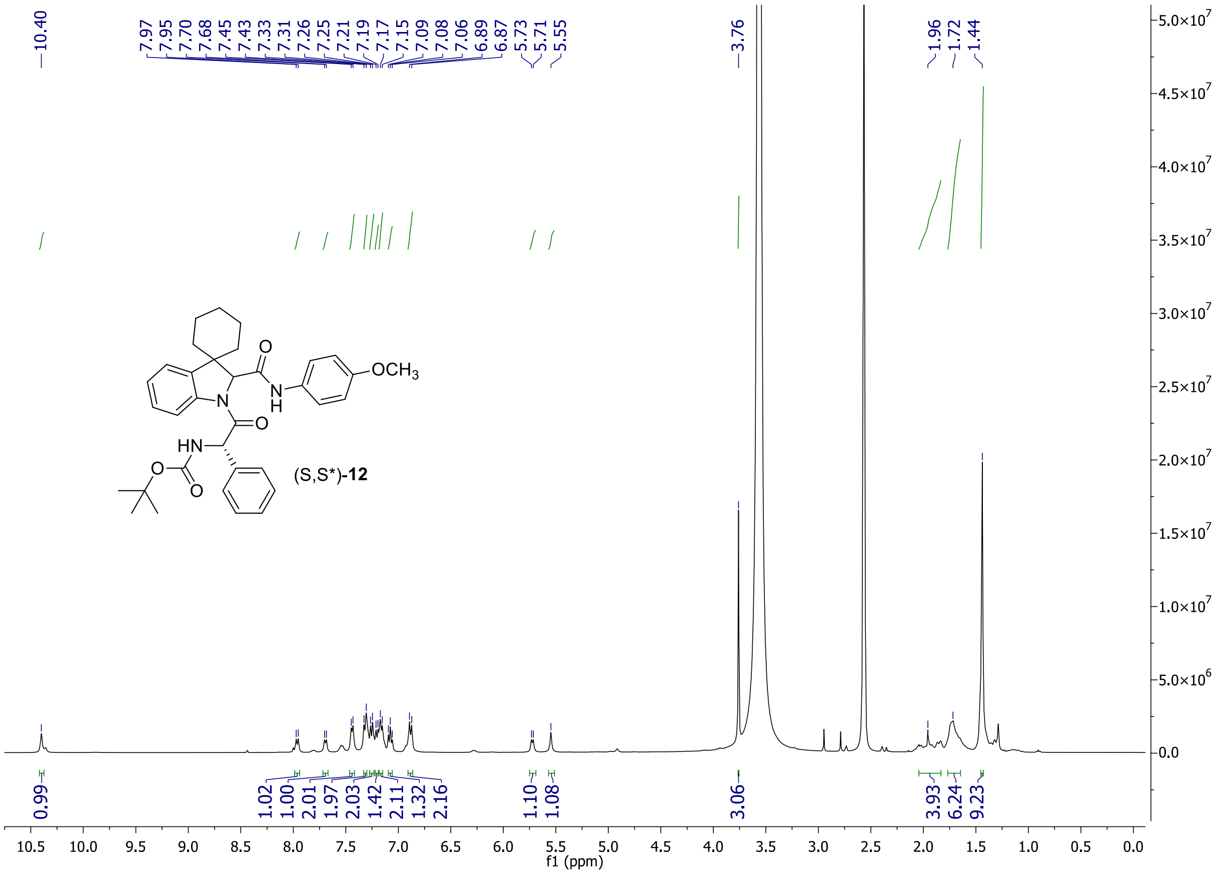

10.40
7.97
7.95
7.70
7.68
7.45
7.43
7.33
7.31
7.26
7.25
7.21
7.19
7.17
7.15
7.09
7.08
7.06
6.89
6.87
5.73
5.71
5.55
3.76
1.96
1.72
1.44
OCH3
O
N
H
N
O
HN
O
O
(S,S*)-12
0.99
1.02
1.00
2.01
1.97
2.03
1.42
2.11
1.32
2.16
1.10
1.08
3.06
3.93
6.24
9.23
f1 (ppm)

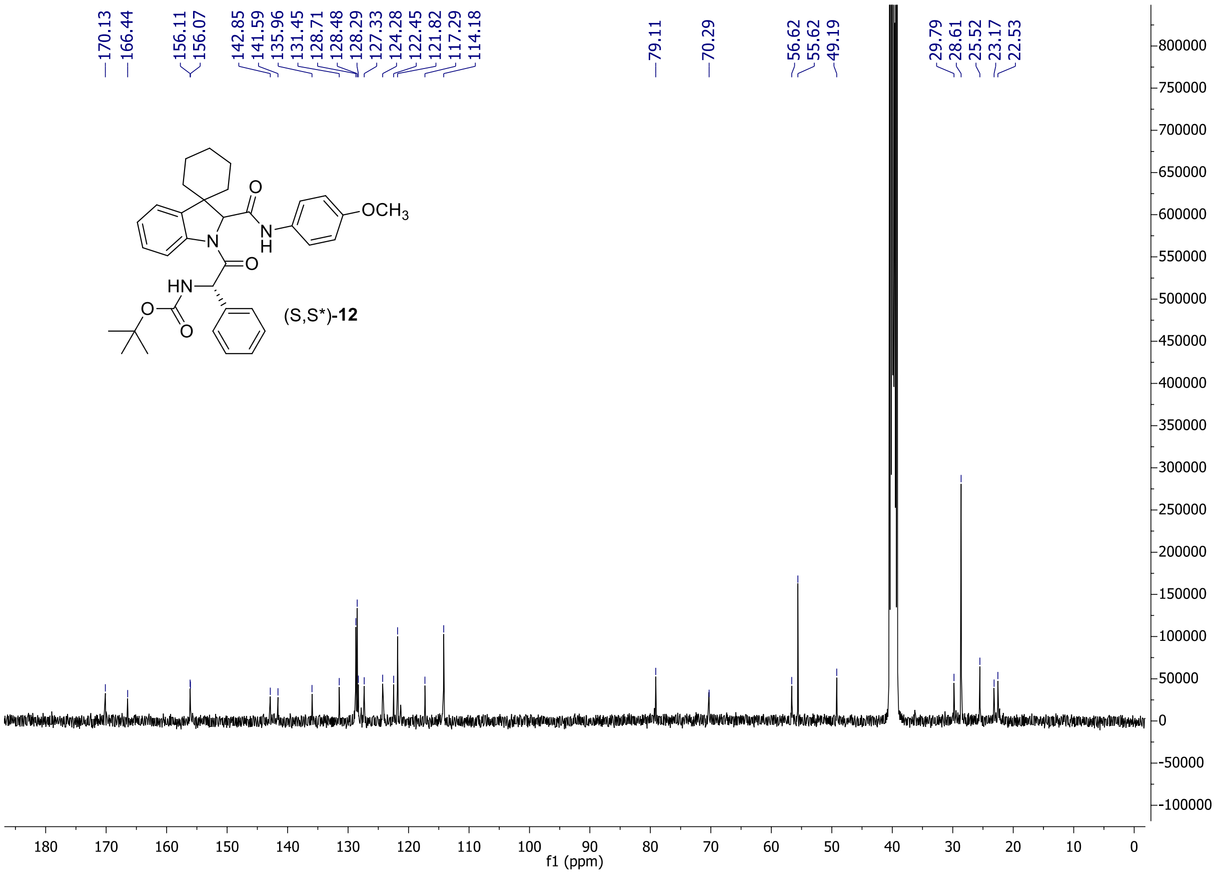

170.13
166.44
156.11
156.07
142.85
141.59
135.96
131.45
128.71
128.48
128.29
127.33
124.28
122.45
121.82
117.29
114.18
79.11
70.29
56.62
55.62
49.19
29.79
28.61
25.52
23.17
22.53
OCH3
(S,S*)-12
f1 (ppm)

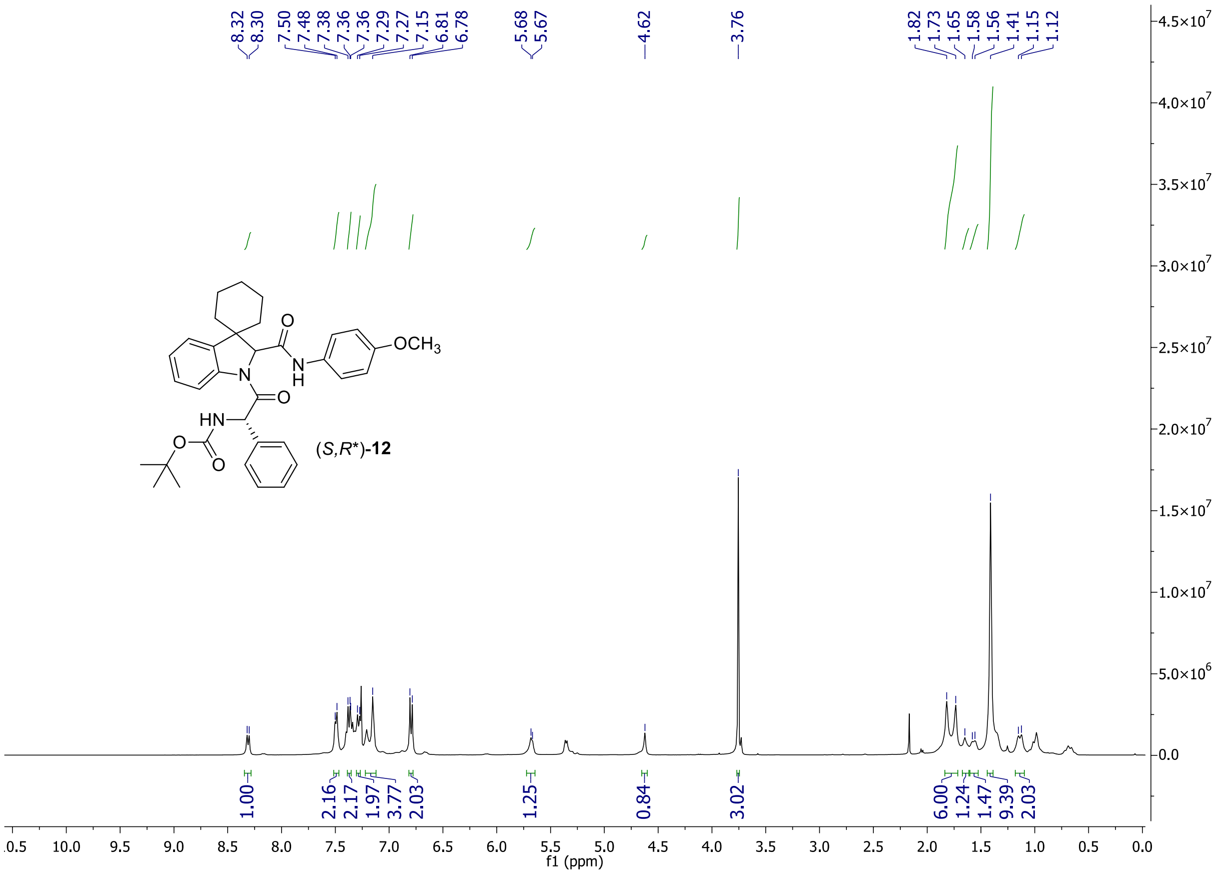
8.32
8.30
7.50
7.48
7.38
7.36
7.36
7.29
7.27
7.15
6.81
6.78
5.68
5.67
4.62
3.76
1.82
1.73
1.65
1.58
1.56
1.41
1.15
1.12
OCH3
HN
O
N
H
(S,R*)-12
1.00
2.16
2.17
1.97
3.77
2.03
1.25
0.84
3.02
6.00
1.24
1.47
9.39
2.03
f1 (ppm)

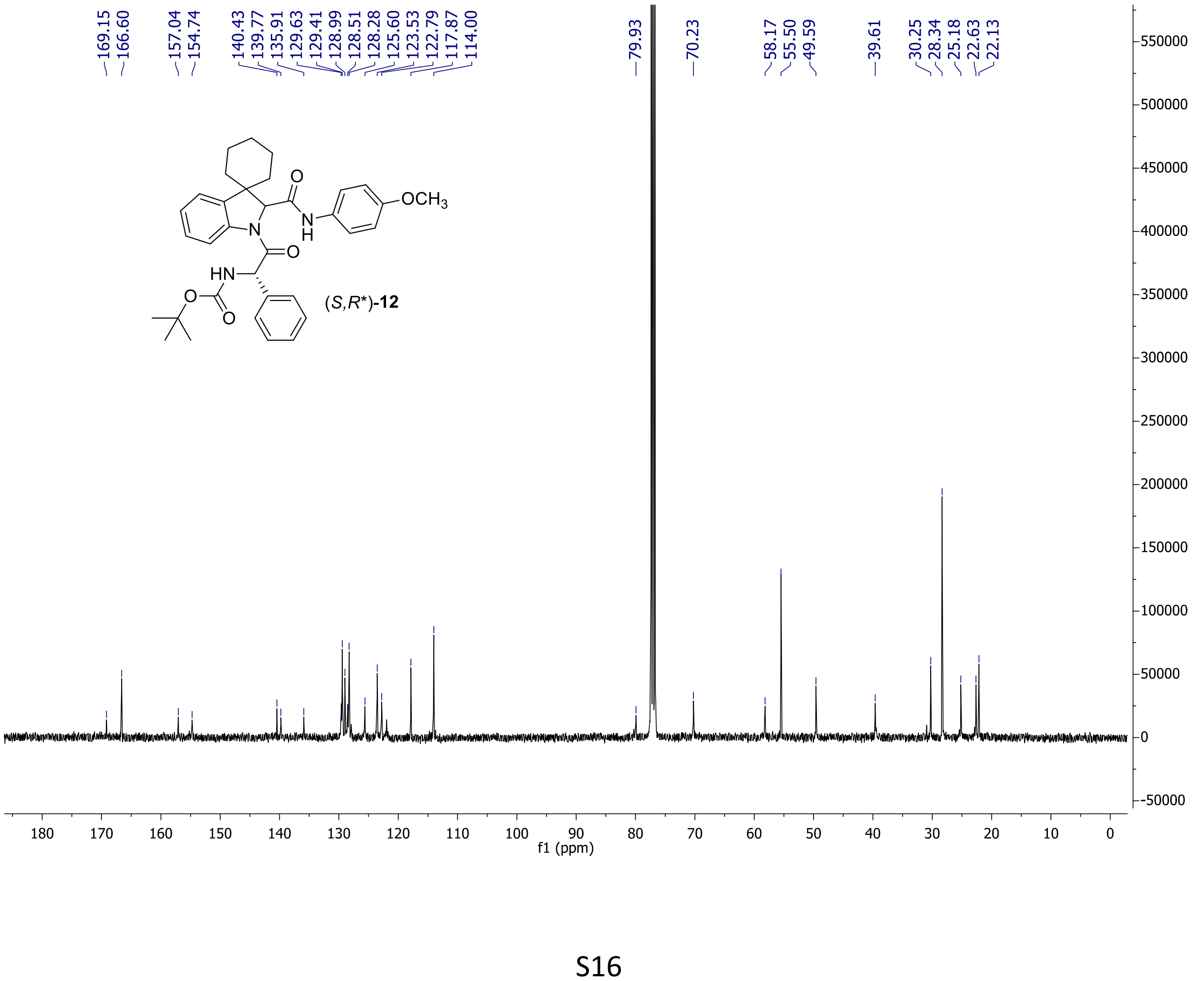
169.15
166.60
157.04
154.74
140.43
139.77
135.91
129.63
129.41
128.99
128.51
128.28
125.60
123.53
122.79
117.87
114.00
79.93
70.23
58.17
55.50
49.59
39.61
30.25
28.34
25.18
22.63
22.13
OCH3
HN
O
N
H
(S,R*)-12
f1 (ppm)

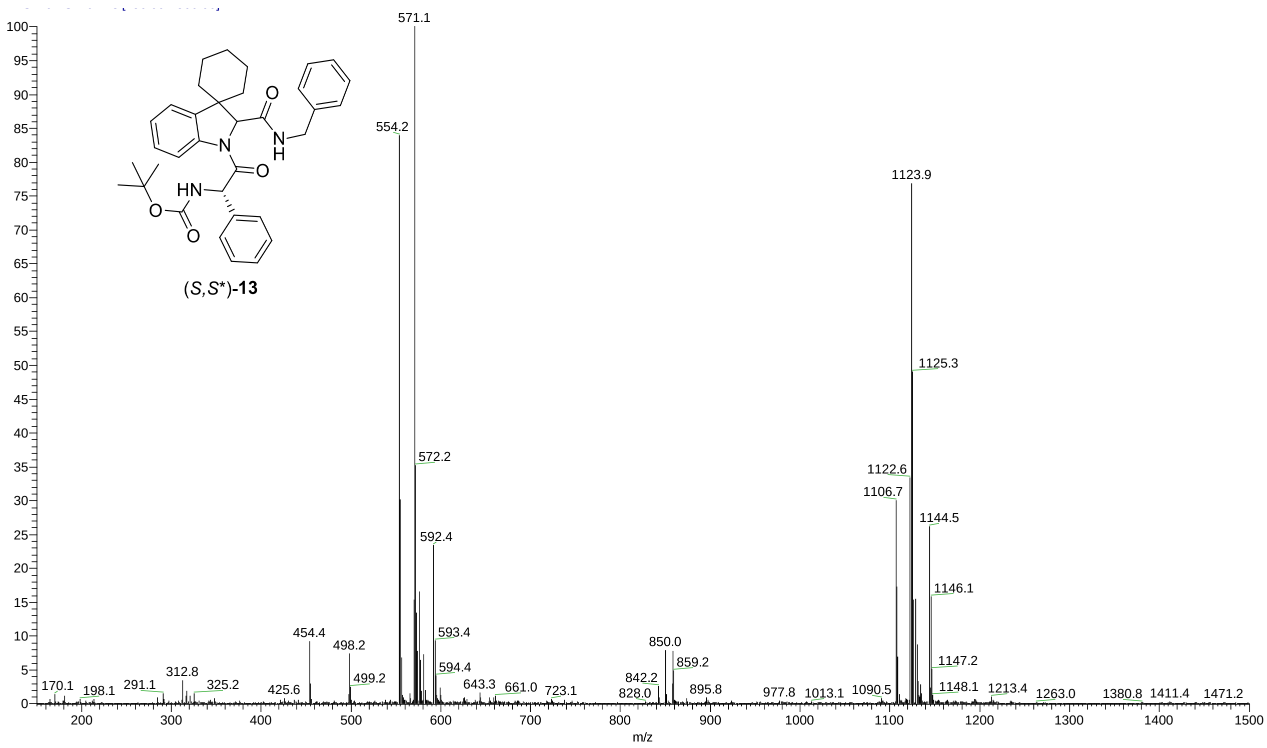
571.1
554.2
1123.9
1125.3
572.2
1122.6
1106.7
592.4
1144.5
1146.1
593.4
454.4
498.2
850.0
859.2
1147.2
594.4
499.2
842.2
828.0
895.8
643.3
661.0
723.1
170.1
198.1
291.1
312.8
325.2
425.6
977.8
1013.1
1090.5
1148.1
1213.4
1263.0
1380.8
1411.4
1471.2
m/z
HN
O
N
H
(S,S*)-13

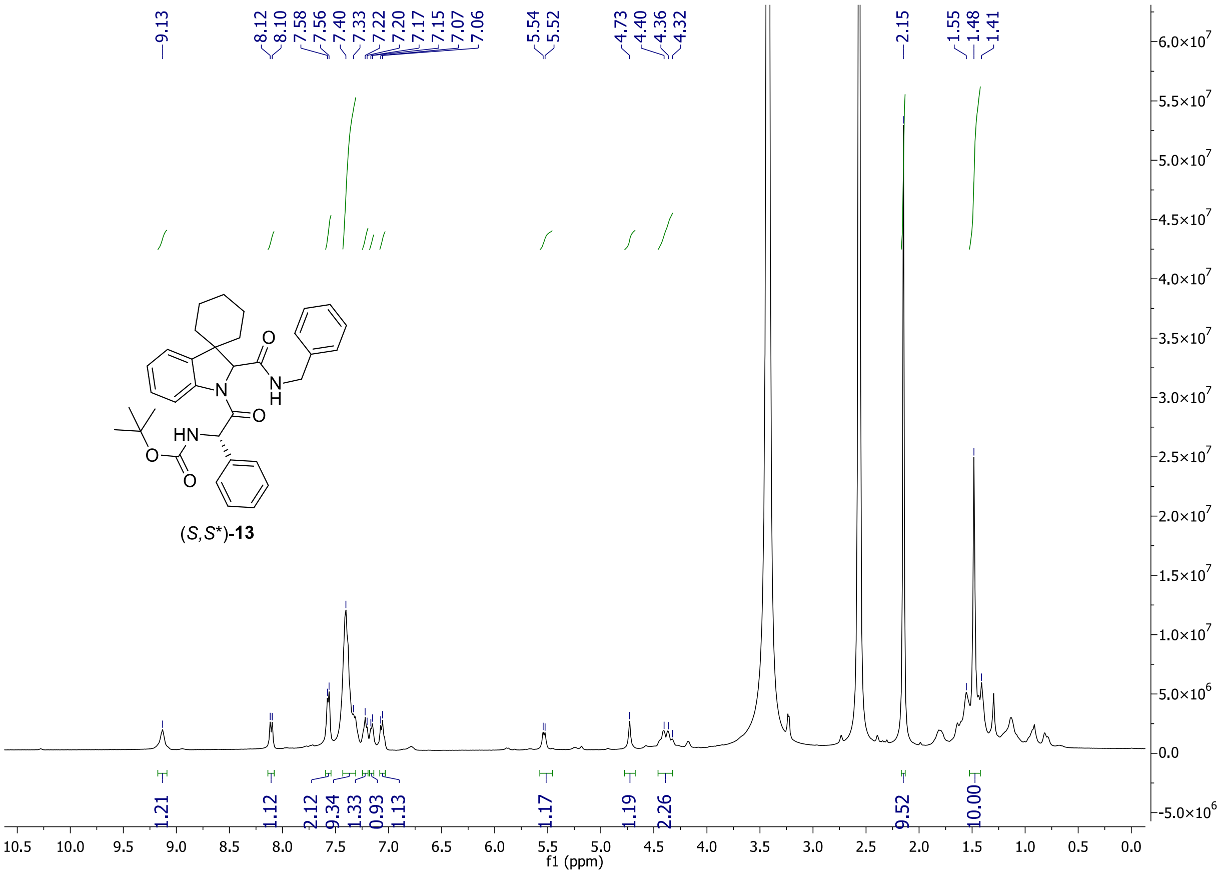
9.13
8.12
8.10
7.58
7.56
7.40
7.33
7.22
7.20
7.17
7.15
7.07
7.06
5.54
5.52
4.73
4.40
4.36
4.32
2.15
1.55
1.48
1.41
1.21
1.12
2.12
9.34
1.33
0.93
1.13
1.17
1.19
2.26
9.52
10.00
f1 (ppm)
(S,S*)-13

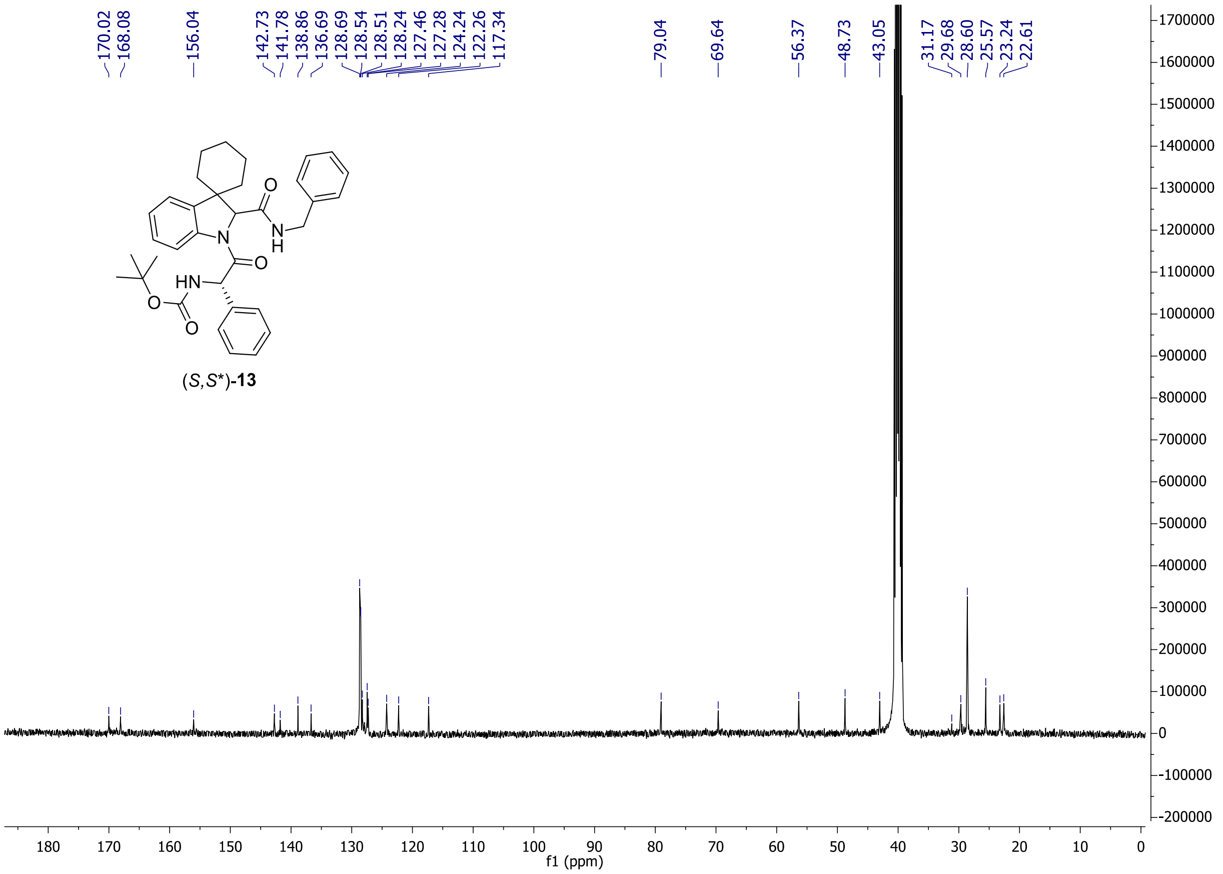
170.02
168.08
156.04
142.73
141.78
138.86
136.69
128.69
128.54
128.51
128.24
127.46
127.28
124.24
122.26
117.34
79.04
69.64
56.37
48.73
43.05
31.17
29.68
28.60
25.57
23.24
22.61
(S,S*)-13
f1 (ppm)

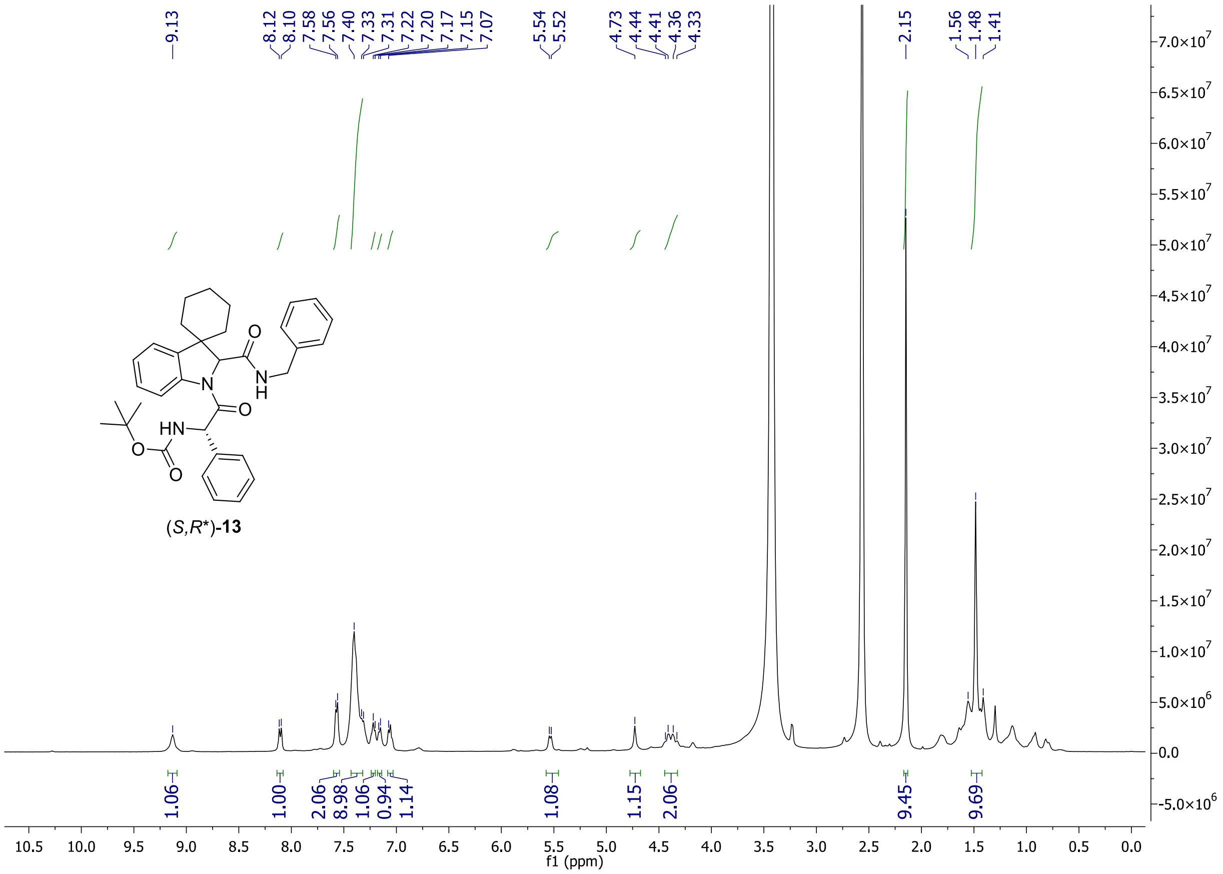
9.13
8.12
8.10
7.58
7.56
7.40
7.33
7.31
7.22
7.20
7.17
7.15
7.07
5.54
5.52
4.73
4.44
4.41
4.36
4.33
2.15
1.56
1.48
1.41
1.06
1.00
2.06
8.98
1.06
0.94
1.14
1.08
1.15
2.06
9.45
9.69
(S,R*)-13
f1 (ppm)

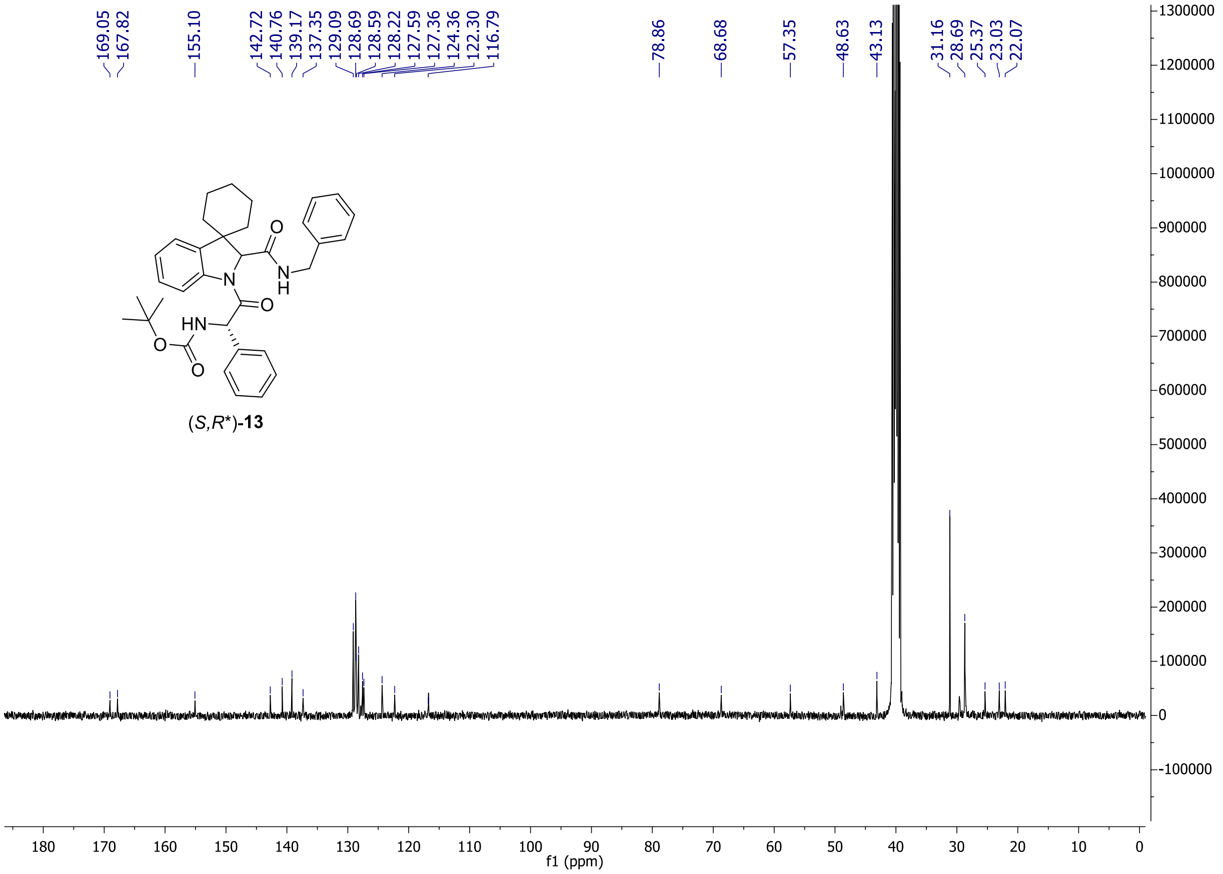

169.05
167.82
155.10
142.72
140.76
139.17
137.35
129.09
128.69
128.59
128.22
127.59
127.36
124.36
122.30
116.79
78.86
68.68
57.35
48.63
43.13
31.16
28.69
25.37
23.03
22.07
f1 (ppm)

(*S*,*R**)-**13**

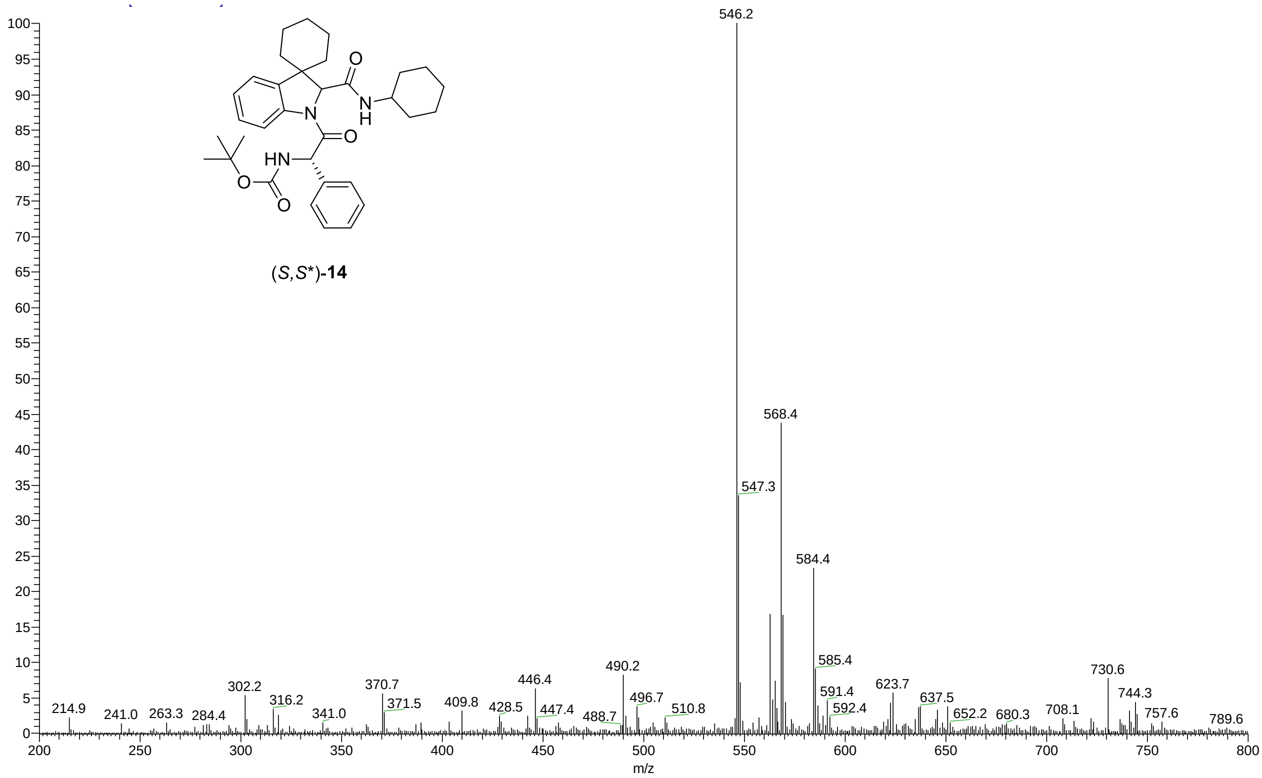

546.2
568.4
547.3
584.4
585.4
591.4
592.4
623.7
637.5
652.2
680.3
708.1
730.6
744.3
757.6
789.6
490.2
496.7
510.8
446.4
447.4
488.7
428.5
409.8
370.7
371.5
341.0
302.2
316.2
284.4
263.3
241.0
214.9
m/z

(*S*,*S**)-**14**

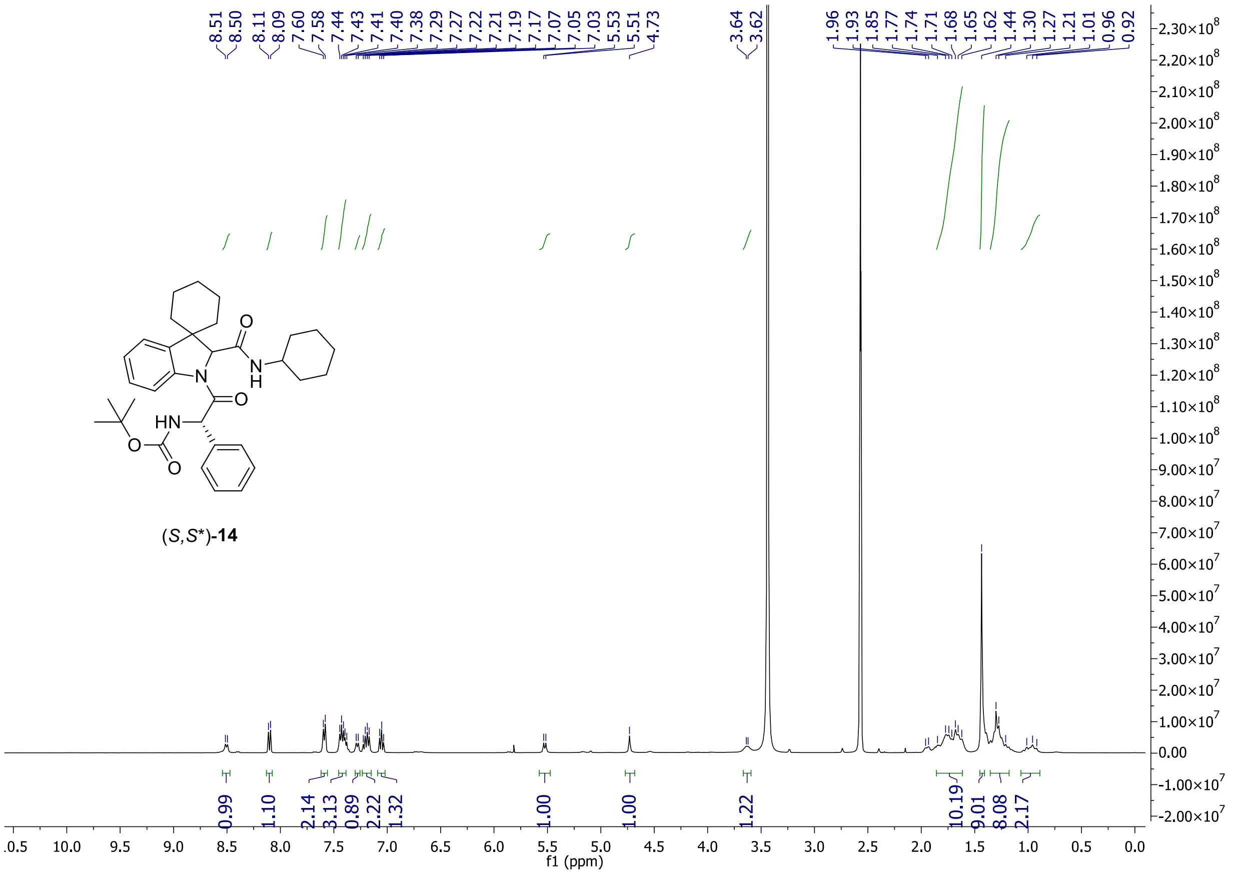
8.51 8.50 8.11 8.09 7.60 7.58 7.44 7.43 7.41 7.40 7.38 7.29 7.27 7.22 7.21 7.19 7.17 7.07 7.05 7.03 5.53 5.51 4.73
3.64 3.62
1.96 1.93 1.85 1.77 1.74 1.71 1.68 1.65 1.62 1.44 1.30 1.27 1.21 1.01 0.96 0.92
0.99 1.10 2.14 3.13 0.89 2.22 1.32 1.00 1.00 1.22 10.19 9.01 8.08 2.17
O
N
H
N
O
HN
O
O
(S,S*)-14
f1 (ppm)

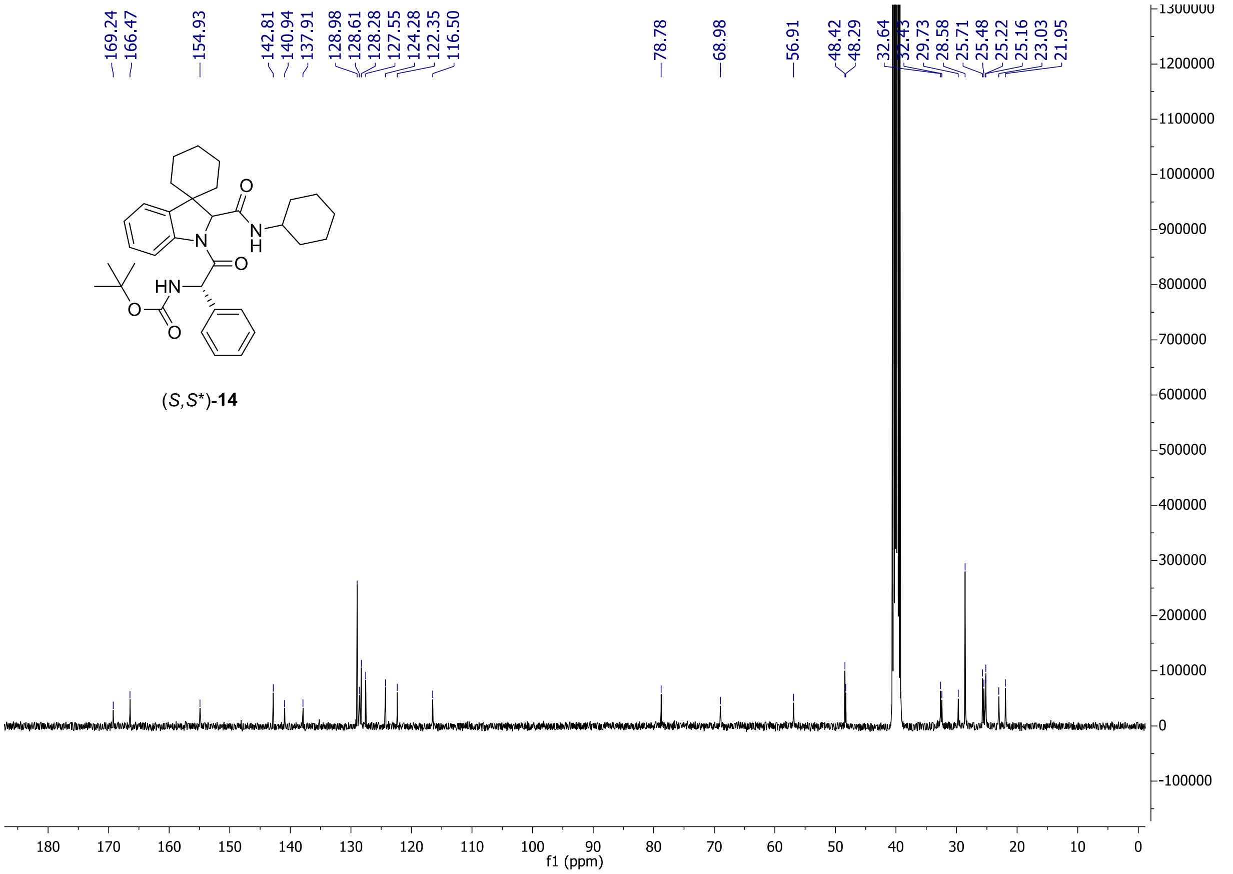
169.24 166.47 154.93 142.81 140.94 137.91 128.98 128.61 128.28 127.55 124.28 122.35 116.50
78.78 68.98 56.91 48.42 48.29 32.64 32.43 29.73 28.58 25.71 25.48 25.22 25.16 23.03 21.95
O
N
H
N
O
HN
O
O
(S,S*)-14
f1 (ppm)

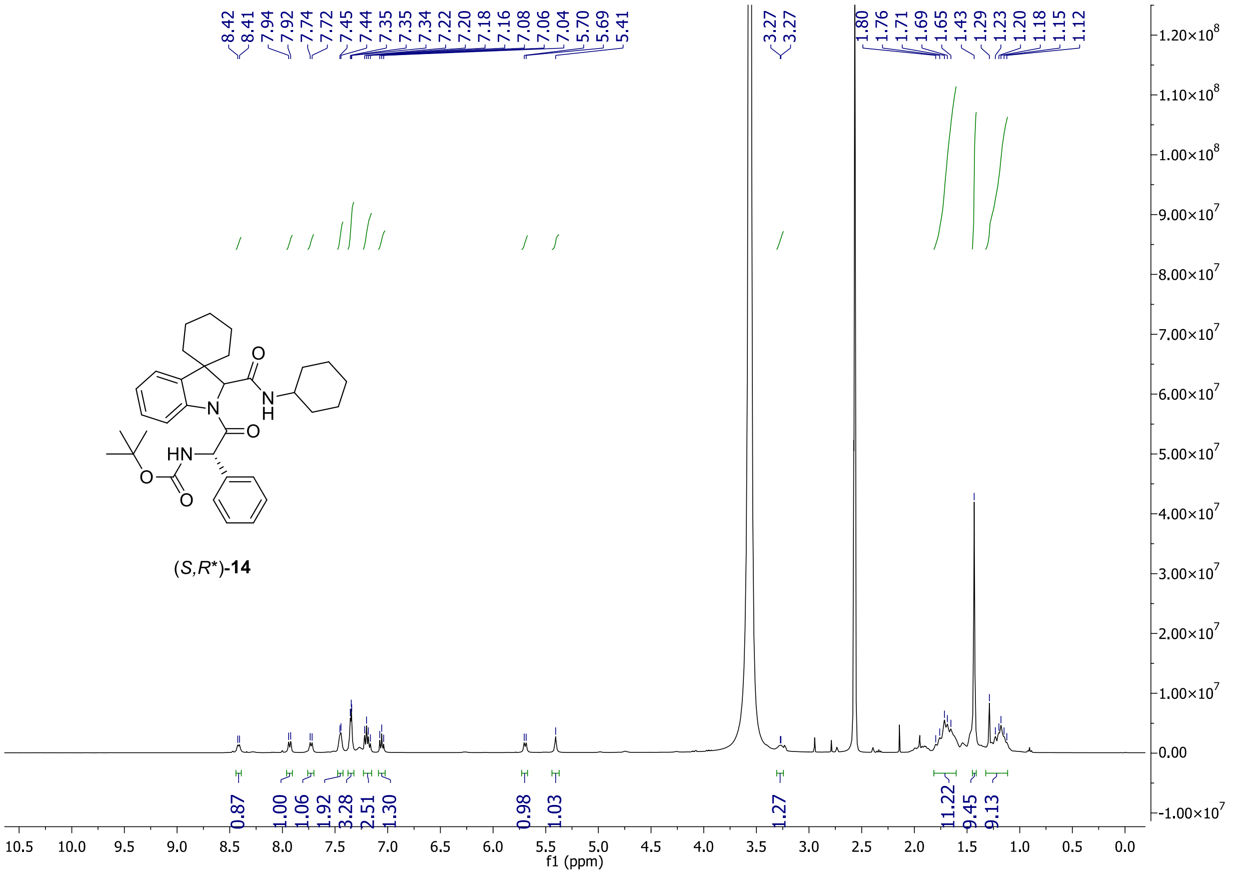

(S,R*)-14
f1 (ppm)

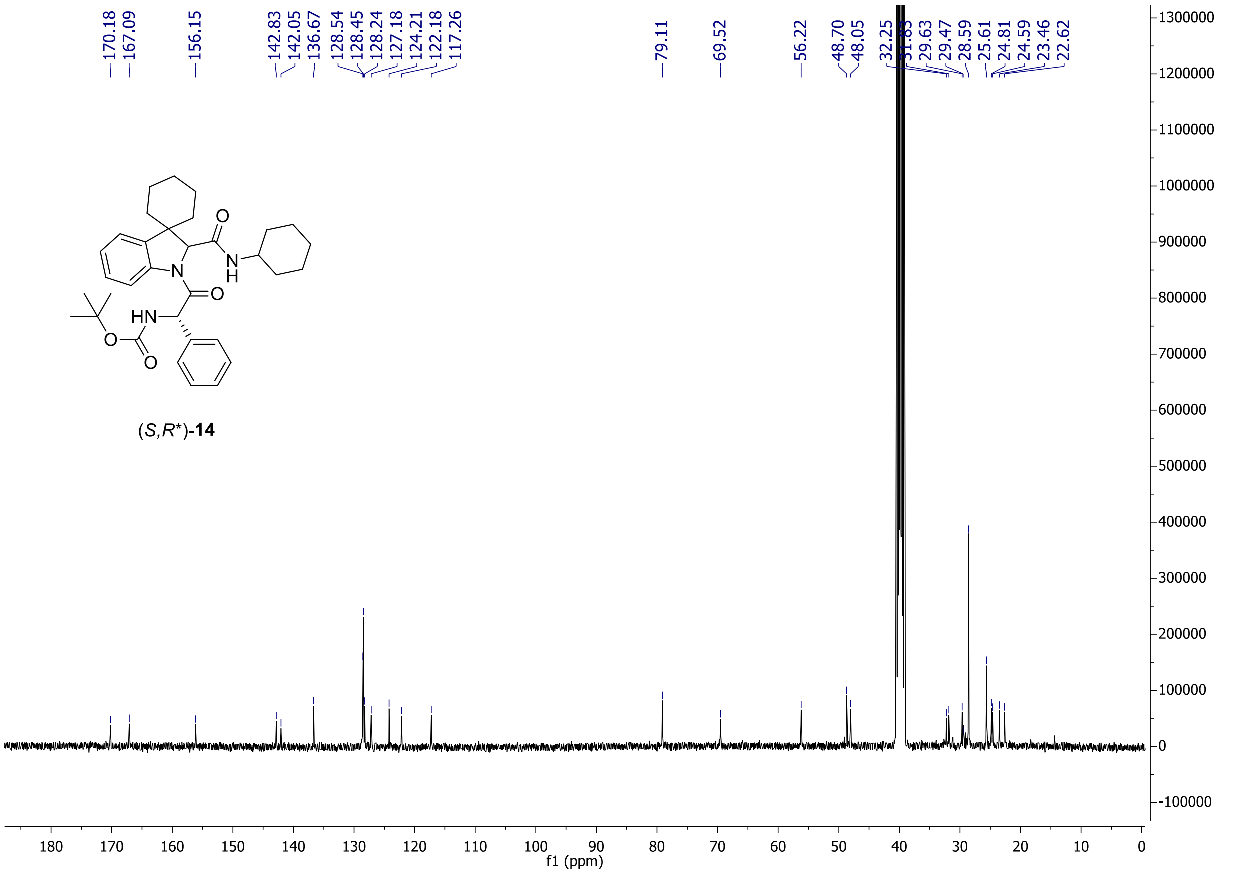

(S,R*)-14
f1 (ppm)

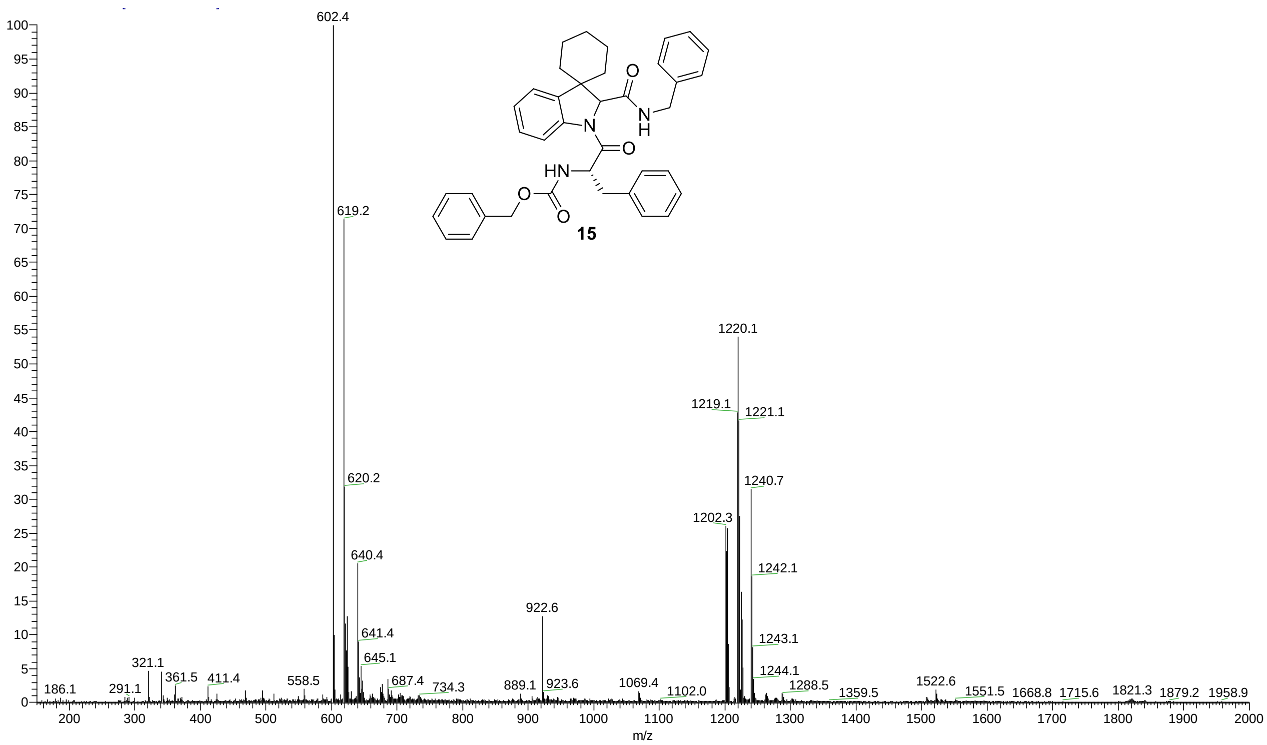
602.4
619.2
620.2
640.4
641.4
645.1
687.4
734.3
922.6
923.6
1220.1
1219.1
1221.1
1240.7
1202.3
1242.1
1243.1
1244.1
1288.5
m/z
15

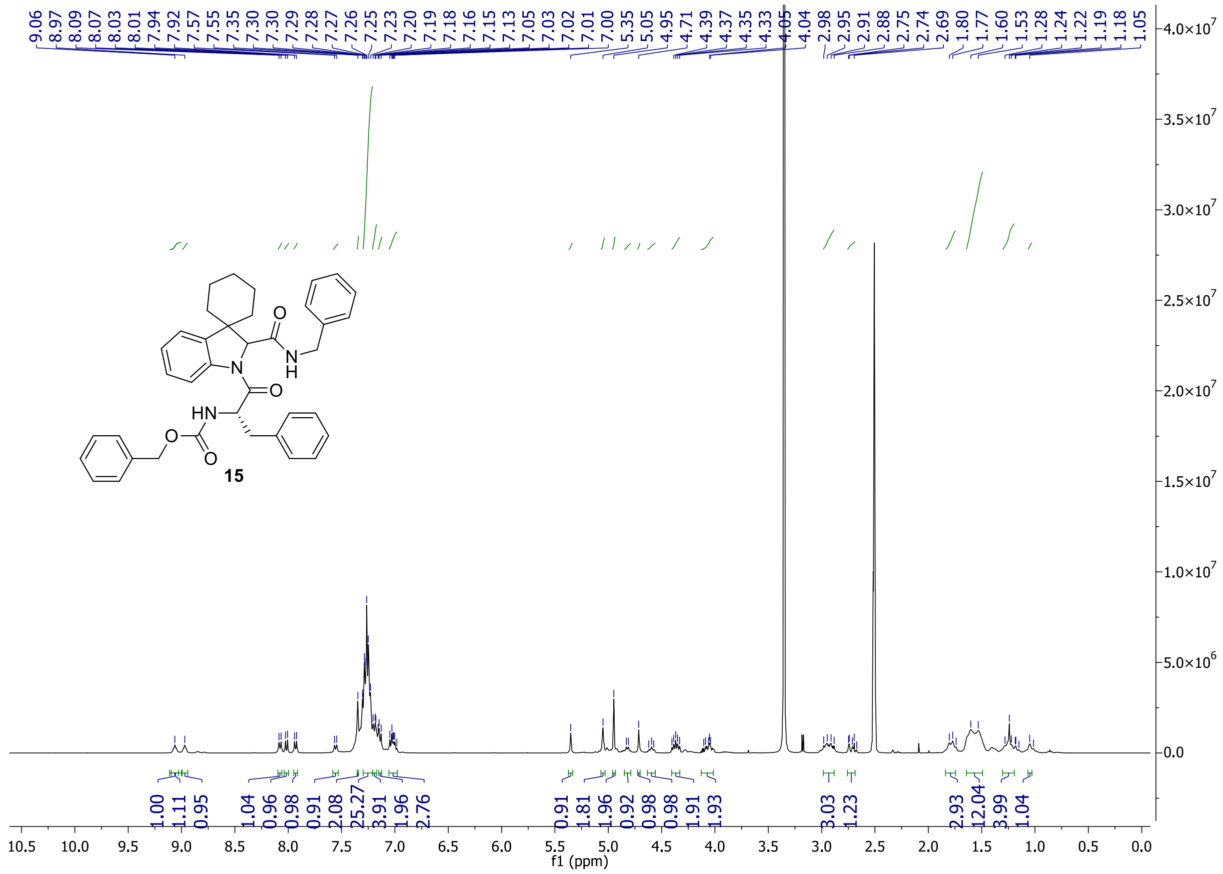
f1 (ppm)
15

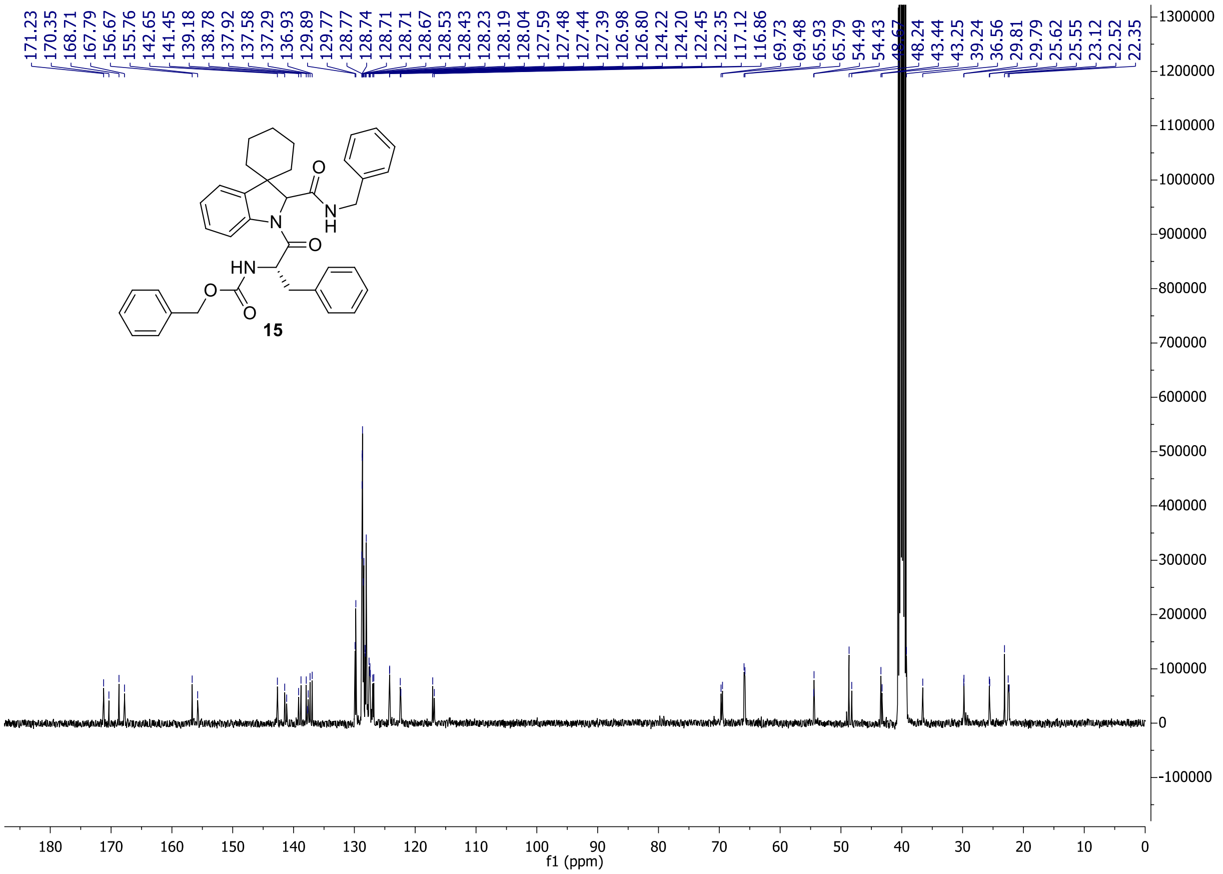

171.23
170.35
168.71
167.79
156.67
155.76
142.65
141.45
139.18
138.78
137.92
137.58
137.29
136.93
129.89
129.77
128.77
128.74
128.71
128.71
128.67
128.53
128.43
128.23
128.19
128.04
127.59
127.48
127.44
127.39
126.98
126.80
124.22
124.20
122.45
122.35
117.12
116.86
69.73
69.48
65.93
65.79
54.49
54.43
48.67
48.24
43.44
43.25
39.24
36.56
29.81
29.79
25.62
25.55
23.12
22.52
22.35
O
N
H
N
O
HN
O
O
15
1300000
1200000
1100000
1000000
900000
800000
700000
600000
500000
400000
300000
200000
100000
0
-100000
180
170
160
150
140
130
120
110
100
90
80
70
60
50
40
30
20
10
0
f1 (ppm)

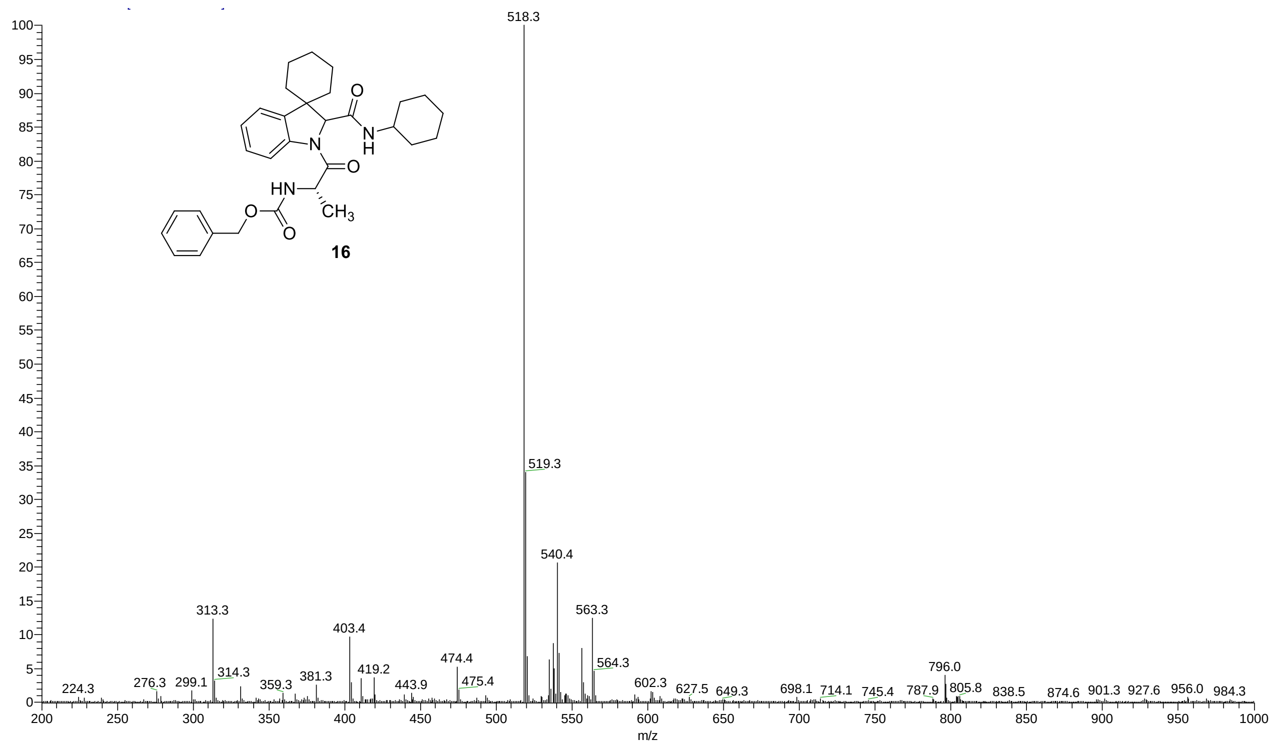
518.3
519.3
540.4
563.3
564.3
313.3
314.3
403.4
419.2
474.4
475.4
224.3
276.3
299.1
359.3
381.3
443.9
602.3
627.5
649.3
698.1
714.1
745.4
787.9
796.0
805.8
838.5
874.6
901.3
927.6
956.0
984.3
m/z
16

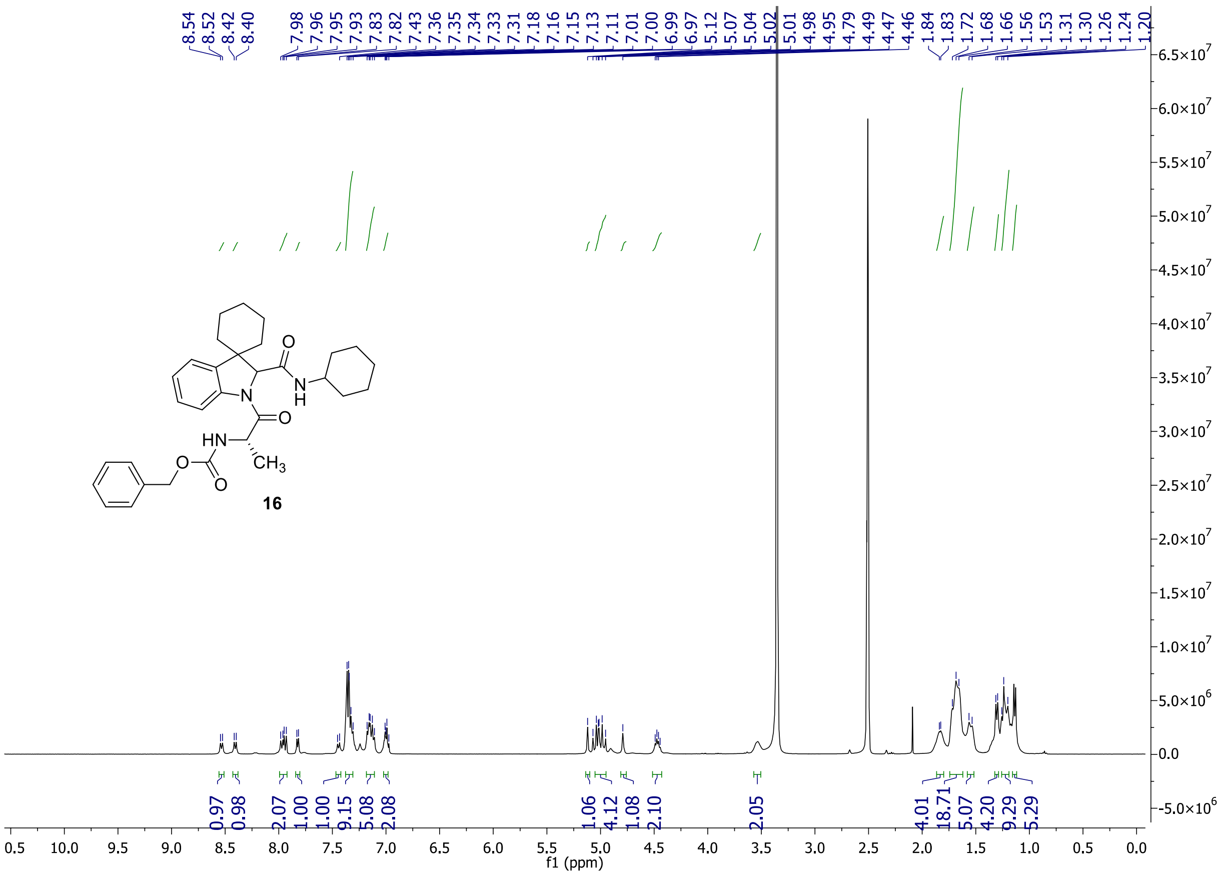
8.54 8.52 8.42 8.40 7.98 7.96 7.95 7.93 7.83 7.82 7.43 7.36 7.35 7.34 7.33 7.31 7.18 7.16 7.15 7.13 7.11 7.01 7.00 6.99 6.97 5.12 5.07 5.04 5.02 5.01 4.98 4.95 4.79 4.49 4.47 4.46 1.84 1.83 1.72 1.68 1.66 1.56 1.53 1.31 1.30 1.26 1.24 1.20
0.97 0.98 2.07 1.00 1.00 9.15 5.08 2.08 1.06 4.12 1.08 2.10 2.05 4.01 18.71 5.07 4.20 9.29 5.29
f1 (ppm)
16

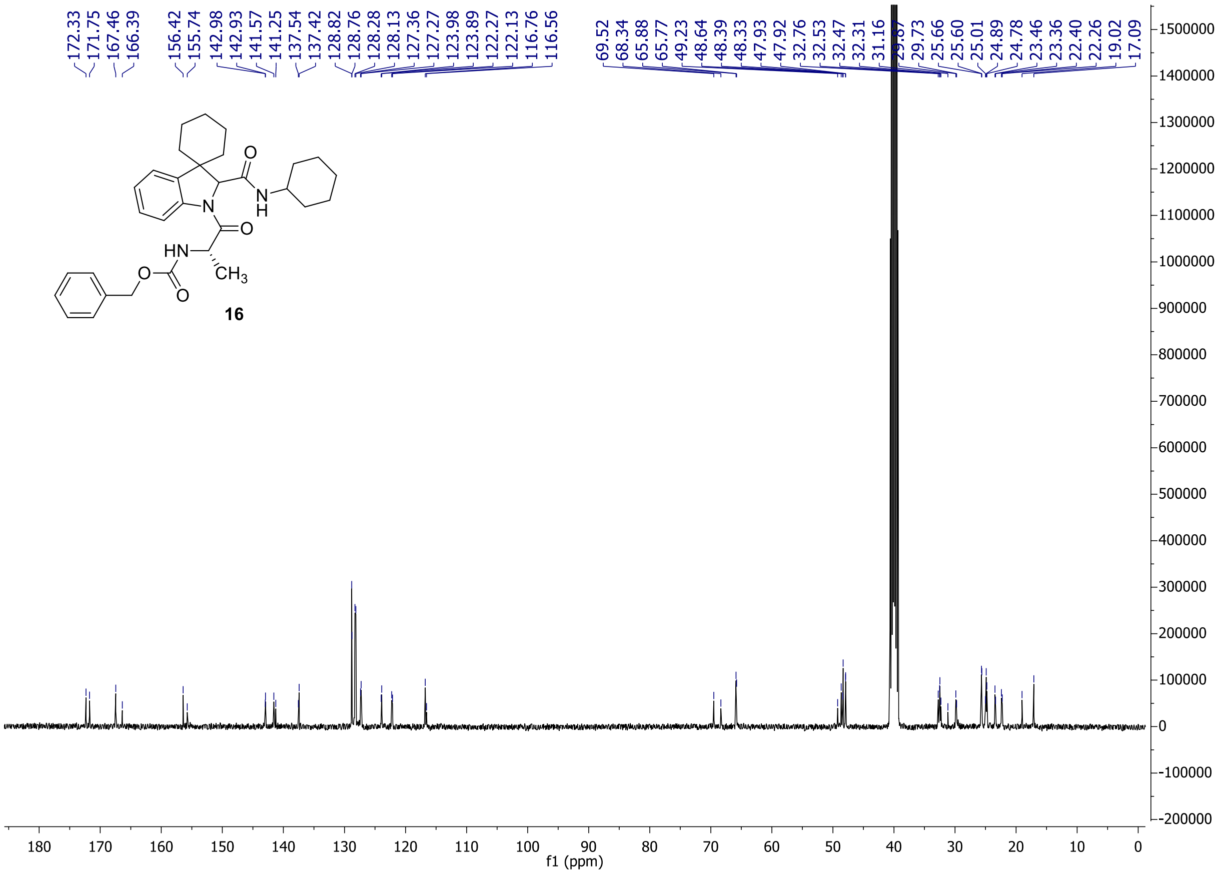
172.33
171.75
167.46
166.39
156.42
155.74
142.98
142.93
141.57
141.25
137.54
137.42
128.76
128.82
128.28
128.13
127.36
127.27
123.98
123.89
122.27
122.13
116.76
116.56
69.52
68.34
65.88
65.77
49.23
48.64
48.39
48.33
47.93
47.92
32.76
32.53
32.47
32.31
31.16
29.87
29.73
25.66
25.60
25.01
24.89
24.78
23.46
23.36
22.40
22.26
19.02
17.09
O
N
H
N
O
HN
CH3
O
O
16
f1 (ppm)

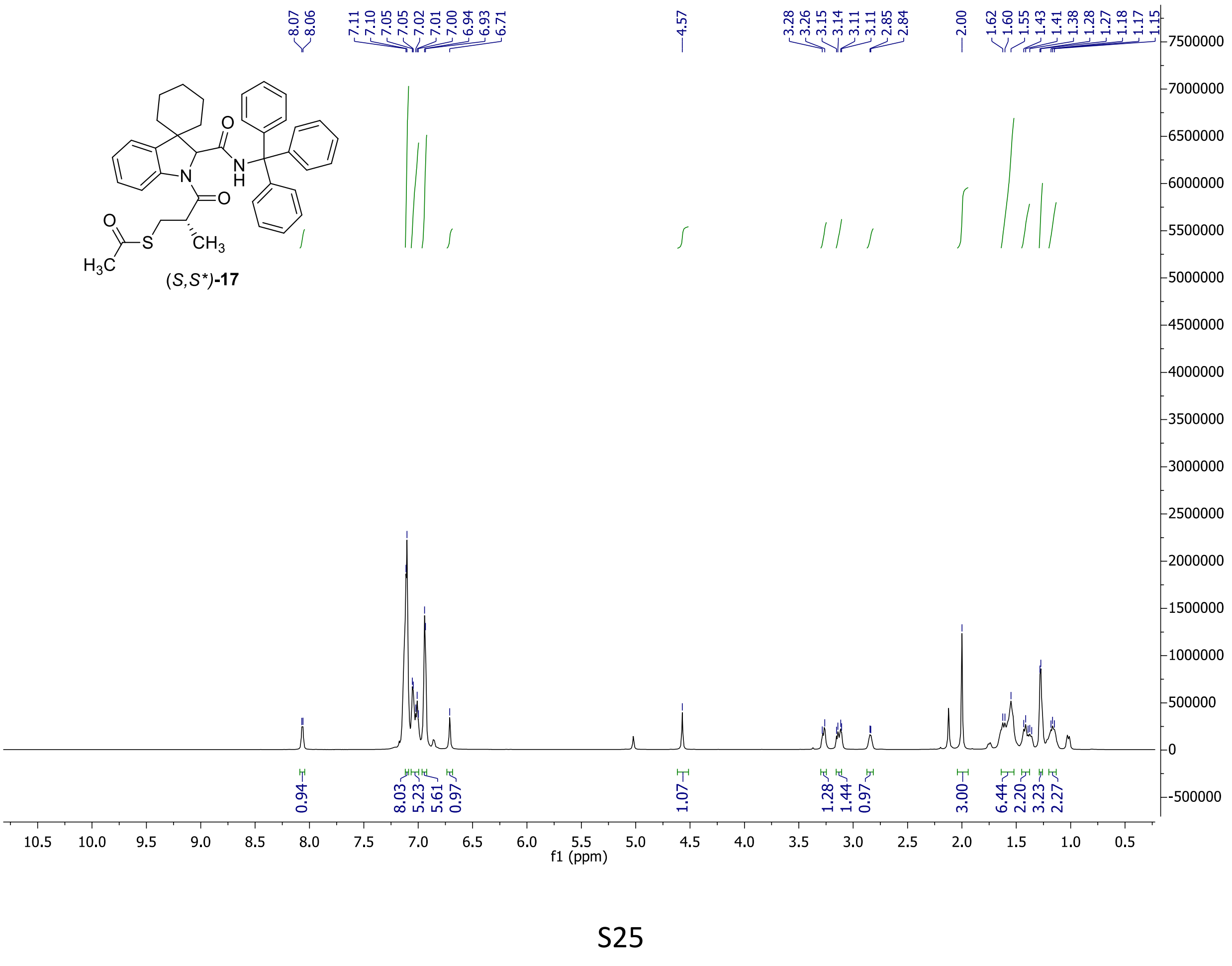
8.07
8.06
7.11
7.10
7.05
7.05
7.02
7.01
7.00
6.94
6.93
6.71
4.57
3.28
3.26
3.15
3.14
3.11
3.11
2.85
2.84
2.00
1.62
1.60
1.55
1.43
1.41
1.38
1.28
1.27
1.18
1.17
1.15
O
N
H
N
O
O
S
CH3
H3C
(S,S*)-17
0.94
8.03
5.23
5.61
0.97
1.07
1.28
1.44
0.97
3.00
6.44
2.20
3.23
2.27
f1 (ppm)

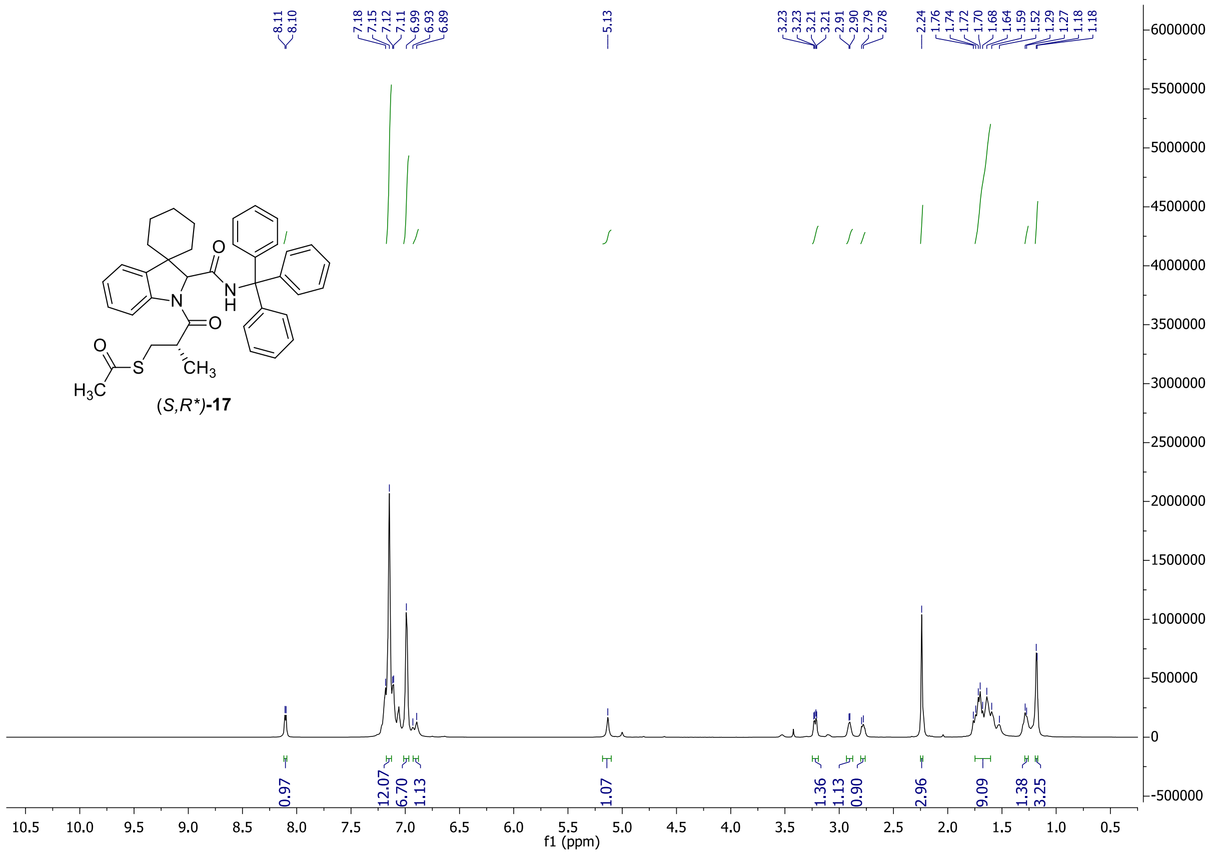
8.11
8.10
7.18
7.15
7.12
7.11
6.99
6.93
6.89
5.13
3.23
3.23
3.21
3.21
2.91
2.90
2.79
2.78
2.24
1.76
1.74
1.72
1.70
1.68
1.64
1.59
1.52
1.29
1.27
1.18
1.18
(S,R*)-17
0.97
12.07
6.70
1.13
1.07
1.36
1.13
0.90
2.96
9.09
1.38
3.25
f1 (ppm)

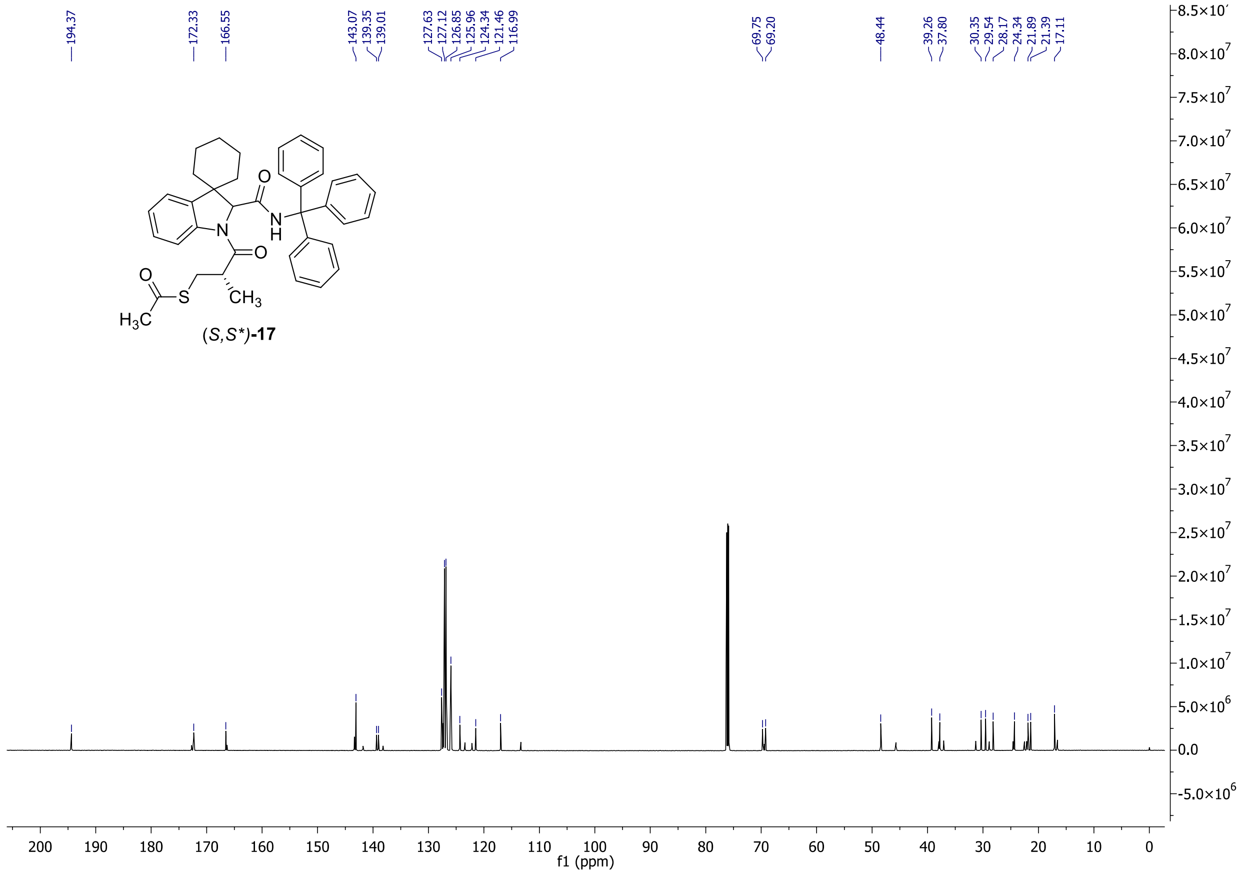
194.37
172.33
166.55
143.07
139.35
139.01
127.63
127.12
126.85
125.96
124.34
121.46
116.99
69.75
69.20
48.44
39.26
37.80
30.35
29.54
28.17
24.34
21.89
21.39
17.11
(S,S*)-17
f1 (ppm)

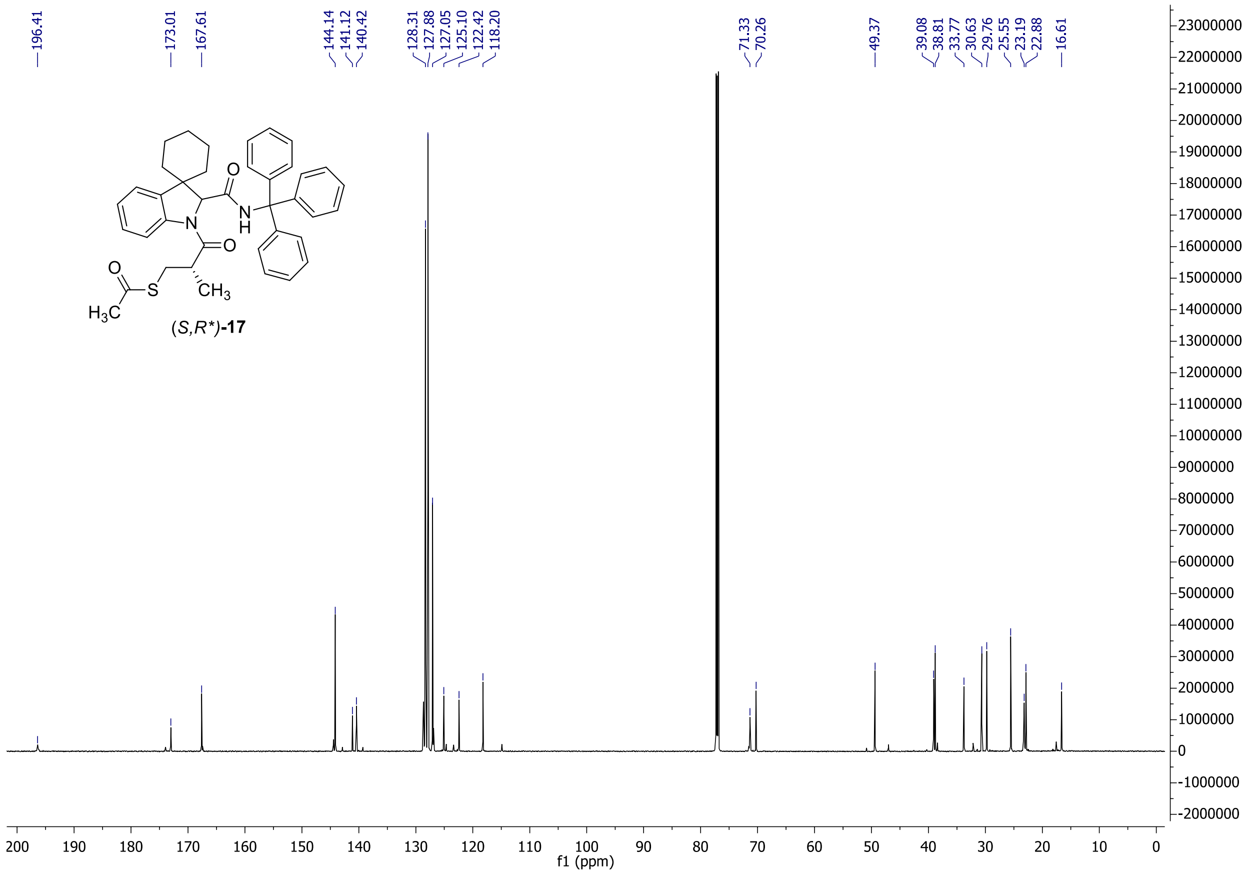

HPLC spectrum of the compound (*S,S**)-8

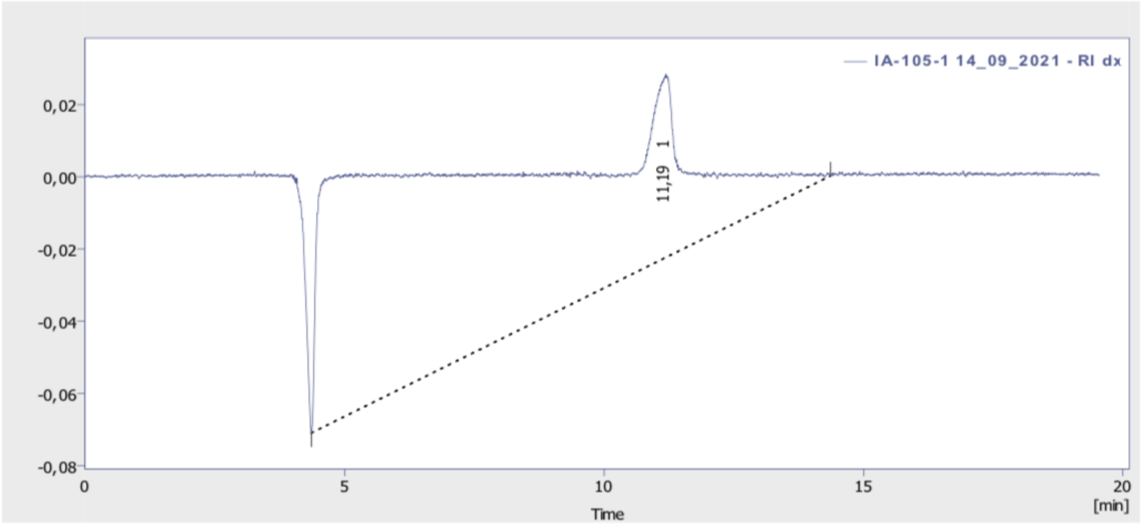

HPLC spectrum of the compound (*S,R**)-**8**

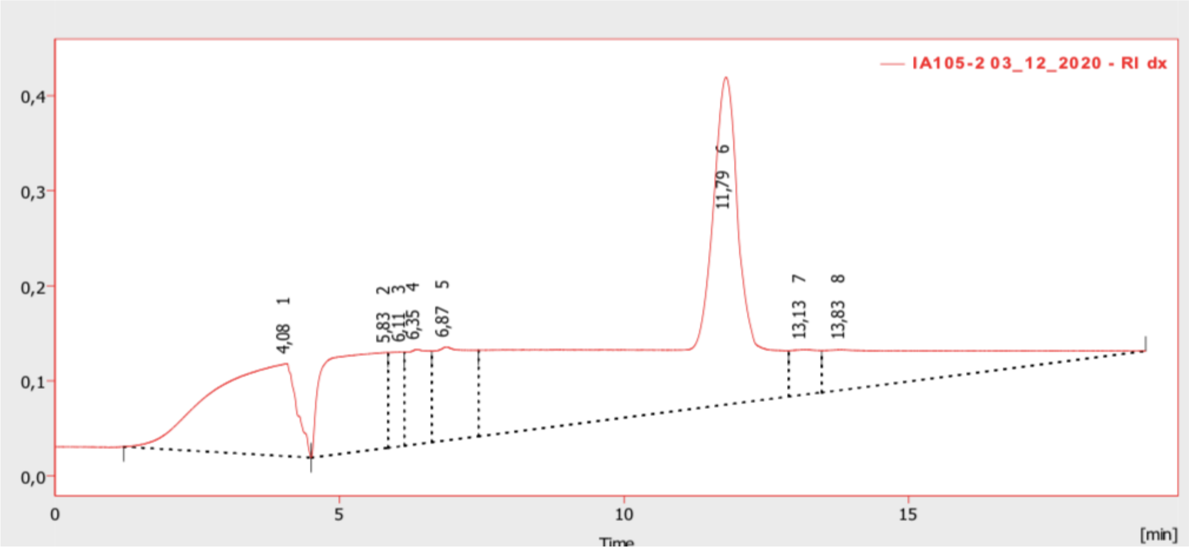

HPLC spectrum of the compound (*S,R**)-**9**

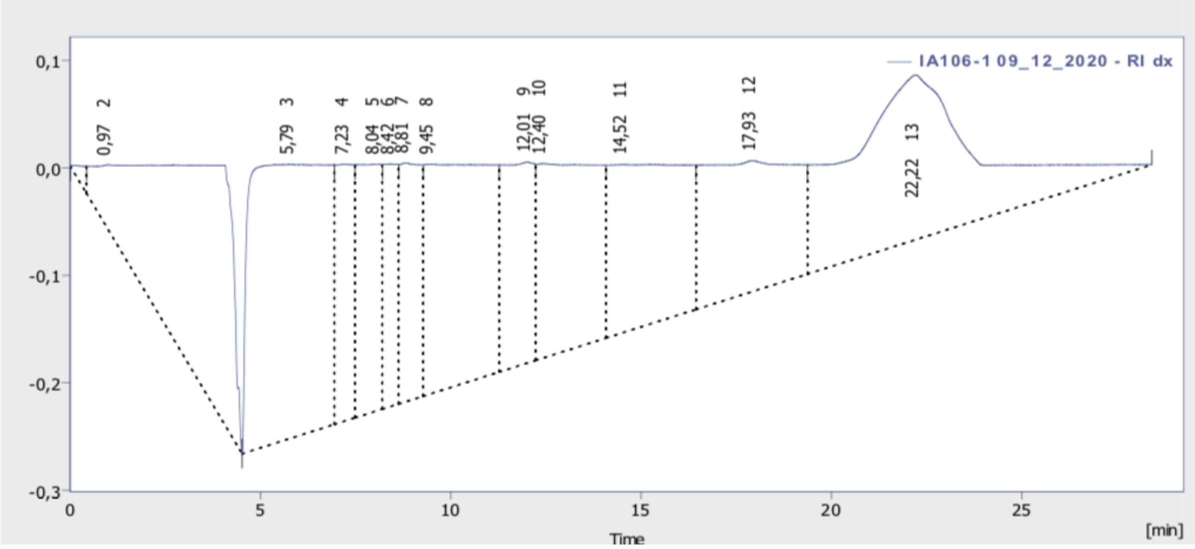

HPLC spectrum of the compound (*S,S**)-**9**

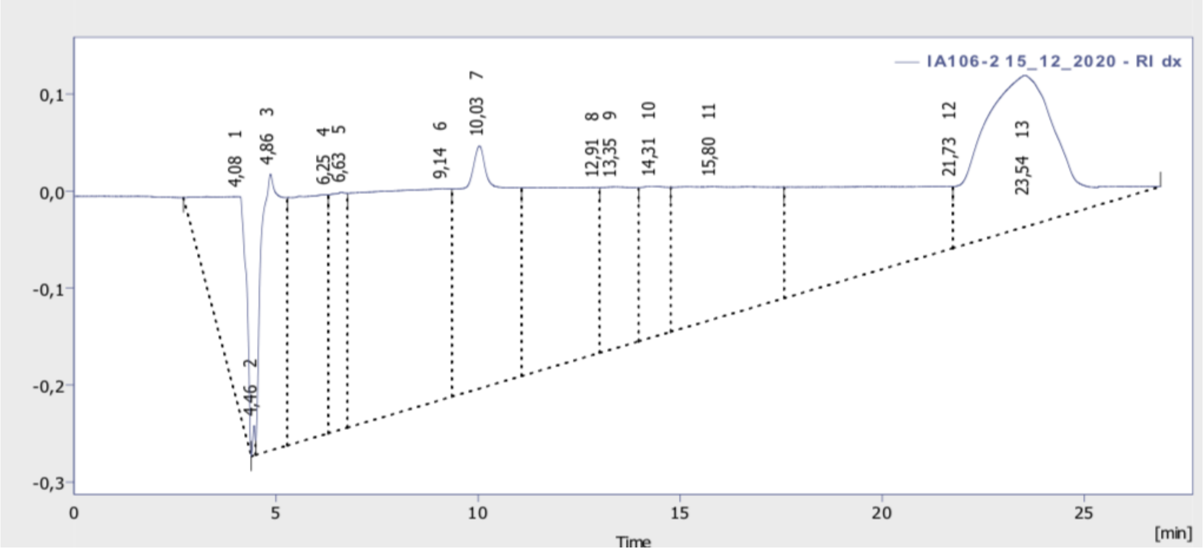

HPLC spectrum of the diastereoisomeric mixture (compound **10**)

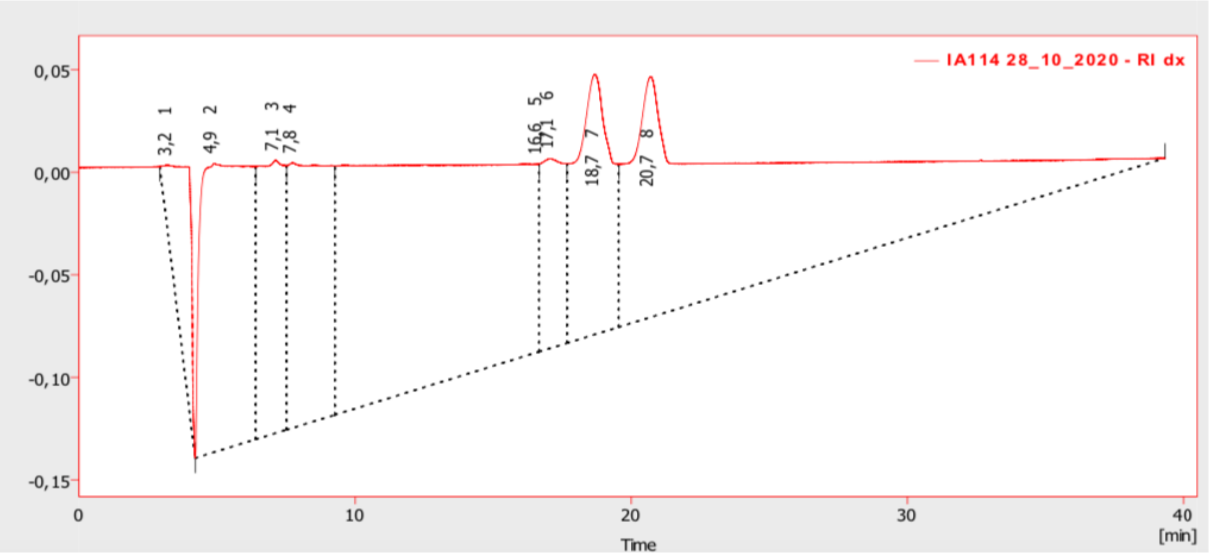

HPLC spectrum of the diastereoisomeric mixture (compound **11**)

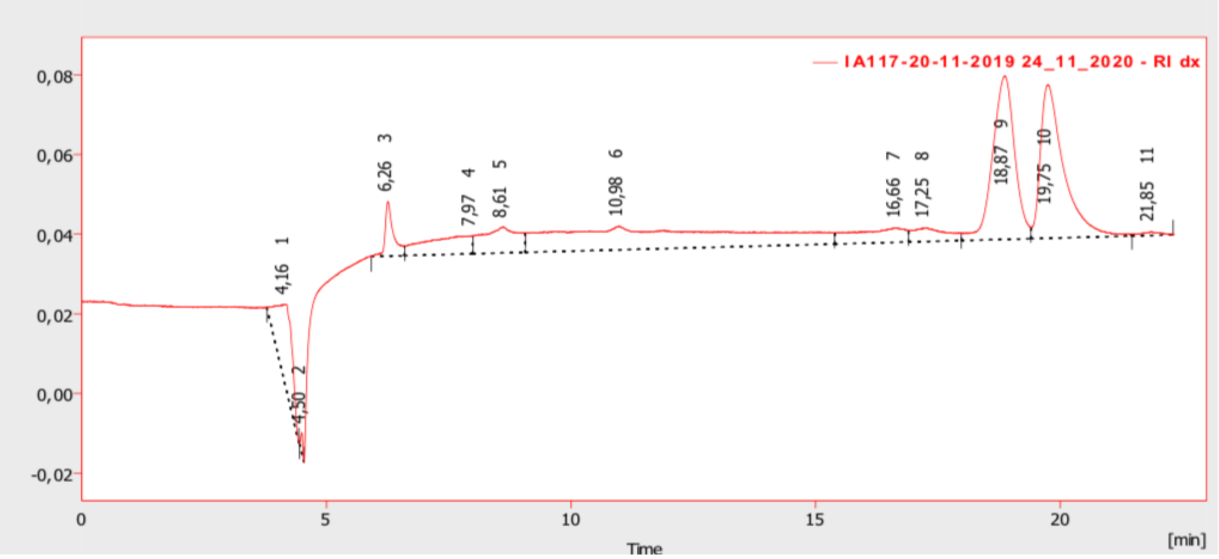

HPLC spectrum of the diastereoisomeric mixture (compound **12**)

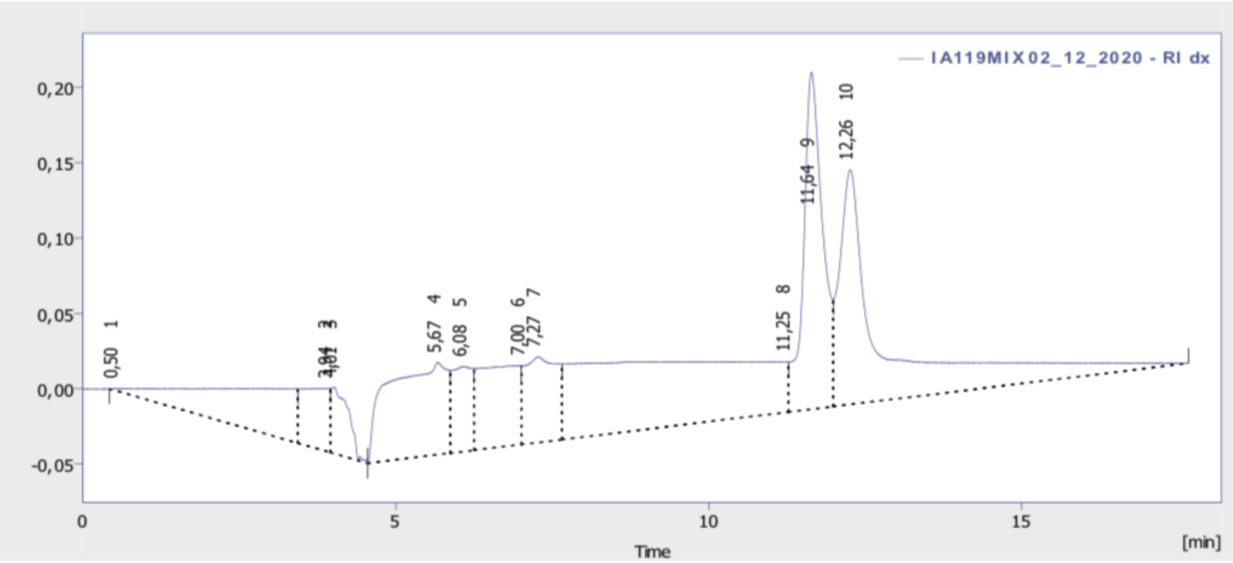

HPLC spectrum of the diastereoisomeric mixture (compound **12**)

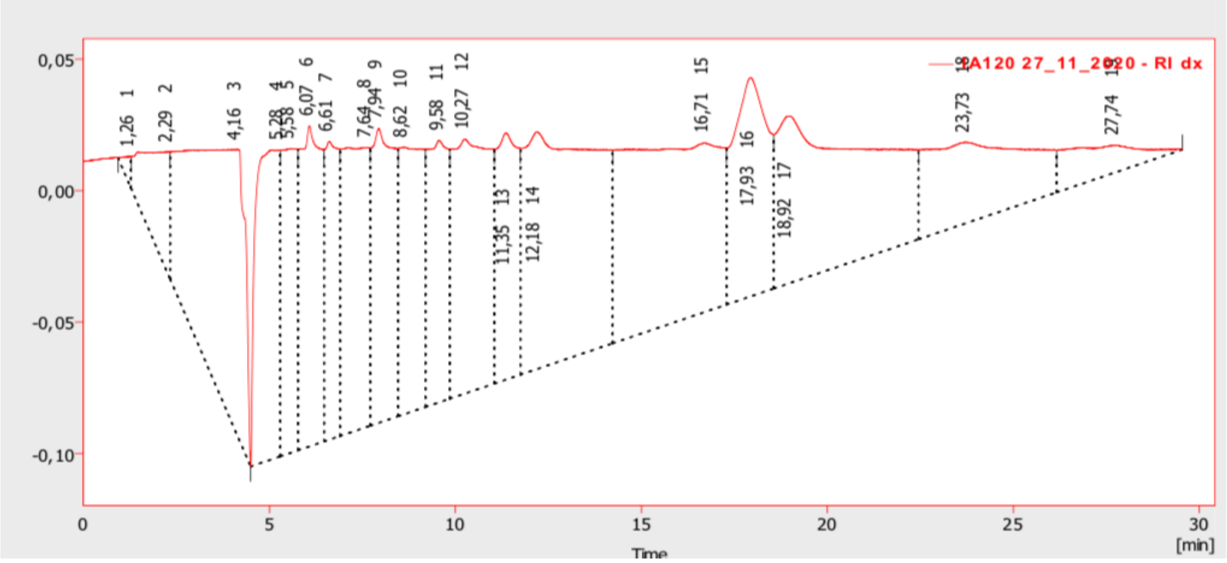

HPLC spectrum of the compound (*S,S**)-**13**

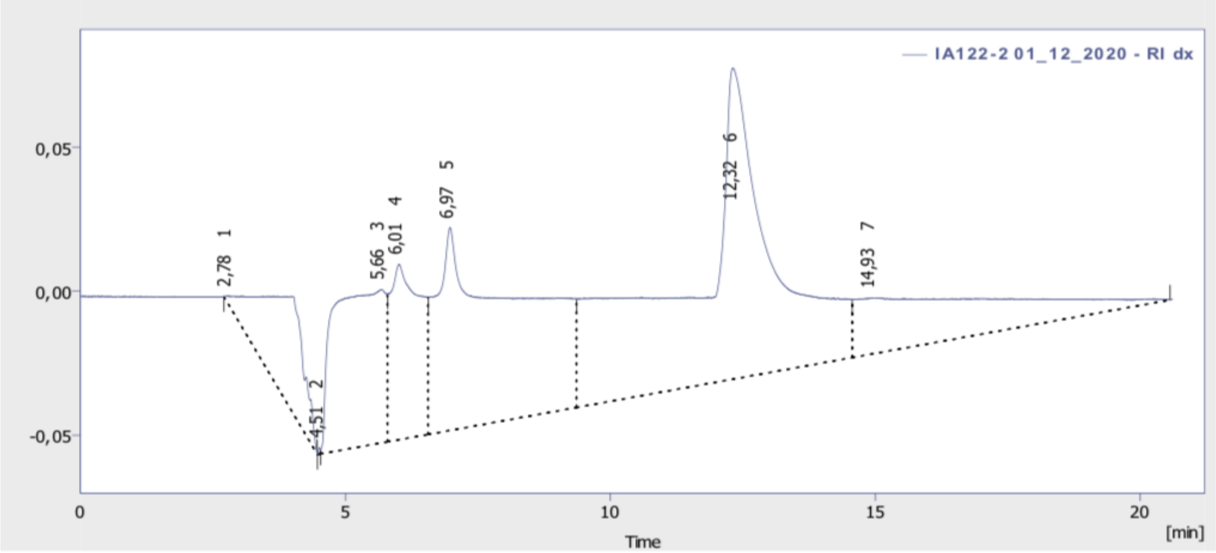

HPLC spectrum of the compound (*S,R**)-**13**

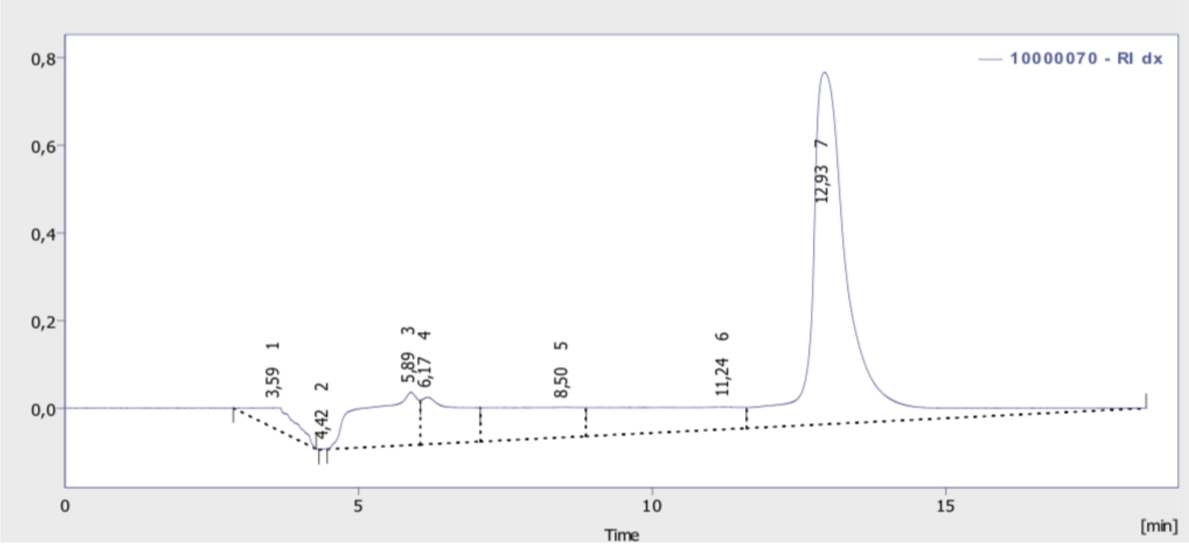

HPLC spectrum of the compound **15**

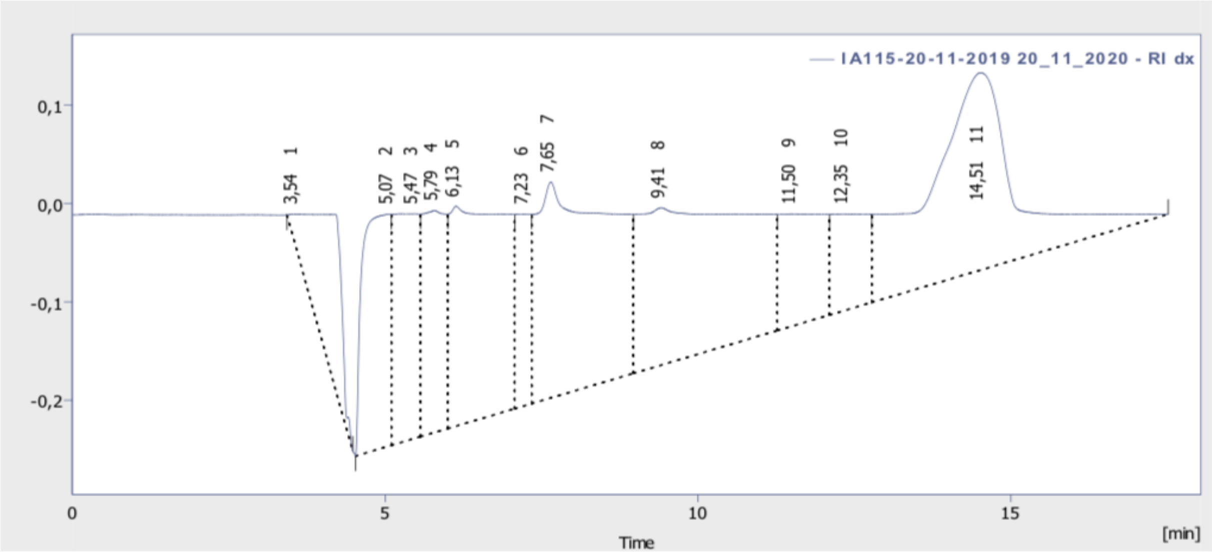

HPLC spectrum of the compound **16**

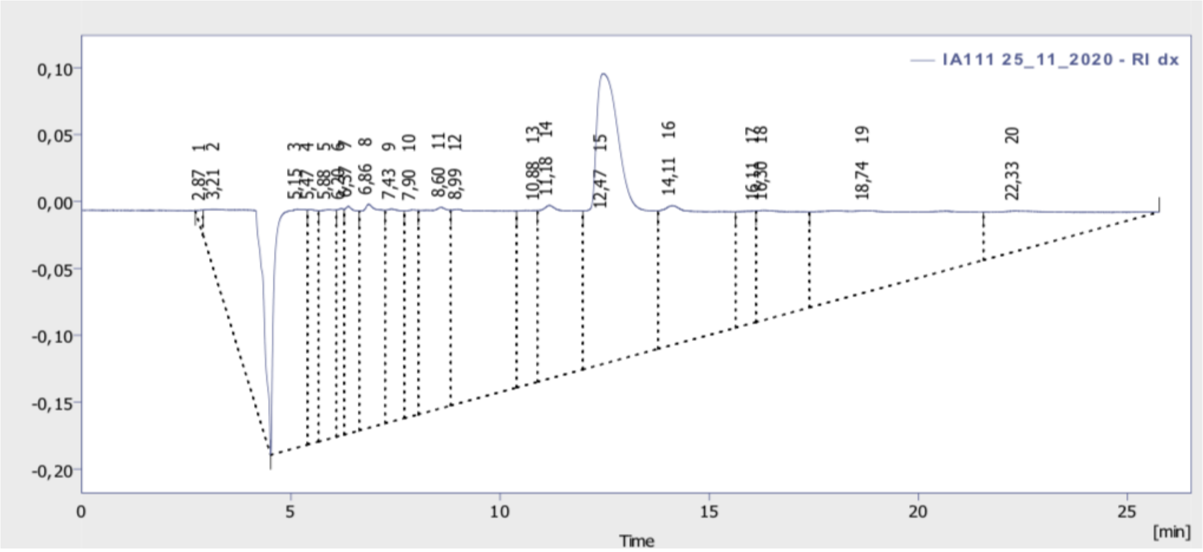

HPLC spectrum of the diastereoisomeric mixture (compound **17**)

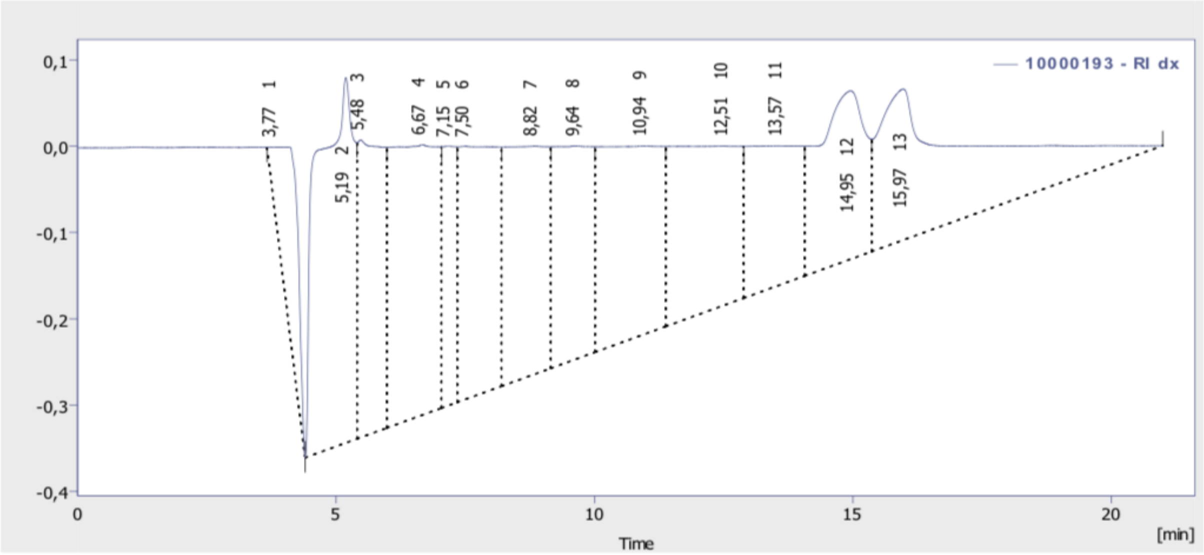

Supplement: Supplementary file 1 — Supporting Information [file CMDC-16-3795-s001.pdf]
